# A daily temperature rhythm in the human brain predicts survival after brain injury

**DOI:** 10.1093/brain/awab466

**Published:** 2022-06-13

**Authors:** Nina M Rzechorzek, Michael J Thrippleton, Francesca M Chappell, Grant Mair, Ari Ercole, Manuel Cabeleira, The CENTER-TBI High Resolution ICU (HR ICU) Sub-Study Participants and Investigators, Jonathan Rhodes, Ian Marshall, John S O’Neill

**Affiliations:** MRC Laboratory of Molecular Biology, Cambridge CB2 0QH, UK; Edinburgh Imaging (Royal Infirmary of Edinburgh) Facility, Edinburgh EH16 4SA, UK; Centre for Clinical Brain Sciences, University of Edinburgh, Edinburgh EH16 4SB, UK; Centre for Clinical Brain Sciences, University of Edinburgh, Edinburgh EH16 4SB, UK; Edinburgh Imaging (Royal Infirmary of Edinburgh) Facility, Edinburgh EH16 4SA, UK; Centre for Clinical Brain Sciences, University of Edinburgh, Edinburgh EH16 4SB, UK; Division of Anaesthesia, University of Cambridge, Box 93 Addenbrooke’s Hospital, Cambridge CB2 0QQ, UK; Division of Neurosurgery, Department of Clinical Neurosciences, University of Cambridge, Box 167, Cambridge Biomedical Campus, Addenbrooke’s Hospital, Cambridge CB2 0QQ, UK; Department of Anaesthesia, Critical Care and Pain Medicine, NHS Lothian, Room No. S8208 (2nd Floor), Royal Infirmary of Edinburgh, Edinburgh EH16 4SA, UK; Edinburgh Imaging (Royal Infirmary of Edinburgh) Facility, Edinburgh EH16 4SA, UK; Centre for Clinical Brain Sciences, University of Edinburgh, Edinburgh EH16 4SB, UK; MRC Laboratory of Molecular Biology, Cambridge CB2 0QH, UK

**Keywords:** brain temperature, brain thermometry, daily, brain injury, mortality

## Abstract

Patients undergo interventions to achieve a ‘normal’ brain temperature; a parameter that remains undefined for humans. The profound sensitivity of neuronal function to temperature implies the brain should be isothermal, but observations from patients and non-human primates suggest significant spatiotemporal variation. We aimed to determine the clinical relevance of brain temperature in patients by establishing how much it varies in healthy adults.

We retrospectively screened data for all patients recruited to the Collaborative European NeuroTrauma Effectiveness Research in Traumatic Brain Injury (CENTER-TBI) High Resolution Intensive Care Unit Sub-Study. Only patients with direct brain temperature measurements and without targeted temperature management were included. To interpret patient analyses, we prospectively recruited 40 healthy adults (20 males, 20 females, 20–40 years) for brain thermometry using magnetic resonance spectroscopy. Participants were scanned in the morning, afternoon, and late evening of a single day.

In patients (*n* = 114), brain temperature ranged from 32.6 to 42.3°C and mean brain temperature (38.5 ± 0.8°C) exceeded body temperature (37.5 ± 0.5°C, *P* < 0.0001). Of 100 patients eligible for brain temperature rhythm analysis, 25 displayed a daily rhythm, and the brain temperature range decreased in older patients (*P* = 0.018). In healthy participants, brain temperature ranged from 36.1 to 40.9°C; mean brain temperature (38.5 ± 0.4°C) exceeded oral temperature (36.0 ± 0.5°C) and was 0.36°C higher in luteal females relative to follicular females and males (*P* = 0.0006 and *P* < 0.0001, respectively). Temperature increased with age, most notably in deep brain regions (0.6°C over 20 years, *P* = 0.0002), and varied spatially by 2.41 ± 0.46°C with highest temperatures in the thalamus. Brain temperature varied by time of day, especially in deep regions (0.86°C, *P* = 0.0001), and was lowest at night. From the healthy data we built HEATWAVE—a 4D map of human brain temperature. Testing the clinical relevance of HEATWAVE in patients, we found that lack of a daily brain temperature rhythm increased the odds of death in intensive care 21-fold (*P* = 0.016), whilst absolute temperature maxima or minima did not predict outcome. A warmer mean brain temperature was associated with survival (*P* = 0.035), however, and ageing by 10 years increased the odds of death 11-fold (*P* = 0.0002).

Human brain temperature is higher and varies more than previously assumed—by age, sex, menstrual cycle, brain region, and time of day. This has major implications for temperature monitoring and management, with daily brain temperature rhythmicity emerging as one of the strongest single predictors of survival after brain injury. We conclude that daily rhythmic brain temperature variation—not absolute brain temperature—is one way in which human brain physiology may be distinguished from pathophysiology.

## Teaser

Temperature-based treatment after brain injury is controversial because ‘normal’ human brain temperature (*T*_Br_) remains undefined. Building a 4D human *T*_Br_ map, Rzechorzek *et al.* show that *T*_Br_ exceeds body temperature by 2–3°C, and varies by age, sex, brain region and time. Loss of a daily *T*_Br_ rhythm reliably predicts death after TBI.


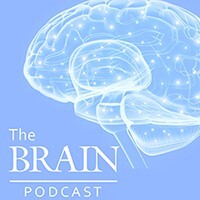


*For the podcast associated with this article, please visit* https://academic.oup.com/brain/pages/podcast

## Introduction

Abnormal temperature has been recognized as a sign of disease for more than two millennia.^1^ Both the temporal and spatial dynamics of temperature contain additional diagnostic information, exemplified by disrupted circadian rhythms, and local warming at sites of injury or infection.^2–9^ Brain temperature (*T*_Br_) is rarely measured directly since invasive methods are required; in practice, it is assumed to match the body core, overlooking the clinical importance of brain-specific measurements. Brain cell function is unequivocally temperature-dependent however,^10,11^ and it is accepted that absolute *T*_Br_, its relationship to body temperature (*T*_Bo_), and the apparent temperature-sensitivity of brain tissue are frequently altered following injury.^3,7,8,12,13^ Indeed, our understanding of human *T*_Br_ has largely been informed by studies of brain-injured patients, where intracranial probes allow precise (±0.1–0.3°C), direct measurement from a single brain locus.^14,15^ The clinical relevance of these data is obscured entirely by the lack of a comprehensive reference dataset; application of targeted temperature management (TTM) in neurocritical care thus remains highly controversial.

Despite its irrefutable clinical value, the normal range of human *T*_Br_ is unknown. Whilst the temperature-dependence of brain function has perpetuated the assumption that *T*_Br_ is maintained within a very narrow range, several lines of evidence suggest that healthy *T*_Br_ may vary over time, and between brain regions.^4,5,7,9,12,13,16–20^ For example, human core *T*_Bo_ is 1–2°C lower during sleep, when cerebral blood flow (CBF) is also ∼20% higher^21,22^; therefore, brain heat removal should be more efficient at night than during the day. Moreover, direct measurements in non-human primates show that deep brain structures are warmer than its surface and that *T*_Br_ varies at least as much as *T*_Bo_ across a 24-h period.^19,20^

Deviations of *T*_Br_ may have transformative diagnostic and/or prognostic utility for acute and chronic brain disorders, but only if these deviations can be distinguished from physiological variation over time.^23^ With magnetic resonance spectroscopy (MRS), spatially resolved *T*_Br_ data can now be obtained non-invasively.^14^ Brain thermometry has proven to be a powerful research application of MRS but, with respect to healthy humans, it has only been used in studies that were not designed to explore time-of-day variation in temperature (Supplementary Table 1). We sought to undertake an evidence-driven appraisal of the clinical value of *T*_Br_ in patients by establishing its spatiotemporal variation in healthy adults. We hypothesized that healthy human *T*_Br_ would vary diurnally (in the manner expected for a daytime active mammal), and that disruption of daily temperature variation would be associated with outcome after traumatic brain injury (TBI).

## Materials and methods

Reporting adheres to STROBE guidelines (Supplementary material). All results are reported ± standard deviation (SD) unless otherwise stated. See Supplementary Box 1 for definitions of terms and phrases that have a specific meaning within the context of this work, but which may have alternative meanings in other contexts.

### Ethics approval

For TBI patient data analysis, approval was provided by NHS Scotland (14/SS/1086, R&D Department, University Hospitals Division NHS Lothian 2015/0171) for data collected at the Intensive Care Unit, Western General Hospital, Edinburgh, UK. For data extracted from other clinical sites via the CENTER-TBI database, the CENTER-TBI study was conducted in accordance with all relevant laws of the EU if directly applicable or of direct effect and all relevant laws of the country where the recruiting sites were located, including but not limited to, the relevant privacy and data protection laws and regulations (the ‘Privacy Law’), the relevant laws and regulations on the use of human materials, and all relevant guidance relating to clinical studies from time to time in force including, but not limited to, the ICH Harmonised Tripartite Guideline for Good Clinical Practice (CPMP/ICH/135/95) (‘ICH GCP’) and the World Medical Association Declaration of Helsinki entitled ‘Ethical Principles for Medical Research Involving Human Subjects’. Informed consent by the patients and/or the legal representative/next of kin was obtained, accordingly to the local legislations, for all patients recruited in the Core Dataset of CENTER-TBI and documented in the e-CRF. Ethical approval was obtained for each recruiting site. The list of sites, ethical committees, approval numbers and approval dates can be found on the website: https://www.center-tbi.eu/project/ethical-approval. Prospective data collection in healthy volunteers was co-sponsored by the University of Edinburgh and NHS Lothian (R&D Project Number 2019/0133). Ethics approval was obtained from the Academic and Clinical Office for Research Support (ACCORD) Medical Research Ethics Committee (AMREC; Study Number 18-HV-045). All participants provided written informed consent to participate.

### Patient brain temperature

#### Study design and data sources

We conducted a multicentre, retrospective cohort study of TBI patients that had high temporal-resolution *T*_Br_ data collected directly from the brain. Data for all eligible patients were extracted using version 2.0 of the CENTER-TBI dataset, compiled between 2015 and 2017. Additional eligible patients monitored at one of the contributing sites (Intensive Care Unit, Western General Hospital, Edinburgh, UK) were included up to May 2020 and comprised 109 of the 134 eligible patients screened. The Western General Hospital is the tertiary referral centre in South East Scotland for neurosurgical emergencies. Patients with moderate to severe TBI admitted to intensive care requiring intubation, sedation, and intracranial pressure management also received brain oxygen tension and temperature monitoring using the Integra Licox system (Integra). Patients were managed in accordance with Brain Trauma Foundation guidelines.^24^ Patients were either admitted directly to intensive care or following surgical intervention for mass lesions. *T*_Br_ was measured via a thermistor, inserted into the brain parenchyma via a dedicated bolt placed via a burr hole (Integra Neurosciences). The bolt was placed so that the thermistor was inserted into frontal white matter at a tissue depth of around 18 mm below the dura; for diffuse injuries this was into the non-dominant hemisphere. When the main injury was focal, the bolt was placed on the side of maximal injury, unless this would place the monitors into non-viable tissue. High temporal-resolution physiological data were recorded at a minimum of 1-min intervals to either a bedside computer running ICU Pilot software (CMA) or to a Moberg neuromonitoring system (Moberg Research Inc.). Data were collected continuously (except for interruptions due to CT scanning or surgical intervention) and until intracranial pressure monitoring was no longer required, or the patient died. Data for the CENTER-TBI study were collected through the Quesgen e-CRF (Quesgen Systems Inc.), hosted on the INCF platform and extracted via the INCF Neurobot tool (INCF, Sweden). For patient monitoring and data collection in the High-Resolution repository, the ICM+ platform (University of Cambridge, UK) and/or Moberg Neuromonitoring system (Moberg Research Inc., USA) were used. For *T*_Bo_, the primary method of measurement was documented in 26 of 134 screened patients and included tympanic (21), bladder (3), external axillary (1) and nasopharyngeal (1). Secondary sites included rectal, external axillary, oesophageal and skin.

#### Temperature data processing

Four inclusion criteria levels were applied to ensure that sufficient temperature data from the brain and/or body were available to assess for a daily rhythm (Box 1), and that any data potentially affected by TTM protocols were excluded. Analysis of patient temperature data was blinded to outcome. Data from the first 2 h of monitoring were excluded from the analysis to ensure the results were not influenced by the time required for the electrode to stabilize. Raw data processing was performed in Excel™ to identify any gaps in the time series, exclude artefactual data (e.g., impossible negative values either side of data gaps) and define the analysis window. Analysis of patient temperature data served to determine whether a daily rhythm was present, agnostic to any relationship with the environmental timing cues. Entrainment by environmental cues was not assumed, since the amplitude of these cues may be diminished in the critical care setting, and their transduction might be impaired in TBI patients. Temperature data were first visualized in GraphPad Prism version 8.2 and assessed for the presence of daily rhythmicity. Visual analyses were validated by testing all datasets with a battery of rhythm-detection algorithms using GraphPad Prism, BioDare2 (biodare2.ed.ac.uk)^25^ and the Harmonic Regression package in R.^26^ To be categorized as having a daily rhythm, the patient’s temperature pattern need not be aligned with the day-night cycle, but it had to meet both of the following criteria: (i) a period length of ∼22–26 h in at least part (but not necessarily all) of the time series as determined by visual analysis of the raw data in GraphPad Prism; and (ii) a period length of 22–26 h as determined by period analysis in (a) cosinor analysis in GraphPad Prism and/or (b) statistically significant output from Harmonic Regression in R and/or (c) BioDare2.

Box 1 **Inclusion criteria for retrospective analysis of temperature data from TBI patients**

**Level A: Criteria for extracting maximum and minimum daily brain and/or body temperatures**

- Known sex
- Known age
- Minimum 24 h of temperature data collection under ‘constant’ conditions. The first data-point recorded in the intensive care setting that exceeds the minimum recorded temperature from that patient in the absence of TTM will be taken as the start point (to exclude low temperature points surrounding insertion of probe or those relating to patient hypothermia on arrival in intensive care)
- For data where only the maximum and minimum daily *T*_Bo_ (and in some cases *T*_Br_) are recorded with their respective times, a minimum of two days’ worth of data is needed
- If TTM was applied, only data relating to time preceding TTM or after the first inflection of data after cessation of TTM can be used and must meet the above requirements for minimum time length in the absence of TTM
- When extracting the time of the minimum and maximum temperature point, the first occurrence of that specific temperature point under intensive care ‘constant’ conditions will be selected

**Level B: Additional criteria for performing daily rhythmic temperature analyses**

- Minimum hourly *T*_Bo_ or *T*_Br_ data with *T*_Br_ data extracted via intracranial probe (standard depth and positioning in cortical white matter) recorded continuously over a minimum of 36 h. The same rules as above apply in relation to TTM.
- Ideally minimum hourly data of another matched parameter [intracranial pressure, partial pressure of brain oxygen (PbTO_2_), mean arterial pressure] with expected daily rhythm

**Level C: Additional criteria for correlation with outcome**

- Mortality/survival in intensive care
- Ideally, a Glasgow Outcome Scale Extended (GOSE) score at 3 and/or 6 months (imputed where necessary)

**Level D: Additional criteria for correlation with injury severity**

- One of more of the following parameters: presence of pupillary light reflex in one/both/no eyes; Glasgow Coma Scale (GCS) score, Glasgow Coma Scale motor response (GCSM) score
- Ideally injury type (focal/diffuse; from CT scoring) and/or severity [International Mission for Prognosis and Analysis of Clinical Trials in TBI (IMPACT) imputed GCS] on admission to Study Hospital and/or Therapy Intensity Level score (including individual components of this)
- Ideally site of probe insertion for focal injury (ipsilateral or contralateral to injury—to be determined using CT/MRI images if available)

In GraphPad Prism, period results were only considered valid if a cosinor curve fit was significantly preferred over a straight line. When using the Harmonic Regression package, the period length term (Tau) of the model to test for was set to 24 h. In BioDare2, period analysis was performed using six different algorithms. A full description of these algorithms can be found at https://biodare2.ed.ac.uk/documents/period-methods. All patient temperature analyses were blinded to patient outcome and a detailed description of how patient temperature datasets were handled using the approaches summarized above can be found in Supplementary material and Supplementary Fig. 1. Results for each patient are presented in the Supplementary material.

### Healthy brain temperature

#### Study design and recruitment

We conducted a prospective, single-site, cohort study in healthy adults, controlled for age, sex, body mass index (BMI), menstrual cycle phase, seasonal variation and individual chronotype. Our primary objective was to determine whether healthy *T*_Br_ varies by time of day. Our secondary objectives were to compare variability in brain and oral temperatures, to test for differences between males and luteal-phase females and to explore brain-regional changes in *T*_Br_ with time. We hypothesized that *T*_Br_ would (i) exceed and vary more than oral temperature across the day; (ii) be higher in luteal females relative to males; and (iii) increase with increasing brain tissue depth. Sample size was estimated for achieving the primary outcome (a change in *T*_Br_ between time points) using a linear mixed model, considering published data on the reliability of MRS brain thermometry in healthy men.^14^ With 36 subjects, and a conservative true diurnal mean *T*_Br_ difference of 0.5°C, we estimated 80% power to detect a statistically significant difference between time points at the 5% significance level. A health-related finding on MRI was the key exclusion criterion and was expected for two volunteers (based on 5% prevalence of health-related findings using high-resolution MRI).^27^ We recruited 40 eligible participants (20 females) for scanning to account for potential withdrawal, exclusion and/or technical scan failure.

Recruitment for our prospective study was based on meeting criteria for our primary outcome (Supplementary Table 2) and was conducted locally using mailshots to University of Edinburgh and NHS staff, social media posts and posters displayed at University of Edinburgh campuses and NHS Lothian hospitals. By completing an online eligibility questionnaire, all prospective participants provided written informed consent for their personal data to be used to schedule consenting interviews and to notify general practitioners of their intention to participate. The questionnaire provided access to inclusion and exclusion criteria, the Participant Information Sheet and Consent to Participate Form (Supplementary material) and Data Protection Information sheet. All participants provided written, informed consent to participate during face-to-face interview conducted by the Chief Investigator (N.M.R.) at the University of Edinburgh. Additional written informed consent was obtained for publication of individual data which, by nature of its distinctive features, could potentially be recognized by participants as their own data. The Study Protocol is presented in the Supplementary material.

#### Prospective data collection

During a consenting interview at the study site, 1 week in advance of scanning, each participant was given a wrist-worn actimeter (ActTrust2, Condor Instruments). Each participant then underwent three identical brain scans in the morning (9–10 am), afternoon (4–5 pm) and late evening (11 pm–midnight) of their scheduled scanning day. Multiple time points spanning >12 h were selected because the human circadian rhythm (body clock) impacts almost every aspect of physiology (Supplementary material).^28–31^ The exact alignment, or phase relationship, between the body clock and the day-night cycle is dictated by individual chronotype, which is determined by genetic and lifestyle factors, and can be derived from longitudinal monitoring of locomotor activity.^32^ To assign scan times to the appropriate part of each participant’s circadian cycle, we determined individual chronotypes using wrist actigraphy to extract the sleep-corrected midpoint of sleep on free (non-work) days (MSF_sc_; Supplementary material).^32^

Height and weight were measured immediately before the morning scan to calculate BMI. Oral temperature was recorded before each scanning session using a digital Clinical Thermometer (S. Brannan & Sons) covered in a single-use Probe Cover (Bunzl Retail & Healthcare Supplies Ltd.) and placed sublingually. Rectal temperature probes were not deemed appropriate since these would discomfort volunteers and impede study recruitment without providing *T*_Bo_ measurements that were likely to be any more meaningful for the interpretation of *T*_Br_ than those collected sub-lingually. Although desirable, continuous core *T*_Bo_ measurement using ingestible telemetric sensors was precluded due to their incompatibility with MRI. Oral temperature measurements also served to exclude any participants with a fever. For females, hormonal influences were controlled through urine-based ovulation testing (ClearBlue^®^) or documenting hormonal contraception type. We aimed to scan females during the luteal phase of their natural menstrual cycle, or on a day when an active combined pill would be taken, or combined patch worn. Females using other forms of contraception (implant or intrauterine device) were excluded. On the day of scanning, food consumption was restricted to 6 am–8 am, 12 noon–2 pm and 6 pm–8 pm; caffeine consumption was restricted to 6 am–8 am and 12 noon–2 pm. Alcohol was strictly prohibited at all times. Participants were asked not to participate in excessive physical activity on the day of scanning. Data collection was limited to a 14-week period between July and October 2019 to avoid daylight savings clock changes and large seasonal variation in environmental light and temperature conditions. Data management procedures are described in Supplementary material.

#### Brain imaging

All brain imaging was conducted at the Edinburgh Imaging (Royal Infirmary of Edinburgh) Facility using a 3-T MAGNETOM Prisma scanner (Siemens Healthcare) with a 32-channel head coil. All participants were screened for MRI contraindications and changed into hospital scrubs for each scanning session—conducted in a temperature-controlled room (target 21.5°C). Room lights were off and the scanner lighting and fan were maintained on their lowest setting. Ear protection was provided and a mirror was attached to the head coil so participants had the choice of closing their eyes or viewing the MRI control room; no visual or acoustic entertainment was provided. At each time point, after whole-brain structural MRI, MRS data were collected from 82 brain locations (voxels). Since the main objective of our study was to determine how human *T*_Br_ varies over the course of a normal day, it was designed to include time points that covered the range of waking hours for most people, without disrupting normal sleep patterns, nor imposing any restrictions on vigilance state. Whilst the protocol was not designed to formally assess vigilance state, we anticipated that some participants might fall asleep during scanning (particularly at the late evening session), and that this might be associated with changes in MRS-derived *T*_Br_.^33^ Participant self-reported sleep was documented at the end of each session so that this could be incorporated into our analysis as a fixed effect. The scanning protocol was well tolerated, with no serious adverse events reported during 7-day follow-up. Further details on the scanning protocol, MRS data processing and the dedicated Study Participant Data Form (Case Report Form) are provided in Supplementary material.

#### Calculation of MRS-derived brain temperature

MRS brain thermometry exploits the fact that the chemical shift of water is exquisitely temperature-dependent (−0.01 ppm/°C), whilst that of the reference metabolite NAA is not.^34^ The chemical shift difference between water and NAA can estimate absolute *T*_Br_ in healthy males with a short-term precision of 0.14°C at 3-T.^14^ *T*_Br_ for each brain tissue voxel in this study was calculated using the following relationship:

(1) $$T_{\mathrm{Br}}=100\times [\mathrm{NAA}\;\mathrm{frequency}\text{–}H_{2}O\;\mathrm{frequency}+2.665]+37$$

where frequency is in parts per million and temperature is in degrees Celsius.

The reliability and accuracy of *T*_Br_ determination using this MRS protocol was thoroughly tested using *in vivo* human and *in vitro* phantom measurements; the latter validated with a magnetic resonance-compatible industrial thermometer that meets international standards.^14^

### Statistical analysis

To determine healthy temperature variation, we applied a linear mixed modelling approach (Supplementary material). The fixed effects (predictors) were specified *a priori* based on published literature describing factors that were most likely to affect body and/or brain temperature in humans and other mammals.^23^ In each case, an upper limit for the number of fixed effects was set to avoid over-fitting each model within the confines of our sample size.^35^ Random effects for intercept and slope were included, allowing participants to have different baseline temperatures and different changes in temperature over time. The models for oral temperature (OralTemp) and *T*_Br_ (BrainTemp) were built as follows:

(2) $$\begin{array}{rl} \mathrm{OralTem}p_{ij} & =[\mathrm{intercept}(\beta 0)+\mathrm{Time}(\beta 1)+\mathrm{Sex}(\beta 2)\\ & \;+\mathrm{EdTemp}(\beta 3)+\mathrm{Age}(\beta 4)+\mathrm{BMI}(\beta 5)]\\ & \;+\varepsilon_{ij}(\mathrm{residuals}\;\mathrm{for}\;\mathrm{subject}\;i\;\mathrm{at}\;\mathrm{time}\;j)\\ & \;+U1_{i}(\mathrm{intercept}\;\mathrm{for}\;\mathrm{subject}\;i)\\ & \;+U2_{i}(\mathrm{slope}\;\mathrm{for}\;\mathrm{subject}\;i\;\mathrm{in}\;\mathrm{relation}\;\mathrm{to}\;\mathrm{Time}) \end{array}$$

where fixed effects include: Time (time of day normalized for chronotype using the ‘time distance’ between the *T*_Oral_ measurement and MSF_sc_ for that participant as a proportion of a linearized unit circle where 0 = MSF_sc_ and 1 = 24 h); Sex (participant biological sex categorized as male, luteal female, or non-luteal female); EdTemp (environmental temperature in Edinburgh on that date and at the time of temperature measurement); Age (participant age on date of temperature measurement); BMI (participant BMI on date of temperature measurement), with random effects for intercept by subject and for slope by subject with respect to time.

(3) $$\begin{array}{rl} \mathrm{BrainTem}p_{ij} & =[\mathrm{intercept}\;(\beta 0)+\mathrm{Time}(\beta 1)+\mathrm{Sex}\;(\beta 2)\\ & \;\;+\;\mathrm{BrainRegion}(\beta 3)+\mathrm{Age}(\beta 4)+\mathrm{Sleep}(\beta 5)]\\ & \;+\varepsilon_{ij}(\mathrm{residuals}\;\mathrm{for}\;\mathrm{subject}\;i\;\mathrm{at}\;\mathrm{time}\;j)\\ & \;+U1_{i}(\mathrm{intercept}\;\mathrm{for}\;\mathrm{subject}\;i)\\ & \;+U2_{i}(\mathrm{slope}\;\mathrm{for}\;\mathrm{subject}\;i\;\mathrm{in}\;\mathrm{relation}\;\mathrm{to}\;\mathrm{Time}) \end{array}$$

where fixed effects include: Time (time of day normalized for chronotype using the ‘time distance’ between the *T*_Br_ measurement and MSF_sc_ for that participant as a proportion of a linearized unit circle where 0 = MSF_sc_ and 1 = 24 h); Sex (participant biological sex categorized as male, luteal female, or non-luteal female); BrainRegion (brain voxel categorized to one of six regions: Superficial 1, Superficial 2, Superficial 3, Superficial 4, Thalamus, Hypothalamus); Age (participant age on date of temperature measurement); and Sleep (whether participant reported falling asleep during scanning; categorized as ‘yes’, ‘maybe’ or ‘no’) with random effects for intercept by subject and for slope by subject with respect to time.

To confirm that there was no relationship between *T*_Br_ and BMI, the model was run a second time, but replacing the Sleep effect with BMI. The model for deep *T*_Br_ was identical to the BrainTemp model above except that only thalamic and hypothalamic regions were included (Supplementary material).

For the final analysis of patient data, a generalized linear mixed model for logit binomial distribution of patient outcome was chosen (the rationale for model choice is provided in Supplementary material). Survival in intensive care or ‘alive’ was specified as a miss and death or ‘dead’ as a hit. The model incorporated fixed effects and random effect for intercept and was built as follows (‘daily rhythm’ results were included for *T*_Br_ only):

(4) $$\;\begin{array}{rl} \mathrm{Outcom}e_{i} & =[\mathrm{intercept}(\beta 0)+\mathrm{Age}(\beta 1)+\mathrm{Sex}(\beta 2)+\mathrm{BrainMean}(\beta 3)\\ & \;+\mathrm{BrainRange}(\beta 4)+\mathrm{Daily}(\beta 5)]\\ & \;+\varepsilon_{ij}(\mathrm{residuals}\;\mathrm{for}\;\mathrm{patient}\;i)\\ & \;+U1_{i}(\mathrm{intercept}\;\mathrm{for}\;\mathrm{patient}\;i) \end{array}$$

where fixed effects include: Age (patient age in intensive care); Sex (patient biological sex categorized as male or female); BrainMean (absolute mean *T*_Br_ throughout analysis window); BrainRange (*T*_Br_ range across analysis window); Daily (presence or absence of a daily *T*_Br_ rhythm within analysis window—categorized as ‘yes’ or ‘no’; see above for details on how tests for daily rhythmicity were performed), with random effects for intercept by subject.

The choice of fixed effects (predictors) to include in the model was based on our core study objectives, avoiding redundant terms and optimizing the model fit (Supplementary material). Missing data values for any of the model components were input as ‘NA’ and thus patients with values missing for one or more of the components were excluded from the model output. The most conservative approach was taken i.e., multiple imputation was not performed since the random nature of missing data could not be assumed.

Statistical modelling and other circular analyses were performed using R version 3.6.3^36^ and the circular,^37^ cosinor,^38^ cosinor2,^39^ lme4,^40^ effects,^41^ afex,^42^ Matrix,^43^ Cairo,^44^ yarrr,^45^ and car^41^ packages. The full reproducible code is provided in Supplementary material or is available on request to the lead author. All other analyses were performed in GraphPad Prism version 8.2.

### Data availability

Individual patient data contained within the CENTER-TBI database are not publicly available but permissions for access can be requested at https://www.center-tbi.eu/data. We are committed to sharing all other anonymized individual participant and patient data that would support further research. All shareable items are available immediately upon publication and indefinitely, or ending 5 years following article publication, by reasonable request from the corresponding author(s). Shareable items will be available to anyone who wishes to access them and for any purpose. Code for statistical modelling is provided in Supplementary material or is available on request to the corresponding author(s).

## Results

### Brain temperature variation after traumatic brain injury

**Figure 1:**
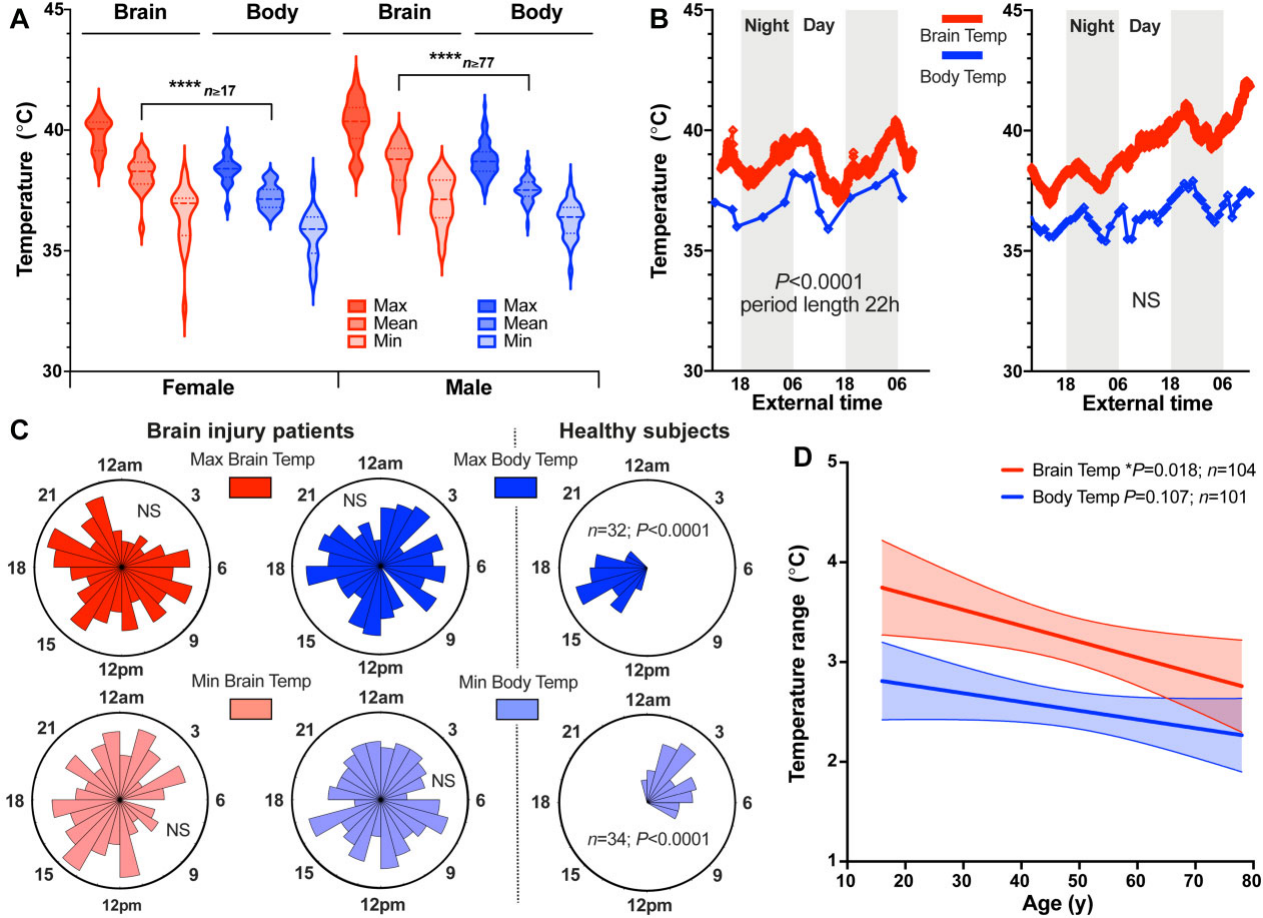
**Disrupted temperature rhythms in brain-injured patients.** (**A**) Violin plot of patient *T*_Br_ and *T*_Bo_ according to sex. Mean *T*_Br_ significantly greater than mean *T*_Bo_, mixed effects analysis with Tukey’s for multiple comparisons (****P < 0.0001, females, *n* = 20 for *T*_Br_ and *n* = 17 for *T*_Bo_; males, *n* = 85 for *T*_Br_ and *n* = 77 for *T*_Bo_). See also Supplementary Fig. 2. (**B**) Representative raw data from a 62-year-old female patient (*left*) showing daily variation in *T*_Br_ and *T*_Bo_, with *T*_Br_ consistently higher than *T*_Bo_ and both parameters in same phase. *T*_Br_ sampled once per minute; peak at 05:28 and nadir at 16:12 highlighting inversion of phase relationship with external day-night cycle under intensive care conditions (external time in 24-h clock format). Representative raw data from a 42-year-old male patient (*right*) showing lack of a daily rhythm in both *T*_Br_ and *T*_Bo_. See also Supplementary Fig. 3. (**C**) Rose plots (*left*) showing timings of temperature maxima and minima in TBI patients (24-h clock format). For all variables, the null hypothesis of a uniform distribution could not be rejected (Rayleigh test of uniformity; *T*_Br_Max, *n* = 104, *P* = 0.20; *T*_Br_Min, *n* = 104, *P* = 0.16; *T*_Bo_Max, *n* = 101, *P* = 0.99; *T*_Bo_Min, *n* = 101, *P* = 0.86). Contrast with healthy subject rectal temperature data from publicly-available database (*right*).^47^ (**D**) Linear regression of patient temperature ranges with age; reduction in temperature range significant for brain (slope of −0.016 significantly different from zero; 95% CI −0.029 to 0.003). Shaded areas represent 95% CIs for lines of best fit. Max = maximum; Min = minimum; NS = not significant. See also Supplementary Fig. 4.

Of 134 eligible patient records screened, 114 had at least 24 h of temperature data recorded (criteria level A). Of these, 110 patients had sufficient temperature data (≥36 h) for daily rhythm analysis (criteria levels A and B; for 10 of these patients, sufficient data were available for *T*_Bo_ only). Outcome in intensive care was available for 113/114 patients (criteria levels A and C), one or more injury severity scores (presence of a pupillary light reflex, Glasgow Coma Scale and/or Glasgow Coma Scale motor response) were available for 109/114 patients (criteria levels A and D). A total of 105 patients met all criteria levels; summary data for patients meeting key criteria levels are shown in Table 1. Mean *T*_Br_ (38.5 ± 0.8°C) was significantly higher than mean *T*_Bo_ (37.5 ± 0.5°C; *P* < 0.0001, Fig. 1A), with a range of 32.6 to 42.3°C. *T*_Br_ was not affected by the site of intracranial probe placement relative to focal injury (Supplementary Fig. 2). We found an approximately daily temperature rhythm in 27/110 patients; 25 of these patients had a daily rhythm in *T*_Br_ and 11 had a daily rhythm in *T*_Bo_ (with nine having a daily rhythm in both temperatures; Fig. 1B and Supplementary Fig. 3). However, across the cohort, the timings of temperature maxima and minima were poorly aligned with the external day-night cycle. This uncoupling of internal timing from the external solar cycle is typical of temperature rhythms when external timing cues are diminished;^46^ it lies in stark contrast to rectal temperature data from healthy individuals maintained under similar conditions but with daily light/dark and feed/fast cues that normally function to synchronize the body’s circadian rhythms with external environmental cycles (Fig. 1C). Notably, there was a relationship between *T*_Br_ and age; *T*_Br_ range was reduced in older patients (*P* = 0.018), driven predominantly by an upward trend in minimum *T*_Br_ (Fig. 1D and Supplementary Fig. 4).

**Table 1:** TBI patient demographics and summary temperature data

	Females (A)	Females (D)	Males (A)	Males (D)
Age (years)	55.0 (14.8) *n* = 21	54.3 (14.7) *n* = 20	45.8 (17.3) *n* = 93	46.2 (17.1) *n* = 85
*T*_Br_ Mean (°C)	38.2 (0.74) *n* = 20	38.1 (0.75) *n* = 19	38.6 (0.85) *n* = 85	38.6 (0.80) *n* = 79
*T*_Bo_ Mean (°C)	37.2 (0.51) *n* = 17	37.2 (0.52) *n* = 16	37.5 (0.51) *n* = 77	37.5 (0.49) *n* = 73
*T*_Br_ Range (°C)	3.38 (1.68) *n* = 20	3.46 (1.68) *n* = 19	3.21 (1.08) *n* = 84	3.25 (1.09) *n* = 78
*T*_Bo_ Range (°C)	2.65 (1.23) *n* = 17	2.75 (1.20) *n* = 16	2.50 (0.87) *n* = 84	2.52 (0.89) *n* = 76
*T*_Br_ Maximum (°C)	39.8 (0.74) *n* = 20	39.9 (0.73) *n* = 19	40.3 (1.03) *n* = 84	40.3 (0.97) *n* = 78
*T*_Bo_ Maximum (°C)	38.4 (0.63) *n* = 17	38.5 (0.50) *n* = 16	38.8 (0.70) *n* = 84	38.8 (0.69) *n* = 76
*T*_Br_ Minimum (°C)	36.4 (1.27) *n* = 20	36.4 (1.30) *n* = 19	37.1 (0.99) *n* = 84	37.1 (0.98) *n* = 78
*T*_Bo_ Minimum (°C)	35.7 (0.99) *n* = 17	35.7 (1.02) *n* = 16	36.3 (0.76) *n* = 84	36.3 (0.71) *n* = 76
PLR	1.86 (0.36) *n* = 21	1.85 (0.37) *n* = 20	1.79 (0.55) *n* = 87	1.79 (0.56) *n* = 84
GCS	7.90 (3.42) *n* = 21	7.90 (3.51) *n* = 20	8.09 (3.58) *n* = 88	8.07 (3.64) *n* = 85
GCSM	4.10 (1.61) *n* = 21	4.05 (1.64) *n* = 20	3.73 (1.85) *n* = 88	3.68 (1.87) *n* = 85

Data presented as mean (SD). GCS = Glasgow Coma Scale score; GCSM = Glasgow Coma Scale motor response score; PLR = presence of a pupillary light reflex in one or both eyes. Individual patient temperature values and ranges were calculated from all available data present within the data analysis window that met inclusion/exclusion criteria level A (Box 1). Aggregate results for males and females are presented for *n* = 114 patients meeting criteria level A (A) and again for *n* = 105 patients meeting criteria level D (D).

### Chronotype-controlled spatiotemporal measurements of human brain temperature

**Figure 2:**
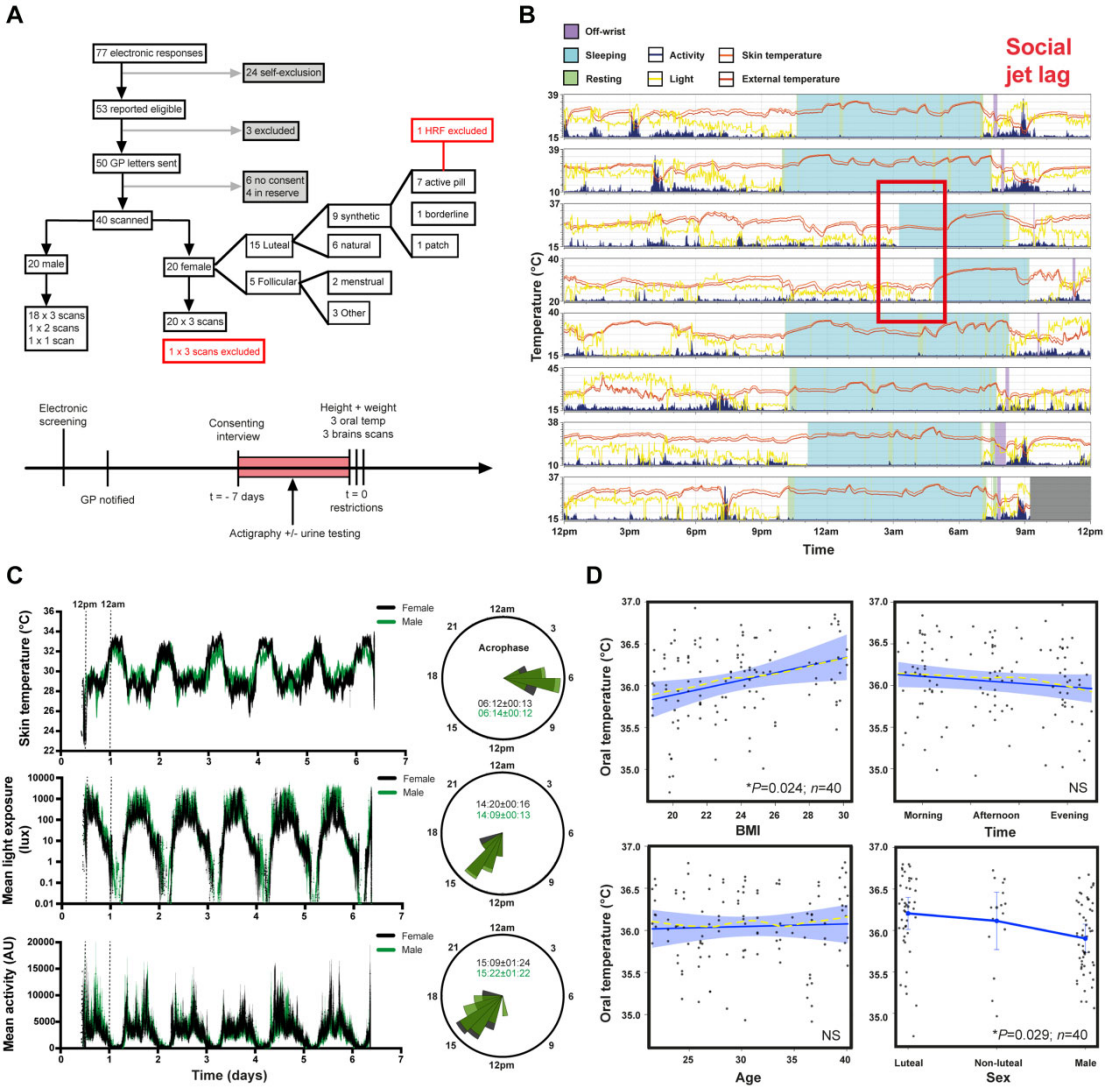
**Chronotype-controlled temperature variations in healthy adults.** (**A**) Prospective study profile and workflow (see the ‘Materials and methods’ section). (**B**) Representative actogram displaying typical actigraphy over 1 week from one male volunteer. Horizontal panels represent consecutive days. Note absence of light exposure and activity, with increase in skin temperature during sleep time (activity also absent when device was ‘off-wrist’). Social jet lag refers to large delay in sleeping schedule due to social activities on two consecutive days, highlighted (red box). See also Supplementary Fig. 5. (**C**) Group-averaged mean ± SEM data for distal skin (wrist) temperature, total light exposure and activity by sex during actigraphy week (*left*). Females *n* = 20, males *n* = 20. Associated rose plots with circular means (acrophases ± SD) displayed (*right*). For each data type, radial uniformity was rejected for both groups (Rayleigh uniformity test *P* < 0.0001) and there were no significant differences in circular mean between them (Watson’s two-sample test for homogeneity, *P* > 0.1). See also Supplementary Fig. 6. (**D**) Linear mixed modelling results for oral temperature. Solid blue lines represent model fits, shaded areas and double-ended error bars represent 95% CIs, dark grey circles display residuals (single temperature data-points) and smoothed dashed yellow lines represent partial residuals. The *x*-axis for time summarizes the continuous variable of time distance since the participant’s MSF_sc_ (proportion of a linearized unit circle, where 0 = MSF_sc_ and 1 = 24 h). Note time-dependent trend but lack of significant diurnal variation in oral temperature, likely reflecting inherent practical challenges of obtaining accurate oral temperature readings in human subjects.^48^ See also Supplementary Fig. 7.

Whilst the patient data were consistent with our hypothesis of *T*_Br_ rhythm disruption in the context of brain injury, this was based on an assumption that human *T*_Br_ normally varies by time of day. We set out to validate this prediction using MRS. Of 77 volunteers screened for eligibility, we recruited 20 males and 20 females (aged 20–40 years) between July and September 2019 (Fig. 2A). Participants represented 15 nationalities across five continents and the last participant was scanned on 8 October 2019. One male attended only for morning scanning and another male volunteer missed afternoon scanning; available data from both of these participants were included in the analysis. Of the females scanned, 11 had natural menstrual cycles, eight were taking a combined contraceptive pill and seven of these took an ‘active’ pill on the day of scanning. The female subject on a ‘pill break’ reported day one of menstruation at their afternoon scan; their *T*_Br_ data were included in the luteal group. Of the females with natural cycles, six were confirmed luteal (urine test), two were in menstruation and three were in non-menstrual follicular phase at scanning. Five females thus formed a non-luteal group. One female wore a combined contraceptive patch on the scanning day (transiently removed during each scan); their *T*_Br_ data were included in the luteal group.

**Figure 3:**
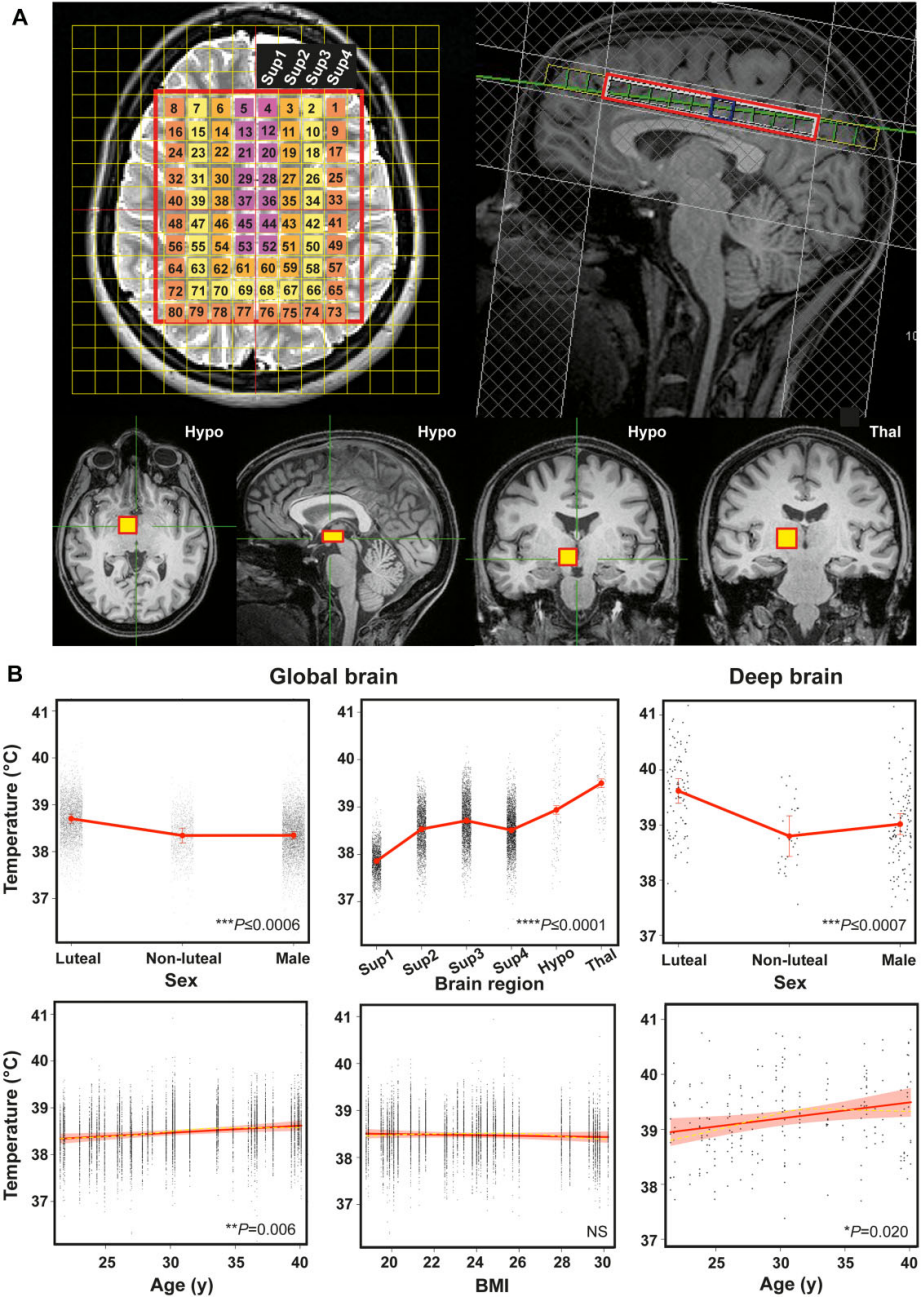
**Human brain temperature is spatially heterogenous.** (**A**) Representative annotated MR images to show MRS extraction protocol immediately after whole-brain structural acquisition. T_2_-weighted axial (*top left*) and T_1_-weighted mid-sagittal (*top right*) image showing multivoxel MRS overlay for more superficial brain regions including cerebral grey and white matter; note positioning superior to corpus callosum. From this multivoxel acquisition, MRS data were extracted from each of the numbered voxels individually; for the final statistical model, the whole cerebral region was split into four superficial groups of voxels (Sup 1–4, depicted as separate colours in the overlay, from medial to lateral). T_1_-weighted axial, sagittal and coronal images (bottom three images from left side, respectively) showing orthogonal positioning of single voxel in right hypothalamus (yellow box). T_1_-weighted coronal image (bottom right) showing positioning of single MRS voxel in right thalamus (yellow box). See also Supplementary Fig. 8. (**B**) Linear mixed modelling results for global *T*_Br_ by sex, age, brain region and BMI, and for deep *T*_Br_ (including thalamus and hypothalamus) by sex and age. Solid red lines represent model fits, shaded areas and double-ended error bars represent 95% CIs, dark grey circles display residuals (single temperature data-points) and smoothed dashed yellow lines represent partial residuals. For sex, *P*-value reflects comparisons of each group with luteal females. For brain region, *P*-value represents comparisons of each region relative to superficial region 1 (parasagittal group of voxels). Sup 1–4 = superficial brain regions 1–4 from medial to lateral; Hypo = hypothalamus; Thal = thalamus. See also Supplementary Fig. 9.

All subjects exhibited daily variation in wrist skin temperature, which was anti-phasic with their rhythm in activity and light exposure, in the week preceding their scans (Fig. 2B and C and Supplementary Figs 5 and 6). BMI was marginally higher in males (*P* = 0.014; Table 2). Oral temperature was 0.29°C higher in luteal females relative to males [95% confidence interval (CI) 0.03 to 0.58, *P* = 0.029] and 0.04°C higher for a unit increase in BMI (0.005 to 0.083, *P* = 0.024; Fig. 2D). There were no differences in oral temperature by age or time of day however, despite daily changes in environmental temperature (Fig. 2D and Supplementary Fig. 7). Brain locations for MRS data sampling are shown in Fig. 3A. MRS data from one female were excluded due to a health-related finding; 24 *T*_Br_ data-points from a total of 9434 (0.25%) were excluded because they did not meet quality control criteria for MRS spectral fitting (Supplementary material, Supplementary Fig. 8 and Supplementary Table 3). The data-points that failed quality control derived from 15 of the 40 subjects scanned. Together, these data confirmed that our cohort was representative of healthy adult males and females with respect to basic physiological parameters, chronotype distribution and sleep patterns. Furthermore, we had developed a novel chronotype-controlled imaging protocol that reproducibly obtains time-resolved *T*_Br_ data at high spatial resolution.

**Table 2:** Healthy participant demographics and sleep characteristics

	Females (*n* = 20)	Males (*n* = 20)
Age (years)	29.76 (5.48)	31.81 (6.16)
BMI	22.33 (2.80)	24.97 (3.65)^a^
No. days actigraphy data		
Free	1.6 (1.23)	1.65 (1.50)
Scheduled	6.0 (1.26)	5.95 (1.73)
Total	7.6 (0.60)	7.6 (0.94)
Sleep onset	23:33 (00:55)	23:59 (01:07)
Onset latency (min)	5.4 (4.26)	4.4 (2.33)
Sleep offset	07:40 (00:50)	07:50 (01:04)
Sleep duration (min)	486.5 (33.47)	474.2 (39.61)
Total sleep time per night (min)	442.4 (34.95)	424.8 (37.00)
WASO (min)	40.05 (17.90)	43.80 (22.07)
Sleep efficiency (%)	89.93 (3.86)	89.04 (4.99)
MSF_sc_	03:56 (01:01)	03:58 (01:26)
MSW_sc_	03:33 (00:50)	03:53 (01:01)
PCSM	03:15 (00:34)	03:31 (01:17)
SJL_sc_ (min)	52.27 (49.28)	38.82 (34.06)
Acrophase	15:09 (01:24)	15:22 (01:22)
Circadian function index	0.65 (0.07)	0.67 (0.08)
Oral temperature (°C)		
Morning	36.18 (0.51)	36.02 (0.40)
Afternoon	36.11 (0.60)	36.03 (0.48)
Evening	36.09 (0.57)	35.84 (0.43)
MRI room temperature (°C)		
Morning	21.02 (0.67)	21.36 (0.76)
Afternoon	21.98 (0.63)	21.94 (0.71)
Evening	21.30 (0.64)	21.38 (0.53)
Scan start time (clock time)^b^		
Morning	09:01 (00:02)	09:31 (00:02)
Afternoon	16:02 (00:05)	16:33 (00:11)
Evening	22:59 (00:02)	23:29 (00:02)
Time difference relative to MSF_sc_^c^		
Morning	05:17 (00.58)	05.46 (01.26)
Afternoon	12.14 (01.12)	12.29 (01.26)
Evening	19.12 (01.12)	19.41 (01.26)
Scan duration (min)		
Morning	31.80 (3.82)	31.10 (3.29)
Afternoon	30.50 (6.68)	29.61 (2.97)
Evening	29.55 (2.09)	28.63 (1.64)
Slept during scan		
Morning	1 (0)	2 (3)
Afternoon	6 (1)	6 (2)
Evening	5 (0)	5 (1)

Data presented as arithmetic mean (SD) except for sleep onset, sleep offset and acrophase [where circular mean (SD) is presented] and ‘slept during scan’, where numbers of individuals are presented as definite (possibly). Mean calculated across entire data collection period for each participant prior to calculation of group mean, where applicable. ‘Sleep onset’  is defined as bed time plus latency of sleep onset. ‘Sleep offset’ is wake up time. ‘Sleep duration’ is the duration between sleep onset and offset. ‘Total sleep time’ is the total duration of sleep period after removing periods of wakefulness. ‘Wake after sleep onset (WASO)’ refers to the summed duration of periods of wakefulness occurring after defined sleep onset; a reflection of sleep fragmentation. ‘Sleep efficiency’ is the percentage of time spent asleep while in bed, calculated by dividing the amount of time spent asleep by the total amount of time in bed. A normal sleep efficiency is considered to be 80% or higher. MSF_sc_ is calculated as the sleep onset on free days plus half of the average weekly sleep duration for all days. ‘Sleep-corrected midpoint of sleep on work days (MSW_sc_)’ is calculated as the sleep onset on work days plus half of the average weekly sleep duration for all days. ‘Previous corrected sleep midpoint (PCSM)’ is the sleep-corrected midpoint of sleep on the night before scanning. ‘Sleep-corrected social jetlag (SJL_sc_)’ is calculated as MSF_sc_ − MSW_sc_ or the absolute difference between sleep onset on free and work days when average sleep duration was longer on free than work days; if average sleep duration was longer on work days than free days, SJL_sc_ was calculated as the absolute difference between sleep offset on free and work days. Note that this parameter was calculated only for participants that reported at least one of each ‘day type’ (free or scheduled) during data collection. Circadian function index ranged from 0.43–0.73 in an age-matched group of healthy volunteers.^49^

^a^ BMI higher in males than females (*P* = 0.014; unpaired two-tailed *t*-test with Welch’s correction).

^b^ Scan time data from the female with a health-related finding have been excluded; there were only *n* = 18 males at the afternoon session and *n* = 19 males at the evening session.

^c^ Time difference relative to MSF_sc_ was converted to a proportion of a unit circle for each participant before incorporation into the linear mixed model in order to correct for chronotype.

### Human brain temperature varies by age, sex and brain region

Reflective of the patient data, healthy global *T*_Br_ (including all voxels measured) was higher than oral temperature (38.5 ± 0.4°C versus 36.0 ± 0.5°C); it was also 0.36°C higher in luteal females relative to follicular females and males (95% CI 0.17 to 0.55, *P* = 0.0006 and 0.23 to 0.49, *P* < 0.0001, respectively). This sex difference appeared to be driven by menstrual cycle phase (Supplementary Fig. 9). Despite age-selective recruitment, we captured an age-dependent increase in *T*_Br_, most notably in deep brain regions (thalamus and hypothalamus; 0.6°C over 20 years; 0.11 to 1.07; *P* = 0.0002). Sex, age and spatial effects on *T*_Br_ are summarized in Fig. 3B and Supplementary Fig. 10A. The *T*_Br_ range overall was 36.1 to 40.9°C, whilst the mean maximal spatial *T*_Br_ range (difference between hottest and coolest voxel in an individual at any given time point) was 2.41 ± 0.46°C. In the cerebrum, white matter-predominating areas were relatively warm. The lowest temperatures were observed in cortical grey matter regions lying close to the brain surface and adjacent to a major venous drainage channel (region Sup1, surrounding the superior sagittal sinus). The highest temperatures were observed in the thalamus (1.64°C higher than cortical grey matter, 1.57–1.72, *P* < 0.0001; 0.56°C higher than hypothalamus, 0.39–0.73, *P* < 0.0001). Eight female and 12 male participants reported having ‘definitely’ or ‘possibly’ fallen asleep during one or more scans; this had no measurable impact on *T*_Br_ within the 30-min scan time (Supplementary material). Collectively, these data show that normal human *T*_Br_ exceeds oral temperature and varies substantially by age, sex, menstrual cycle and brain region.

### Diurnal variation in human brain temperature

**Figure 4:**
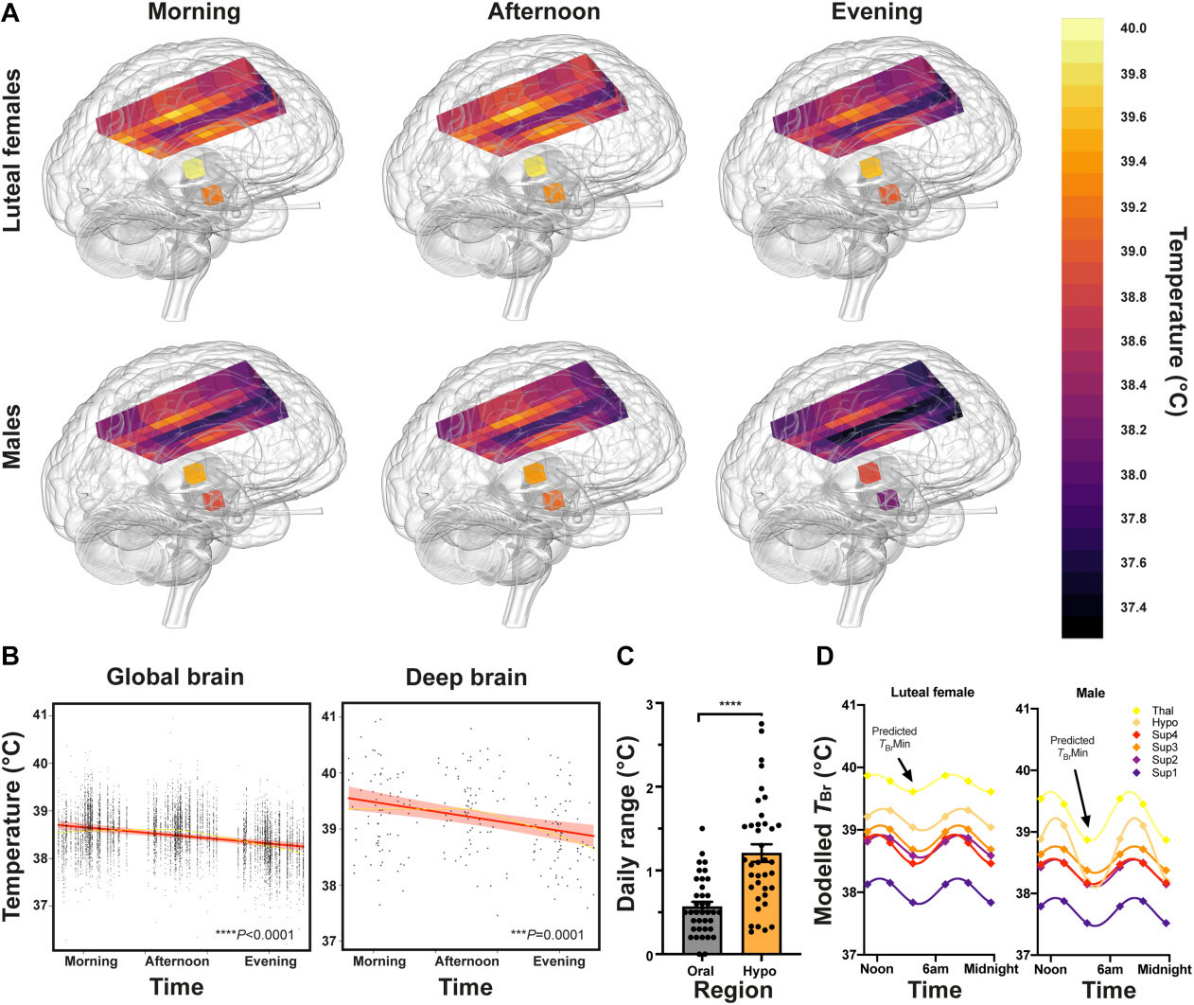
**Healthy brain temperature varies by time of day.** (**A**) Snapshot 3D maps of *T*_Br_ at each data collection point. Inferno colour scale is used to assign a temperature to each tissue voxel, to 0.1°C resolution. Aggregate temperatures are displayed in each voxel for luteal females (*n* = 14) and males (*n* = 20) separately. (**B**) Linear mixed modelling results for *T*_Br_ by time of day; results for global *T*_Br_ (*left*) and deep brain *T*_Br_ (thalamus and hypothalamus, *right*) are shown. Solid red lines represent model fits, shaded areas represent 95% CIs, dark grey circles display residuals (single temperature data-points) and smoothed dashed yellow lines represent partial residuals. The *x*-axis for time summarizes the continuous variable of time distance since the participant’s MSF_sc_ (proportion of a linearized unit circle, where 0 = MSF_sc_ and 1 = 24 h). (**C**) Temperature range (maximum versus minimum across three tested time points) for oral and hypothalamic sites for each healthy participant (*n* = 39). Temperature varied more by time of day in the hypothalamus than orally (repeated measures one-way ANOVA with Sidak’s multiple comparisons test ****P < 0.0001; see Supplementary Fig. 10B for other brain regions). (**D**) Schematic to model 24-h temperature rhythms of the healthy human brain. Extrapolated *T*_Br_ rhythms in healthy luteal females (*n* = 14) and males (*n* = 20), without controlling for age, BMI, or chronotype. Extrapolated temperature rhythms were created by duplicating the average temperatures measured at three time points and applying a 24 h sinusoidal fit to these six points. Note higher temperatures in all regions in luteal females relative to males and marked variation in deep brain temperatures in males. Arrows point to predicted *T*_Br_ minima around 2–3 am (approaching MSF_sc_). Sup1–4 = superficial brain regions 1–4 from medial to lateral; Hypo = hypothalamus; Thal = thalamus.

Absolute *T*_Br_ is ultimately determined by a balance between the rate of heat generated by the brain, and its rate of heat loss, mediated principally by CBF.^50,51^ Since blood arrives to the brain from the body at a lower temperature, this temperature gradient should enable effective brain heat removal, as long as cerebral perfusion is maintained.^52^ It follows that *T*_Br_ must be partially determined by *T*_Bo_. Since *T*_Bo_ and CBF both show clear diurnal regulation in humans, with lower temperature and higher CBF at night,^21,22^ we reasoned that human *T*_Br_ should drop in the evening. Our linear mixed model (Fig. 4A and B) revealed that global *T*_Br_ varied by 0.57°C (95% CI 0.40–0.75, *P* < 0.0001) across time; whereas deep brain locations varied by 0.86°C (0.37–1.26, *P* = 0.0001) and the hypothalamus displayed the greatest temporal variation (1.21 ± 0.65°C, range 0.27–2.75°C). Diurnal temperature variation was significantly greater in deep brain regions than in the cerebrum or the body (oral temperature; Fig. 4C and Supplementary Fig. 10B and C) and for all brain regions, *T*_Br_ was lowest at night.

Robust, approximately sinusoidal, daily *T*_Bo_ rhythms are a very well-characterized aspect of human physiology and similar temperature rhythms have been extensively documented in other diurnal mammals in the brain and body.^53,54^ Since *T*_Br_ is expected to depend (at least in part) on *T*_Bo_, we used the simplest and most appropriate mathematical model (24 h cosinor fit) to predict diurnal human *T*_Br_ in a continuous fashion. We extrapolated a sinusoidal time series for *T*_Br_ in six brain regions of interest (Fig. 4D). The predicted average minimum (anticipated around MSF_sc_, ∼3 am) was 38.4°C in luteal females and 38.0°C in males. Importantly, the diurnal range of measured *T*_Br_ across individuals in healthy cortical white matter—the location measured in patients with moderate-to-severe brain injury—was ∼37.0–40.3°C. In summary, these data reveal a remarkable sex- and brain region-dependent diurnal variation in normal human *T*_Br_.

### HEATWAVE: a 4D map of human brain temperature

Combining our spatial and temporal observations, we built HEATWAVE—a 4D map to model human *T*_Br_ at hourly resolution (Supplementary Videos 1 and 2). HEATWAVE can be dynamically explored at (https://www2.mrc-lmb.cam.ac.uk/groups/oneill/research/heatwave/). These comparisons highlight the relatively hot deep brain regions and their greater diurnal variation in males than females. The HEATWAVE videos complement the voxel maps in Fig. 4A, which represent a reference resource for interpreting human *T*_Br_ at each of the time points tested. Since each data-point in each map is an average of data from multiple individuals, it incorporates the range of ages, BMIs, and chronotypes expected for each sex in the demographic tested. Our data collection points also cater for the times (morning and afternoon) when most patients would present for MR-based neuroimaging in the non-acute setting. In addition to modelling diurnal human *T*_Br_ in a continuous fashion, HEATWAVE thus provides the first comprehensive spatially-resolved description of normal human *T*_Br_ at three clinically-relevant time points; a rich reference dataset for future studies in different age groups and patient cohorts.

### Daily brain temperature rhythms predict patient survival

**Figure 5:**
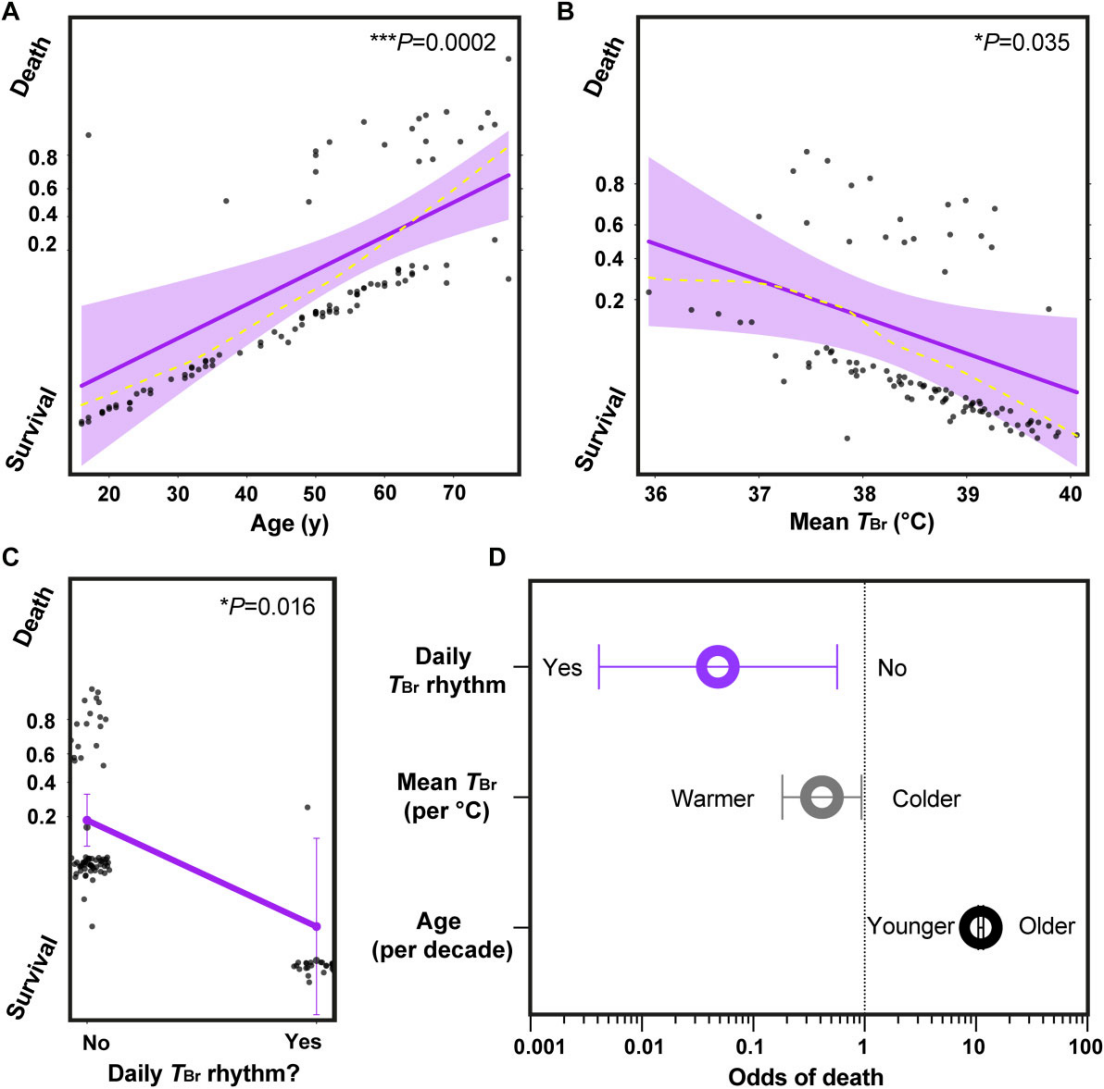
**A daily brain temperature rhythm predicts survival after brain injury.** (**A**–**C**) Generalized linear mixed model results for outcome in *n* = 98 TBI patients. Probability of death (‘success’ or ‘hit’ = 1) relative to survival (‘failure’ or ‘miss’ = 0) is depicted on the *y*-axis. Solid purple lines represent model fits for logit (log of the odds) binomial distribution for a given predictor and dark grey circles display residuals (individual patients). For numerical predictors (**A** and **B**), shaded areas represent 95% CIs and smoothed dashed yellow lines represent partial residuals. For the categorical predictor of presence/absence of a daily *T*_Br_ rhythm (**C**), residuals are jittered in the *x*-axis direction for visibility and 95% CIs are presented as double-ended error bars. (**D**) Odds of death in intensive care transformed from the data in **A**–**C**; the results for these three predictors are significant since the 95% CIs (double-ended error bars) do not include 1. Note also that CIs become numerically asymmetric once transformed from log odds to regular odds. Only factors that demonstrated a statistically significant relationship with mortality are shown. Note logarithmic scale on the *x*-axis and large effect size for presence of a daily rhythm in *T*_Br_ in (**D**). See the ‘Materials and methods’ section for further details on the generalized linear mixed model and Supplementary material for numerical outputs and related code.

The spatial, age- and sex-dependent outputs of HEATWAVE can guide interpretation of *T*_Br_ data in multiple clinical settings. The unique attraction of a 4D temperature map is readily envisaged for high temporal resolution data, however. With this in mind, we translated our core finding from HEATWAVE (that *T*_Br_ should normally vary in a time of day-dependent manner) back to our TBI patient dataset. Ninety-eight TBI patients had sufficient data to test for associations between *T*_Br_ variation and outcome (mortality) and 25 patients died in intensive care. Applying a generalized linear mixed model (Fig. 5), we found that lack of a daily *T*_Br_ rhythm, or an age increase of 10 years, increased the odds of death in intensive care 21-fold and 11-fold, respectively (odds ratio for death with rhythm 0.05; 95% CI 0.004–0.57, *P* = 0.016 and odds ratio for death with ageing by 1 year 1.10; 1.05–1.16, *P* = 0.0002). These relationships could not be explained by a general elevation in *T*_Br_, since mean *T*_Br_ was positively associated with survival (odds ratio for death 0.42 for 1°C increase, 0.18–0.94, *P* = 0.035). The presence of a daily *T*_Br_ rhythm did not correlate with either age or mean *T*_Br_ and critically, *T*_Br_ maxima and minima did not predict outcome (Supplementary material). Together, these data show that daily temperature variation is frequently disrupted or absent in TBI patients and that *T*_Br_ variation is of greater prognostic use than absolute *T*_Br_. Older TBI patients lacking a daily *T*_Br_ rhythm are at greatest risk of death in intensive care and presence of a daily *T*_Br_ rhythm appears to be one of the strongest single predictors of survival after TBI.^55^

## Discussion

We have established a 4D map of human *T*_Br_ and shown how this parameter varies with time of day, brain region, age, and sex in adults. Human brain tissue clearly functions normally at temperatures 1*–*3°C higher than generally assumed; this discovery alone has exigent implications for neurocritical care. These data provide clinicians with an urgently-needed and readily-accessible reference resource for evidence-based interpretation of *T*_Br_ data in patients. Finally, we have found that a daily *T*_Br_ rhythm is associated with a 21-fold increased chance of survival after brain injury, illustrating the high prognostic value of time-resolved *T*_Br_ measurements and empowering a temperature-based prediction of mortality.^23,56^ Overall these results indicate that daily *T*_Br_ rhythms are strongly associated with healthy brain function and become progressively compromised with age. Future studies should address whether supporting normal *T*_Br_ variation is beneficial to patients.

Essential to clinical diagnostics is the comparison of patient data with reference ranges from healthy individuals; MRS-thermometry now makes this possible for *T*_Br_. We have validated our core MRS findings using multiple complementary methods of temperature measurement. This is most pressing for *T*_Br_, where such methods are effectively mutually exclusive in healthy individuals and neurocritical care patients. TTM is the mainstay of neuroprotection subsequent to out-of-hospital cardiac arrest.^57^ Here, the objective is to reduce *T*_Br_, which is rarely measured directly in trials that test the therapeutic value of TTM in the context of brain injury. Cooling adults at the ‘wrong’ biological time or fixing patient temperatures at a constant target value may further compromise thermoregulation by abolishing physiologically-important, health-critical temperature variation. The highest temperature we observed in any healthy individual was 40.9°C in the thalamus of a luteal female in the afternoon; whilst the perception exists that a *T*_Br_ of this value would cause brain damage, there is no direct evidence for this, and similar deep brain temperatures are observed physiologically in other mammalian species.^58^ Furthermore, the *T*_Br_ range in our volunteers raises doubt over whether *T*_Br_ was abnormally high in some patient reports.^16^ Current temperature management guidelines do not consider physiological differences by sex or time of day,^59^ and whether adults should be cooled at all in neurocritical care is still debated. A clear understanding of how and why *T*_Br_ varies in health and disease is thus imperative. Here we report a healthy cortical white matter maximum *T*_Br_ of 40.3°C, but we caution strongly against overinterpreting single *T*_Br_ values or transitory trends. Rather, we recognize the need for technological solutions that allow individualized target temperature ranges to be determined, facilitating decision making that incorporates chronotype, age, sex, menstrual cycle and time of day.

Although time-based human neuroimaging studies are sparse, some morning/afternoon comparisons are consistent with diurnal regulation of brain morphometry,^60,61^ as well as diurnal variation in neural activity and metabolism.^62–64^ However, prior studies were underpowered without consideration of chronotype and a late evening time point, which provides greater insight into healthy brain physiology by incorporating the period approaching habitual sleep time (Fig. 4). Note that, in the absence of chronotype control (Supplementary Fig. 10), there was negligible difference in *T*_Br_ between morning and afternoon time points and the overall effect size for time-of-day variation is reduced compared to the output of the linear mixed model (Fig. 4). This underscores the value of our chronotype-controlled approach. It was neither practical nor clinically-relevant to deprive our participants of all external timing cues (to derive circadian *T*_Br_ variation), but this diurnal *T*_Br_ variation is almost identical to direct measurements obtained in healthy non-human primates under stringent conditions.^19^ A limitation of our prospective study design is that data could not be collected at the predicted *T*_Br_ nadir during sleep. Notwithstanding that patients are rarely scheduled for MRI scanning at 3 am, any attempt to collect MRS data at this time would have disrupted sleep and thus impaired our ability to obtain physiologically meaningful data. Though unlikely, it is conceivable that gross regional differences in the activity of cellular water impact upon the apparent spatial *T*_Br_ variation we observe within an individual at a given time point. However, we are unaware of any supporting evidence for this, nor can it be attributed to simple grey versus white matter distribution.^65^ Moreover, such differences cannot influence the *T*_Br_ variation we have found in relation to time of day, sex, age, or menstrual cycle stage. This is illustrated well when we limit our model to a subset of deep brain regions of more homogeneous tissue structure, where *T*_Br_ variation persists with respect to all of the aforementioned fixed effects (Figs 3B and 4B and Supplementary Fig. 7). Crucially, our robust statistical approach caters for multiple physiologically-relevant confounders within and between individuals that would have prevented the detection of significant *T*_Br_ variation in previous studies.^65,66^ Alongside the patient data (Fig. 5) and multiple parallel methods of temperature measurement in healthy subjects by us and others,^23,67–69^ our results offer compelling evidence of a daily temperature rhythm throughout the normal human brain (Supplementary material).

The within-brain temperature gradient is remarkable (Fig. 3B). As an ‘open’ thermodynamic system performing no mechanical work, aerobic metabolism of the brain releases heat at ∼0.66 J/min/g of tissue which is primarily removed by CBF.^52,70–72^ It is therefore highly likely that regional variation in neurovascular anatomy plays the chief role in creating spatial *T*_Br_ gradients (Supplementary material). Whilst not possible within the time constraints of our scanning protocol, arterial spin labelling could be used to confirm spatiotemporal relationships between *T*_Br_ and CBF—and indeed the morning-to-afternoon decrease in deep CBF shown previously using this method is consistent with the rising *T*_Br_ in our male volunteers.^73^ Although we cannot completely exclude a contribution from regional differences in water content,^65,74^ these are unlikely to explain the temperature difference between the thalamus and hypothalamus (both grey matter structures devoid of CSF). We suggest that the lower temperature of the hypothalamus might reflect its closer proximity to major vascular networks such as the Circle of Willis. In principle, technical limitations (Supplementary material) could potentially exaggerate MRS-derived temperature differences at the extreme edges of regions of interest (cerebral layer Sup4). The spatial distribution we have found is however very similar to non-human primates, excepting a larger gradient magnitude that is entirely consistent with the difference in brain volume between humans and rhesus monkeys.^20^ Unlike previous studies,^65,66^ we make no baseline assumption that temperature should be homogeneous across brain regions, nor between different tissue types within the brain. Importantly, we did not apply a post-acquisition correction to our MRS data to equalize temperatures between grey and white matter,^65,66^ since this would perpetuate the above assumption, and overlooks the clear tissue temperature differences observed in non-human primates and normothermic human patient brains.^15,18,20^ Indeed, higher temperatures in white matter-rich areas concur with predictions based on modelling perfusion, blood volume fraction and heat generation in different brain tissues.^14,75–77^

An increase in mean *T*_Br_ (Fig. 3B) and a trend upwards in minimum *T*_Br_ (Supplementary Fig. 4) with age suggests that overnight brain cooling becomes less efficient in older people, leading to a damped *T*_Br_ rhythm. This age-dependent reduction in *T*_Br_ range (and thus amplitude) is consistent with studies of *T*_Bo_ and may contribute to the disrupted sleep patterns and ‘sundowning’ symptoms of dementia patients.^28,78–80^ An age-dependent effect on *T*_Br_ both in healthy volunteers and TBI patients provides compelling evidence that age is an important factor to consider when interpreting *T*_Br_ data in humans and may play a role in brain thermoregulation. Cerebral blood supply is considered so efficient that heat removal is achieved without the need for other mechanisms under most circumstances,^50,51,81^ which seems intuitive for the young, healthy brain. However, the vast literature linking neurodegeneration to cerebrovascular compromise indicates that our key brain cooling mechanism progressively deteriorates with age (Supplementary material).^82,83^ Neuronal activity is highly sensitive to temperature change, with a Q_10_ of ∼2.3, although this is generally considered to be most problematic in the acute setting.^52,71,84^ In a study of 1130 epilepsy patients, 80–92% showed a 24-h cycle of seizure rates, with events most common at ∼8 am, when *T*_Br_ should increase most steeply (Fig. 4D).^85^ Given that cooling can terminate epileptic discharges,^86^ diurnal changes in *T*_Br_ may well contribute to diurnal variation in the incidence of seizures and cluster headache.^87,88^

*T*_Bo_ increases, and its overnight drop is blunted, in luteal versus follicular phase females.^68,89^ This menstrual variation predicts that *T*_Bo_ is ∼0.4°C higher in the early luteal phase.^89^ For the first time, we report a parallel luteal-phase increase in global *T*_Br_ by ∼0.36°C and of deep *T*_Br_ by 0.82°C (95% CI 0.37–1.28, *P* = 0.0006). This may contribute to the reported variable sleep patterns and changes in cognition across different stages of the menstrual cycle.^68,90^ A thermogenic effect of progesterone is well-recognized and may involve direct stimulation of preoptic/anterior hypothalamic thermoregulatory neurons or the suprachiasmatic nucleus.^68,69,91,92^ Despite its postulated neuroprotective effects, large clinical trials have failed to show any benefit of progesterone therapy for TBI—one reason for this could be damping of the daily *T*_Br_ rhythm (Fig. 4D).^93^ It is widely accepted that BMI positively correlates with *T*_Bo_ as found here (Fig. 2),^23,94^ and this may have impacted our ability to detect a time of day variation in oral temperature. Since BMI was slightly higher in males relative to females in our healthy cohort, a difference in BMI cannot explain the higher *T*_Br_ observed in luteal females and, notably, there was no relationship between BMI and *T*_Br_ overall (Fig. 3B and Supplementary material). This supports our conclusion that *T*_Br_ cannot be solely dependent on, nor predicted from, *T*_Bo_ since brain heat removal also occurs through routes that are unaffected by adipose deposition.^50,51^ Undoubtedly, our results emphasize the importance of including healthy controls of both sexes when exploring human brain physiology.

Prospective controlled trials are needed to confirm the predictive power of *T*_Br_ rhythmicity in relation to patient outcome, as well as the clinical utility of TTM protocols in brain-injured patients (Supplementary material). There may be traction in exploiting *T*_Br_ variation to detect or monitor focal pathologic processes such as neoplasia, trauma, vascular insults and epileptogenesis, but also more distributed inflammatory, metabolic and neuropsychiatric diseases.^70,95–101^ In particular, future work should be directed to address whether abnormal daily *T*_Br_ rhythmicity may serve as an early biomarker of neurodegeneration; a mechanistically opaque process for which early diagnostics are notably deficient.^30,82^ In this context, our findings offer an intriguing parallel to recent studies describing the effect of sleep deprivation on molecular clearance from the brain.^102^ Notably, brain regions with the highest absolute temperatures and greatest diurnal variation in HEATWAVE were those most affected by clearance failure in patients after sleep deprivation.^102^ We propose that disruption of daily *T*_Br_ variation induced acutely by sleep deprivation, and in the long term by ageing, might contribute to the build-up of protein aggregates in neurodegenerative disease. Ultimately, further *in vitro* and *in vivo* studies are needed to determine a mechanistic role for *T*_Br_ disturbance in acute and chronic brain disorders.

Whilst providing excellent spatial resolution, sequential MRS brain thermometry is clearly impractical for routine use in most clinical settings. Since core *T*_Bo_ is not a faithful proxy for *T*_Br_,^103^ our results highlight an urgent demand for cost-effective, non-invasive technologies that can capture longitudinal variations in *T*_Br_, alongside core body and peripheral temperatures. For now, the 2–3°C variation we have found between brain regions and by time of day can revolutionize human neural cellular platforms, advancing the physiological relevance of data obtained with these systems and our understanding of how the brain works.

## Supplementary Material

awab466_Supplementary_Data

## Appendix 1

### Investigators for The CENTER-TBI HR ICU Sub-Study

Full details are provided in the Supplementary material.

### Collaborators

Audny Anke, Ronny Beer, Bo-Michael Bellander, Erta Beqiri, Andras Buki, Manuel Cabeleira, Marco Carbonara, Giorgio Chevallard, Arturo Chieregato, Giuseppe Citerio, Endre Czeiter, Marek Czosnyka, Bart Depreitere, Ari Ercole, Shirin Frisvold, Raimund Helbok, Stefan Jankowski, Daniel Kondziella, Lars-Owe Koskinen, David K. Menon, Geert Meyfroidt, Kirsten Moeller, David Nelson, Anna Piippo-Karjalainen, Andreea Radoi, Arminas Ragauskas, Rahul Raj, Jonathan Rhodes, Saulius Rocka, Rolf Rossaint, Juan Sahuquillo, Oliver Sakowitz, Peter Smielewski, Ana Stevanovic, Nino Stocchetti, Nina Sundström, Riikka Takala, Tomas Tamosuitis, Olli Tenovuo, Peter Vajkoczy, Alessia Vargiolu, Rimantas Vilcinis, Stefan Wolf, Alexander Younsi, Frederick A. Zeiler.

## Abbreviations

BMI: body mass index
CBF: cerebral blood flow
CENTER-TBI =: Collaborative European NeuroTrauma Effectiveness Research in Traumatic Brain Injury
TBI: traumatic brain injury
MRS =: magnetic resonance spectroscopy
MSF_sc_: sleep-corrected midpoint of sleep on free days
*T*_Bo_: body temperature
*T*_Br_: brain temperature
TTM: targeted temperature management
