# Supplementary material for: A daily temperature rhythm in the human brain predicts survival after brain injury
Source: Brain. 2022 Jun 13;145(6):2031–48. doi: 10.1093/brain/awab466 (PMC9336587; doi:10.1093/brain/awab466)
Supplement: awab466_Supplementary_Data [file awab466_Supplementary_Data.zip › brain-2021-00914-File014.pdf]

SIEMENS MAGNETOM Prisma

\\Study Protocols\BRAIN\Other\CiBraT_E192051\t2_space_v4
TA: 3:42 PM: REF Voxel size: 0.9×0.9×0.9 mmPAT: 4 Rel. SNR: 1.00 : spcR

Properties

Prio recon	Off
Load images to viewer	On
Inline movie	Off
Auto store images	On
Load images to stamp segments	On
Load images to graphic segments	Off
Auto open inline display	Off
Auto close inline display	Off
Start measurement without further preparation	Off
Wait for user to start	Off
Start measurements	Single measurement

Routine

Slab group	1
Slabs	1
Position	Isocenter
Orientation	Transversal
Phase enc. dir.	R >> L
AutoAlign	Head > Brain
Phase oversampling	0 %
Slice oversampling	18.2 %
Slices per slab	176
FoV read	240 mm
FoV phase	100.0 %
Slice thickness	0.90 mm
TR	3200 ms
TE	408 ms
Averages	1.4
Concatenations	1
Filter	Raw filter, Distortion Corr.(2D), Prescan Normalize
Coil elements	HEA;HEP

Contrast - Common

TR	3200 ms
TE	408 ms
MTC	Off
Magn. preparation	None
Fat suppr.	None
Blood suppr.	Off
Restore magn.	On

Contrast - Dynamic

Averages	1.4
Reconstruction	Magnitude
Measurements	1
Multiple series	Each measurement

Resolution - Common

FoV read	240 mm
FoV phase	100.0 %
Slice thickness	0.90 mm
Base resolution	256
Phase resolution	100 %
Slice resolution	100 %
Phase partial Fourier	Allowed
Slice partial Fourier	Off
Interpolation	Off

Resolution - iPAT

PAT mode	GRAPPA
Accel. factor PE	2
Ref. lines PE	24
Accel. factor 3D	2
Ref. lines 3D	24
Reference scan mode	Integrated

Resolution - Filter Image

Image Filter	Off
Distortion Corr.	On
Mode	2D
Unfiltered images	On
Prescan Normalize	On
Unfiltered images	Off
Normalize	Off
B1 filter	Off

Resolution - Filter Rawdata

Raw filter	On
Elliptical filter	Off

Geometry - Common

Slab group	1
Slabs	1
Position	Isocenter
Orientation	Transversal
Phase enc. dir.	R >> L
Slice oversampling	18.2 %
Slices per slab	176
FoV read	240 mm
FoV phase	100.0 %
Slice thickness	0.90 mm
TR	3200 ms
Series	Interleaved
Concatenations	1

Geometry - AutoAlign

Slab group	1
Position	Isocenter
Orientation	Transversal
Phase enc. dir.	R >> L
AutoAlign	Head > Brain
Initial Position	Isocenter
L	0.0 mm
P	0.0 mm
H	0.0 mm
Initial Rotation	90.00 deg
Initial Orientation	Transversal

Geometry - Saturation

Fat suppr.	None
Restore magn.	On
Special sat.	None

Geometry - Navigator

Geometry - Tim Planning Suite

Set-n-Go Protocol	Off
Table position	H
Table position	0 mm

Geometry - Tim Planning Suite

Inline Composing	Off

System - Miscellaneous

Positioning mode	REF
Table position	H
Table position	0 mm
MSMA	S - C - T
Sagittal	R >> L
Coronal	A >> P
Transversal	F >> H
Coil Combine Mode	Adaptive Combine
Save uncombined	Off
Matrix Optimization	Performance
AutoAlign	Head > Brain
Coil Select Mode	On - AutoCoilSelect

System - Adjustments

B0 Shim mode	Tune up
B1 Shim mode	TrueForm
Adjust with body coil	Off
Confirm freq. adjustment	Off
Assume Dominant Fat	Off
Assume Silicone	Off
Adjustment Tolerance	Auto

System - Adjust Volume

Position	Isocenter
Orientation	Transversal
Rotation	0.00 deg
A >> P	263 mm
R >> L	350 mm
F >> H	350 mm
Reset	Off

System - pTx Volumes

B1 Shim mode	TrueForm
Excitation	Slab-sel.

System - Tx/Rx

Frequency 1H	123.244480 MHz
Correction factor	1
Gain	High
Img. Scale Cor.	1.000
Reset	Off
? Ref. amplitude 1H	0.000 V

Physio - Signal1

1st Signal/Mode	None
Trigger delay	0 ms
TR	3200 ms
Concatenations	1

Physio - Cardiac

Magn. preparation	None
Fat suppr.	None
Dark blood	Off
FoV read	240 mm
FoV phase	100.0 %
Phase resolution	100 %

Physio - PACE

Resp. control	Off
Concatenations	1

Inline - Common

Subtract	Off
Measurements	1
StdDev	Off
Save original images	On

Inline - MIP

MIP-Sag	Off
MIP-Cor	Off
MIP-Tra	Off
MIP-Time	Off
Save original images	On

Inline - Composing

Inline Composing	Off
Distortion Corr.	On
Mode	2D
Unfiltered images	On

Sequence - Part 1

Introduction	On
Dimension	3D
Elliptical scanning	Off
Reordering	Linear
Flow comp.	No
Echo spacing	3.61 ms
Adiabatic-mode	Off
Bandwidth	723 Hz/Px

Sequence - Part 2

Echo train duration	910 ms
RF pulse type	Normal
Gradient mode	Fast
Excitation	Slab-sel.
Flip angle mode	T2 var
Turbo factor	282

Sequence - Assistant

Allowed delay	30 s

\\Study Protocols\BRAIN\Other\CiBraT_E192051\t1_mprage_sag_p3_iso_Munich

TA: 3:45 PM: REF Voxel size: 1.0×1.0×1.0 mmPAT: 3 Rel. SNR: 1.00 : tfl

Properties

Prio recon	Off
Load images to viewer	On
Inline movie	Off
Auto store images	On
Load images to stamp segments	On
Load images to graphic segments	Off
Auto open inline display	Off
Auto close inline display	Off
Start measurement without further preparation	Off
Wait for user to start	Off
Start measurements	Single measurement

Routine

Slab group	1
Slabs	1
Dist. factor	50 %
Position	Isocenter
Orientation	Sagittal
Phase enc. dir.	A >> P
AutoAlign	Head > Basis
Phase oversampling	0 %
Slice oversampling	0.0 %
Slices per slab	192
FoV read	256 mm
FoV phase	100.0 %
Slice thickness	1.00 mm
TR	2500.0 ms
TE	4.37 ms
Averages	1
Concatenations	1
Filter	Distortion Corr.(2D), Prescan Normalize
Coil elements	HEA;HEP

Contrast - Common

TR	2500.0 ms
TE	4.37 ms
Magn. preparation	Non-sel. IR
TI	1100 ms
Flip angle	7 deg
Fat suppr.	Water excit. fast
Water suppr.	None

Contrast - Dynamic

Averages	1
Averaging mode	Long term
Reconstruction	Magnitude
Measurements	1
Multiple series	Each measurement

Resolution - Common

FoV read	256 mm
FoV phase	100.0 %
Slice thickness	1.00 mm
Base resolution	256
Phase resolution	100 %
Slice resolution	100 %
Phase partial Fourier	7/8
Slice partial Fourier	Off

Resolution - Common

Interpolation	Off

Resolution - iPAT

PAT mode	GRAPPA
Accel. factor PE	3
Ref. lines PE	24
Accel. factor 3D	1
Reference scan mode	Integrated

Resolution - Filter Image

Image Filter	Off
Distortion Corr.	On
Mode	2D
Unfiltered images	On
Prescan Normalize	On
Unfiltered images	Off
Normalize	Off
B1 filter	Off

Resolution - Filter Rawdata

Raw filter	Off
Elliptical filter	Off

Geometry - Common

Slab group	1
Slabs	1
Dist. factor	50 %
Position	Isocenter
Orientation	Sagittal
Phase enc. dir.	A >> P
Slice oversampling	0.0 %
Slices per slab	192
FoV read	256 mm
FoV phase	100.0 %
Slice thickness	1.00 mm
TR	2500.0 ms
Multi-slice mode	Single shot
Series	Interleaved
Concatenations	1

Geometry - AutoAlign

Slab group	1
Position	Isocenter
Orientation	Sagittal
Phase enc. dir.	A >> P
AutoAlign	Head > Basis
Initial Position	Isocenter
L	0.0 mm
P	0.0 mm
H	0.0 mm
Initial Rotation	0.00 deg
Initial Orientation	Sagittal

Geometry - Navigator

Geometry - Tim Planning Suite

Set-n-Go Protocol	Off
Table position	H
Table position	0 mm
Inline Composing	Off

System - Miscellaneous

Positioning mode	REF
Table position	H
Table position	0 mm
MSMA	S - C - T
Sagittal	R >> L
Coronal	A >> P
Transversal	F >> H
Coil Combine Mode	Adaptive Combine
Save uncombined	Off
Matrix Optimization	Off
AutoAlign	Head > Basis
Coil Select Mode	On - AutoCoilSelect

System - Adjustments

B0 Shim mode	Standard
B1 Shim mode	TrueForm
Adjust with body coil	Off
Confirm freq. adjustment	Off
Assume Dominant Fat	Off
Assume Silicone	Off
Adjustment Tolerance	Auto

System - Adjust Volume

Position	Isocenter
Orientation	Sagittal
Rotation	0.00 deg
A >> P	256 mm
F >> H	256 mm
R >> L	192 mm
Reset	Off

System - pTx Volumes

B1 Shim mode	TrueForm
Excitation	Non-sel.

System - Tx/Rx

Frequency 1H	123.244480 MHz
Correction factor	1
Gain	Low
Img. Scale Cor.	1.000
Reset	Off
? Ref. amplitude 1H	0.000 V

Physio - Signal1

1st Signal/Mode	None
TR	2500.0 ms
Concatenations	1

Physio - Cardiac

Magn. preparation	Non-sel. IR
TI	1100 ms
Fat suppr.	Water excit. fast
Dark blood	Off
FoV read	256 mm
FoV phase	100.0 %
Phase resolution	100 %

Physio - PACE

Resp. control	Off
Concatenations	1

Inline - Common

Subtract	Off
Measurements	1
StdDev	Off
Save original images	On

Inline - MIP

MIP-Sag	Off
MIP-Cor	Off
MIP-Tra	Off
MIP-Time	Off
Save original images	On

Inline - Composing

Inline Composing	Off
Distortion Corr.	On
Mode	2D
Unfiltered images	On

Inline - MapIt

Save original images	On
MapIt	None
Flip angle	7 deg
Measurements	1
TR	2500.0 ms
TE	4.37 ms

Sequence - Part 1

Introduction	Off
Dimension	3D
Elliptical scanning	Off
Reordering	Linear
Asymmetric echo	Off
Flow comp.	No
Multi-slice mode	Single shot
Echo spacing	11.1 ms
Bandwidth	140 Hz/Px

Sequence - Part 2

RF pulse type	Fast
Gradient mode	Fast
Excitation	Non-sel.
RF spoiling	On
Incr. Gradient spoiling	Off
Turbo factor	192

Sequence - Assistant

Mode	Off

\\Study Protocols\BRAIN\Other\CiBraT_E192051\csi_centrum_semiovale_TBr_144
TA: 4:52 PM: REF Voxel size: 10.0×10.0×10.0 mmRel. SNR: 1.00 : csislsr

Properties

Prio recon	Off
Load images to viewer	On
Inline movie	Off
Auto store images	On
Load images to stamp segments	Off
Load images to graphic segments	Off
Auto open inline display	Off
Auto close inline display	Off
Start measurement without further preparation	Off
Wait for user to start	Off
Start measurements	Single measurement

Routine

Position	L0.3 A12.2 H15.4 mm
Orientation	T > C-2.8 > S-2.4
Rotation	1 deg
Slices	1
Vol A >> P	100 mm
Vol R >> L	90 mm
FoV A >> P	160 mm
FoV R >> L	160 mm
Thickness F >> H	10 mm
TR	1200 ms
TE	144 ms
Averages	3
Filter	Prescan Normalize, Hamming
Coil elements	HEA;HEP

Contrast

TR	1200 ms
TE	144 ms
Averages	3
Averaging mode	Long term
Flip angle	65 deg
Water suppr.	Weak water suppr.
Water suppr. BW	50 Hz
Measurements	1

Resolution - Common

FoV R >> L	160 mm
FoV A >> P	160 mm
Thickness F >> H	10 mm
Scan res. R >> L	16
Scan res. A >> P	16
Interpol. res. R >> L	16
Interpol. res. A >> P	16
Hamming	On
Width	50
Prescan Normalize	On
Vector size	1024

Geometry - Common

Position	L0.3 A12.2 H15.4 mm
Orientation	T > C-2.8 > S-2.4
Rotation	1 deg
FoV R >> L	160 mm
FoV A >> P	160 mm
Thickness F >> H	10 mm

Geometry - Common

Vol R >> L	90 mm
Vol A >> P	100 mm
Sat. region	1
Thickness	40 mm
Position	R67.7 P0.2 H6.1 mm
Orientation	S > T5.2 > C0.2
Sat. delta frequ.	-3.40 ppm
Sat. region	2
Thickness	40 mm
Position	L67.1 A1.0 F2.4 mm
Orientation	S > T2.0 > C0.9
Sat. delta frequ.	-3.40 ppm
Sat. region	3
Thickness	40 mm
Position	L5.5 A7.2 F13.3 mm
Orientation	T > C-2.7 > S-2.5
Sat. delta frequ.	-3.40 ppm
Sat. region	4
Thickness	40 mm
Position	R1.6 A84.8 H4.3 mm
Orientation	C > T2.9 > S-1.0
Sat. delta frequ.	-3.40 ppm
Sat. region	5
Thickness	40 mm
Position	L0.9 P58.8 F3.2 mm
Orientation	C > T3.1 > S-0.9
Sat. delta frequ.	-3.40 ppm
Sat. region	6
Thickness	40 mm
Position	L7.9 A4.3 H42.4 mm
Orientation	T > C-3.0 > S-2.5
Sat. delta frequ.	-3.40 ppm

Geometry - AutoAlign

Slice group	1
Position	L0.3 A12.2 H15.4 mm
Orientation	T > C-2.8 > S-2.4
Phase enc. dir.	A >> P
AutoAlign	Head > Brain
Initial Position	L0.7 A0.5 H33.7
L	0.7 mm
A	0.5 mm
H	33.7 mm
Initial Rotation	91.75 deg
Initial Orientation	Transversal

System - Miscellaneous

Positioning mode	REF
Table position	F
Table position	23 mm
MSMA	S - C - T
Sagittal	R >> L
Coronal	A >> P
Transversal	F >> H
Save uncombined	Off
AutoAlign	Head > Brain
Coil Select Mode	Default

System - Adjustments

B0 Shim mode	Brain

System - Adjustments

B1 Shim mode	TrueForm
Adj. water suppr.	On
Adjust with body coil	Off
Confirm freq. adjustment	On
Only after freq. change	On
Assume Dominant Fat	Off
Assume Silicone	Off
Adjustment Tolerance	Auto

System - Adjust Volume

Position	L0.3 A12.2 H15.4 mm
Orientation	T > C-2.8 > S-2.4
Rotation	91.00 deg
R >> L	90 mm
A >> P	100 mm
F >> H	10 mm
Reset	Off

System - pTx Volumes

B1 Shim mode	TrueForm

System - Tx/Rx

Frequency 1H	123.244480 MHz
Gain	High
Img. Scale Cor.	1.000
Reset	Off
? Ref. amplitude 1H	0.000 V

Sequence - Common

Preparation scans	4
Dimension	2D
Delta frequency	-2.70 ppm
Phase encoding	Weighted
Bandwidth	2000 Hz
Acquisition duration	512 ms
Remove oversampling	Off

\\Study Protocols\BRAIN\Other\CiBraT_E192051\svs_hypothalamus_TBr_144
TA: 5:13 PM: REF Vol: 10 ×20 ×10 mmRel. SNR: 1.00 : svs_se

Properties

Prio recon	Off
Load images to viewer	On
Inline movie	Off
Auto store images	On
Load images to stamp segments	Off
Load images to graphic segments	Off
Auto open inline display	Off
Auto close inline display	Off
Start measurement without further preparation	Off
Wait for user to start	Off
Start measurements	Single measurement

Routine

Position	Isocenter
Orientation	Transversal
Rotation	0 deg
Vol R >> L	20 mm
Vol R >> L	20 mm
Vol F >> H	10 mm
TR	1200 ms
TE	144 ms
Averages	256
Filter	Prescan Normalize
Coil elements	HE1-4

Contrast

TR	1200 ms
TE	144 ms
Averages	256
Flip angle	65 deg
Water suppr.	Weak water suppr.
Water suppr. BW	50 Hz
Spectral suppr.	None
Measurements	1

Resolution - Common

Prescan Normalize	On
Vector size	1024

Geometry - Common

Position	Isocenter
Orientation	Transversal
Rotation	0 deg
Vol R >> L	20 mm
Vol A >> P	10 mm
Vol F >> H	10 mm

Geometry - AutoAlign

AutoAlign	---
Initial Position	Isocenter
L	0 mm
P	0 mm
H	0 mm
Initial Rotation	0.00 deg
Initial Orientation	Transversal

Geometry - Navigator

System - Miscellaneous

Positioning mode	REF
Table position	H
Table position	0 mm
MSMA	S - C - T
Sagittal	R >> L
Coronal	A >> P
Transversal	F >> H
Save uncombined	Off
Save single averages	Off
AutoAlign	---
Coil Select Mode	Default

System - Adjustments

B0 Shim mode	Brain
B1 Shim mode	TrueForm
Adj. water suppr.	On
Adjust with body coil	Off
Confirm freq. adjustment	On
Only after freq. change	On
Assume Dominant Fat	Off
Assume Silicone	Off
Adjustment Tolerance	Auto

System - Adjust Volume

Position	Isocenter
Orientation	Transversal
Rotation	0.00 deg
A >> P	10 mm
R >> L	20 mm
F >> H	10 mm
Reset	Off

System - pTx Volumes

B1 Shim mode	TrueForm

System - Tx/Rx

Frequency 1H	123.244480 MHz
Gain	High
Img. Scale Cor.	1.000
Reset	Off
? Ref. amplitude 1H	0.000 V

Physio - Signal1

1st Signal/Mode	None
TR	1200 ms

Physio - PACE

Resp. control	Off

Sequence - Common

Preparation scans	4
Delta frequency	-2.3 ppm
Ref. scan mode	Save all
No. of ref. scans	1
Phase cycling	Auto
Bandwidth	2000 Hz
Acquisition duration	512 ms
Remove oversampling	Off

\\Study Protocols\BRAIN\Other\CiBraT_E192051\svs_thalamus_TBr_144
TA: 2:40 PM: REF Vol: 15 ×15 ×15 mmRel. SNR: 1.00 : svs_se

Properties

Prio recon	Off
Load images to viewer	On
Inline movie	Off
Auto store images	On
Load images to stamp segments	Off
Load images to graphic segments	Off
Auto open inline display	Off
Auto close inline display	Off
Start measurement without further preparation	Off
Wait for user to start	Off
Start measurements	Single measurement

Routine

Position	Isocenter
Orientation	Transversal
Rotation	0 deg
Vol R >> L	15 mm
Vol R >> L	15 mm
Vol F >> H	15 mm
TR	1200 ms
TE	144 ms
Averages	128
Filter	Prescan Normalize
Coil elements	HE1-4

Contrast

TR	1200 ms
TE	144 ms
Averages	128
Flip angle	65 deg
Water suppr.	Weak water suppr.
Water suppr. BW	50 Hz
Spectral suppr.	None
Measurements	1

Resolution - Common

Prescan Normalize	On
Vector size	1024

Geometry - Common

Position	Isocenter
Orientation	Transversal
Rotation	0 deg
Vol R >> L	15 mm
Vol A >> P	15 mm
Vol F >> H	15 mm

Geometry - AutoAlign

AutoAlign	---
Initial Position	Isocenter
L	0 mm
P	0 mm
H	0 mm
Initial Rotation	0.00 deg
Initial Orientation	Transversal

Geometry - Navigator

System - Miscellaneous

Positioning mode	REF
Table position	H
Table position	0 mm
MSMA	S - C - T
Sagittal	R >> L
Coronal	A >> P
Transversal	F >> H
Save uncombined	Off
Save single averages	Off
AutoAlign	---
Coil Select Mode	Default

System - Adjustments

B0 Shim mode	Brain
B1 Shim mode	TrueForm
Adj. water suppr.	On
Adjust with body coil	Off
Confirm freq. adjustment	On
Only after freq. change	On
Assume Dominant Fat	Off
Assume Silicone	Off
Adjustment Tolerance	Auto

System - Adjust Volume

Position	Isocenter
Orientation	Transversal
Rotation	0.00 deg
A >> P	15 mm
R >> L	15 mm
F >> H	15 mm
Reset	Off

System - pTx Volumes

B1 Shim mode	TrueForm

System - Tx/Rx

Frequency 1H	123.244480 MHz
Gain	High
Img. Scale Cor.	1.000
Reset	Off
? Ref. amplitude 1H	0.000 V

Physio - Signal1

1st Signal/Mode	None
TR	1200 ms

Physio - PACE

Resp. control	Off

Sequence - Common

Preparation scans	4
Delta frequency	-2.3 ppm
Ref. scan mode	Save all
No. of ref. scans	1
Phase cycling	Auto
Bandwidth	2000 Hz
Acquisition duration	512 ms
Remove oversampling	Off
