# Supplementary material for: A daily temperature rhythm in the human brain predicts survival after brain injury
Source: Brain. 2022 Jun 13;145(6):2031–48. doi: 10.1093/brain/awab466 (PMC9336587; doi:10.1093/brain/awab466)
Supplement: awab466_Supplementary_Data [file awab466_Supplementary_Data.zip › brain-2021-00914-File015.pdf]

Supplementary Appendix 4

Academic and Clinical Central Office for Research and Development

**IRAS Number:**	244533	**REC Number:**	18-HV-045	**R&D Number:**	2019/0133
**Sponsor Number:**	AC 18038	**Site ID:**	E192051	**Study Acronym:**	CiBraT
**NIHR CPMS ID:**	42644	**Participant ID:**	CiBraT_	**Participant Initials:**	

Study Participant Data Form (Case Report Form)

Abbreviations: Y = yes, N = no, NA = not applicable, NR = not recruited, NK = not known, ND = not done, WD = withdrawn, RS = rescheduled, AE = adverse event, DC = declined

Study Details

Study title	Can we measure a diurnal shift in brain temperature in healthy human volunteers using Magnetic Resonance Spectroscopy (MRS)?		
Short study title	Circadian Brain Temperature (CiBraT) Study		
Chief Investigator	Dr Nina Rzechorzek (also Principal Investigator)		
Medical Statistician	Dr Francesca Chappell	Study Manager	Dr Duncan Martin
Local Collaborator	Prof Ian Marshall	Medical Physicist	Dr Michael J Thrippleton
Neuroradiologist	Dr Grant Mair	Location	Edinburgh Imaging (RIE) Facility
Study design	Prospective, single site, cohort study in healthy volunteers		
Jisc URL	https://mrc.onlinesurveys.ac.uk/cibrat		
I declare that this Section is complete and accurate to the best of my knowledge			
Chief Investigator	[signature] MRCVS	Date	DD __ MMM __ YYYY 2019

Participant Details

Initials		Sex	Male / Female
Age	YY / MM	Study ID	C i B r a T _
Postcode		Within 5 miles?	Y / N
Jisc response date	DD / MMM / YYYY	Urine kit needed?	Y / N / NA
I declare that this Section is complete and accurate to the best of my knowledge			
Chief Investigator	[signature] MRCVS	Date	DD __ MMM __ YYYY 2019

accord
Academic and Clinical Central Office for Research and Development
NHS Lothian

**IRAS Number:**	244533	**REC Number:**	18-HV-045	**R&D Number:**	2019/0133
**Sponsor Number:**	AC 18038	**Site ID:**	E192051	**Study Acronym:**	CiBraT
**NIHR CPMS ID:**	42644	**Participant ID:**	CiBraT_	**Participant Initials:**	

NHS Details

GP name			
GP address			
GP notified?	Y / N	GP letter date	DD MMM 2019 (YYYY)
GP concerns?	Y / N / NK	Cycle control: Pill / Patch / NA	Admin time: HH / NA
CHI number		Brand/NA	

I declare that this Section is complete and accurate to the best of my knowledge

Chief Investigator	[signature] MRCVS	Date	DD MMM 2019 (YYYY)

Consenting Interview (Pre-Scan Visit)

Date	DD MMM 2019 (YYYY)	Time	HH MM
Location			
Data entry	Dr Nina Rzechorzek	Willingness to participate rechecked?	Y / N / NR
Has participant read and understood PIS, CTPF, DPIS?			Y / N
Three copies of CTPF completed and signed?			Y / N / NR
Hard copies of PIS, signed CTPF, and DPIS given to participant?			Y / N / NR
Participant elected to be emailed about (a) study results and/or (b) future studies?			Y / N Y / N
MRI checklist completed?	Y / N	Checklist queries reported to radiography team?	Y / N / NA
Urine Test Kit supplied?	Y / N / NA / NR	Number of days to scan date	
ActTrust2 device issued		ActTrust2 participant ID	CiBraT_
Scans booked?	Y / N / NA	Travel expenses received?	Y / N / NA

I declare that this Section is complete and accurate to the best of my knowledge

Chief Investigator	[signature] MRCVS	Date	DD MMM 2019 (YYYY)

Academic and Clinical Central Office for Research and Development

IRAS Number:	244533	REC Number:	18-HV-045	R&D Number:	2019/0133
Sponsor Number:	AC 18038	Site ID:	E192051	Study Acronym:	CiBraT
NIHR CPMS ID:	42644	Participant ID:	CiBraT_	Participant Initials:	

Scan Visit 1 (Morning)

Date	DD / MMM / 2019 (YYYY)	Time	HH : MM		
Participant arrived by 8.45am (female) or 9.15am (male)?		Y / N / NA	Room temp.	.	
Location	Edinburgh Imaging (RIE) Facility (BRIC2)				
Data entry	Dr Nina Rzechorzek	Radiographer			
Participant well?	Y / N	Any AEs?	Y / N	AEs followed up?	Y / N / NA
Willingness checked?	Y / N / WD / RS	Actigraph removed?	Y / N		
Number of days Actigraph worn		Free / Scheduled		Patch removed?	Y / N / NA
MRI checklist signed?	Y / N	Ovulation confirmed?	Y / N / NA		
Oral temperature (°C)	.	Within range?	Y / N	33.2–38.1 °C women<br>35.7–37.7 °C men	
Height (m)	.	Weight (kg)	.		
BMI (kg/m²)	.	BMI within range (18.5-29.9)?	Y / N		
Prohibited medications or alcohol?	Y / N	Food or caffeine after 8am?	Y / N		
Vigorous exercise this morning?	Y / N	Eligibility confirmed?	Y / N		
Participant changed into hospital clothing?	Y / N	All MR sequences completed?	Y / N		
Reason for reschedule (if applicable, otherwise NA)					
Reason for withdrawal (if given, otherwise NA)					
Participant reported falling asleep during scan?	Y / N / NK	Scan duration (minutes)			
Meal vouchers (lunch) issued?	Y / N / DC	Visit duration (minutes)			
Actigraphy data transferred?	Y / N	ActTrust2 data erased?	Y / N		
I declare that this Section is complete and accurate to the best of my knowledge					
Chief Investigator	[signature] MRCVS	Date	DD / MMM / 2019 (YYYY)		

accord
Academic and Clinical Central Office for Research and Development
NHS Lothian

**IRAS Number:**	244533	**REC Number:**	18-HV-045	**R&D Number:**	2019/0133
**Sponsor Number:**	AC 18038	**Site ID:**	E192051	**Study Acronym:**	CiBraT
**NIHR CPMS ID:**	42644	**Participant ID:**	CiBraT_	**Participant Initials:**	

Scan Visit 2 (Afternoon)

Date	DD / MMM / 2019 (YYYY)	Time	HH : MM
Participant arrived by 3.45pm (female) or 4.15am (male)?	Y / N / NA	Room temp.	__ . _
Location	Edinburgh Imaging (RIE) Facility (BRIC2)		
Data entry	Dr Nina Rzechorzek	Radiographer	
Any AEs?	Y / N / NA	AEs followed up?	Y / N / NA
Participant reports feeling well?	Y / N	Willingness rechecked?	Y / N / WD / RS
MRI checklist reviewed and signed?	Y / N	Patch removed?	Y / N / NA
Oral temperature (°C)	__ . _	Within range?	Y / N — 33.2–38.1 °C women; 35.7–37.7 °C men
Prohibited medications or alcohol?	Y / N	Food or caffeine beyond 12-2pm?	Y / N
Vigorous exercise since this morning?	Y / N	Eligibility confirmed?	Y / N
Participant changed into hospital clothing?	Y / N	All MR sequences completed?	Y / N
Reason for withdrawal (if given, otherwise NA)			
Participant reported falling asleep during scan?	Y / N / NK	Scan duration (minutes)	
Meal vouchers (dinner) issued?	Y / N / DC	Visit duration (minutes)	

I declare that this Section is complete and accurate to the best of my knowledge

Chief Investigator	[signature] MRCVS	Date	DD / MMM / 2019 (YYYY)

Academic and Clinical Central Office for Research and Development

IRAS Number:	244533	REC Number:	18-HV-045	R&D Number:	2019/0133
Sponsor Number:	AC 18038	Site ID:	E192051	Study Acronym:	CiBraT
NIHR CPMS ID:	42644	Participant ID:	CiBraT_	Participant Initials:	

Scan Visit 3 (Evening)

Date	DD / MMM / 2019 (YYYY)	Time	HH / MM
Participant arrived by 10.45pm (female) or 11.15pm (male)?	Y / N / NA	Room temp.	.
Location	Edinburgh Imaging (RIE) Facility (BRIC2)		
Data entry	Dr Nina Rzechorzek	Radiographer	
Any AEs?	Y / N / NA	AEs followed up?	Y / N / NA
Participant reports feeling well?	Y / N	Willingness rechecked?	Y / N / WD / RS
MRI checklist reviewed and signed?	Y / N	Patch removed?	Y / N / NA
Oral temperature (°C)	.	Within range?	Y / N — 33.2–38.1 °C women, 35.7–37.7 °C men
Medications, caffeine or alcohol?	Y / N	Food beyond 6-8pm?	Y / N
Vigorous exercise since last scan?	Y / N	Eligibility confirmed?	Y / N
Participant changed into hospital clothing?	Y / N	All MR sequences completed?	Y / N
Reason for withdrawal (if given, otherwise NA)			
Participant reported falling asleep during scan?	Y / N / NK	Scan duration (minutes)	
Notes		Visit duration (minutes)	
Participant travel expenses received?	Y / N / NA	Expenses reimbursed?	Y / N / NA

I declare that this Section is complete and accurate to the best of my knowledge

Chief Investigator	[signature]	Date	DD / MMM / 2019 (YYYY)

accord
Academic and Clinical Central Office for Research and Development
NHS Lothian

**IRAS Number:**	244533	**REC Number:**	18-HV-045	**R&D Number:**	2019/0133
**Sponsor Number:**	AC 18038	**Site ID:**	E192051	**Study Acronym:**	CiBraT
**NIHR CPMS ID:**	42644	**Participant ID:**	CiBraT_	**Participant Initials:**	

Post-scan

Any AEs within 7 days?	Y / N / NA	AEs followed up?	Y / N / NA
Neuroradiology report completed?	Y / N	Any HRFs identified?	Y / N
HRF exclusion reported to CI?	Y / N / NA	HRFs reported to participant?	Y / N / NA
HRFs reported to GP?	Y / N / NA	Clinical radiology report sent to GP?	Y / N

I declare that this Section is complete and accurate to the best of my knowledge

Chief Investigator		Date	DD / MMM / YYYY 2019

Actigraphy chronotype data

Night	Type*	Sleep start	Sleep end	Sleep duration (min)	Sleep midpoint	MSF_{sc}/MSW_{sc}
1	F / S	HH MM	HH MM		HH MM	HH MM
2	F / S	HH MM	HH MM		HH MM	HH MM
3	F / S	HH MM	HH MM		HH MM	HH MM
4	F / S	HH MM	HH MM		HH MM	HH MM
5	F / S	HH MM	HH MM		HH MM	HH MM
6	F / S	HH MM	HH MM		HH MM	HH MM
7	F / S	HH MM	HH MM		HH MM	HH MM
8	F / S	HH MM	HH MM		HH MM	HH MM
Mean	F / S	HH MM	HH MM		Acrophase	HH MM
Mean MSF_{sc}		HH MM	Mean MSW_{sc}	HH MM	SJL_{sc}	HH MM

*F = free; S = scheduled (relating to wake time the following morning). MSF_{sc}/MSW_{sc} = sleep corrected midpoint of sleep on free/work days = sleep onset on free/work days plus half of the average weekly sleep duration (all days). SJL_{sc} = sleep corrected social jetlag (MSF_{sc}-MSW_{sc} = absolute difference between sleep onset on free and work days).

I declare that this Section is complete and accurate to the best of my knowledge

Chief Investigator		Date	DD / MMM / YYYY 2019

*refer to MRS database for blinded analysis and data points from each voxel

accord
Academic and Clinical Central Office for Research and Development
NHS Lothian

**IRAS Number:**	244533	**REC Number:**	18-HV-045	**R&D Number:**	2019/0133
**Sponsor Number:**	AC 18038	**Site ID:**	E192051	**Study Acronym:**	CiBraT
**NIHR CPMS ID:**	42644	**Participant ID:**	CiBraT_	**Participant Initials:**	

MRS data (post-unblinding)*

MRS data blinded by Study Manager?	Y / N		
Scan visit	v_1	v_2	v_3
MRS data quality			
Mean temperature superficial voxels °C	__ __ . __	__ __ . __	__ __ . __
Temperature thalamic voxel °C	__ __ . __	__ __ . __	__ __ . __
Temperature hypothalamic voxel °C	__ __ . __	__ __ . __	__ __ . __
Time to nearest acrophase	HH MM	HH MM	HH MM
Time since previous corrected sleep midpoint	HH MM	HH MM	HH MM
Time since average MSF_{sc}	HH MM	HH MM	HH MM

*refer to MRS database for blinded analysis and data points from each voxel

I declare that this Section is complete and accurate to the best of my knowledge			
Chief Investigator	[signature]	Date	DD MMM YYYY 2019

Deviations or Non-compliance with Study Protocol and Serious Breaches

Date	DD MMM YYYY	Time	HH MM
Nature of event			
Event requires exclusion?	Y / N		
Event impacts on data quality or integrity?	Y / N		
Event requires follow-up?	Y / N		
Sponsor notified?	Y / N / NA		
GP notified?	Y / N / NA		

I declare that this Section is complete and accurate to the best of my knowledge			
Chief Investigator	[signature]	Date	DD MMM YYYY 2019

accord
Academic and Clinical Central Office for Research and Development
NHS Lothian

IRAS Number:	244533	REC Number:	18-HV-045	R&D Number:	2019/0133
Sponsor Number:	AC 18038	Site ID:	E192051	Study Acronym:	CiBraT
NIHR CPMS ID:	42644	Participant ID:	CiBraT_	Participant Initials:	

Participant withdrawal

Date	DD MMM YYYY		
Elected withdrawal or CI-determined?			
Method of notification			
Primary reason for withdrawal (if available)			
GP notified of withdrawal by CI?			
I declare that this Section is complete and accurate to the best of my knowledge			
Chief Investigator	[signature]	Date	DD MMM YYYY 2019

Adverse event (AE) recording

Nature of event			
Start date	DD MMM YYYY		
Stop date	DD MMM YYYY		
Time	HH MM NK		
Location			
Seriousness		Severity	
Expectedness (only if possibly related)		Relatedness	
Outcome			
CI/PI oversight			
MedDRA code			
I declare that this Section is complete and accurate to the best of my knowledge			
Chief Investigator	[signature]	Date	DD MMM YYYY 2019

I declare that this Source Data Document is complete and accurate to the best of my knowledge			
Chief Investigator Final Sign Off	[signature]	Date	DD MMM YYYY 2019
