# Supplementary material for: A daily temperature rhythm in the human brain predicts survival after brain injury
Source: Brain. 2022 Jun 13;145(6):2031–48. doi: 10.1093/brain/awab466 (PMC9336587; doi:10.1093/brain/awab466)
Supplement: awab466_Supplementary_Data [file awab466_Supplementary_Data.zip › brain-2021-00914-File017.pdf]

Supplementary Material for

A daily temperature rhythm in the human brain predicts survival after brain injury

Nina M. Rzechorzek, Michael J. Thrippleton, Francesca M. Chappell, Grant Mair, Ari Ercole, Manuel Cabeleira, The CENTER-TBI High Resolution ICU (HR ICU) Sub-Study Participants and Investigators, Jonathan Rhodes, Ian Marshall, John S. O'Neill

Contents

Supplementary Methods
Supplementary Text
Supplementary Figures 1 to 10
Supplementary Box 1
Supplementary Tables 1 to 3
Supplementary Extended Data
Captions for Supplementary Videos 1 and 2
Supplementary References
Supplementary Appendices 1 to 5
STROBE Checklist

Other Supplementary material for this manuscript includes:

Supplementary Videos 1 and 2 (also located at https://www2.mrc-lmb.cam.ac.uk/groups/oneill/research/heatwave/).

Supplementary Methods

Actigraphy and chronotyping

Chronotype varies between sexes in an age-dependent manner,[1] as well as between individuals of the same sex. Actigraphy is a validated, non-invasive, and objective tool for studying sleep and circadian patterns.[2-5] We chose the ActTrust2 Wrist Actimeter (Condor Instruments, Sao Paulo, Brazil), sampling the following parameters at a frequency of 60 seconds: external temperature, skin temperature, rest/activity/sleep patterns, and various wavelengths of light (infrared, red, green, blue, UVA and UVB). All scanned participants wore their Actimeter for a minimum of 5 days and nights; they were asked to press an event button on the device once just before trying to sleep each night and again when they first awoke each morning. These reported sleep and wake times were validated against the actigraphy data and confirmed verbally with each participant on their scanning day. Data was transferred using the ActTrust Dock, then processed and analysed using ActTrust Studio software and an in-built Condor Instruments algorithm which uses Proportional Integral Mode (PIM) and Zero Crossing Mode normalized per second (ZCMn) data[6]. The light/dark phase was programmed to reflect the latitude and longitude (time zone with Coordinated Universal Time (UTC) offset of +1h) in Edinburgh, United Kingdom. Devices were retrieved, and the data downloaded and analysed, on the day of scanning; participants were asked to report which (if any) main sleep periods in the preceding week ended naturally ('free') or with an alarm or other disturbance ('scheduled'). Free and scheduled assignments were validated against the data. Sleep scoring parameters were enabled to include one or more main periods of sleep at night. Periods of 'off-wrist' time were excluded from the analysis. Sleep scoring was performed using the Cole-Kripke method (ActStudio software) to extract seven key sleep parameters for each night of sleep.[6] ActTrust2 data underwent further processing to derive eleven diurnal characteristics, as well as MSF_{sc} and sleep-corrected social jetlag (SJL_{sc}).[7-9]

Scanning protocol

The scanning protocol was adapted from Thrippleton et al.[10] Localizers and structural sequences were acquired prior to MRS to plan voxel placement. Structural acquisition included whole-brain axial T2-weighted (T2w; three-dimensional fast spin-echo; TR/TE = 3200/408 ms; field of view, 240 x 240 mm^2; 18.2% slice oversampling; phase-encoding direction R>>L; 0.9-mm isotropic resolution) and sagittal T1-weighted (T1w; three-dimensional inversion recovery-prepared gradient echo; TR/TI/TE = 2500/1100/4.37 ms; flip angle, 7°; field of view, 256 x 256 mm^2; 0% slice oversampling; phase-encoding direction A>>P; 1-mm isotropic resolution) sequences. Structural imaging was repeated at each scanning session and the last-acquired structural images were used for HRF screening. Magnetic resonance spectroscopic imaging (MRSI; semi-LASER sequence, TR/TE = 1200/144ms; flip angle, 65°)[11] of the cerebrum was acquired from a 10-mm thick axial slice located at the level of the centrum semiovale, generating multiple 10 × 10 × 10 mm voxels (Fig. 3A). Six saturation bands of 40mm thickness were applied to suppress scalp lipid and other signals on all faces of the volume of interest. Automated shimming and partial water suppression (50Hz bandwidth) were applied. For each phase-encoding step, a 512-ms free induction decay (FID) was obtained. Since a single-voxel semi-LASER MRS sequence was not available at the time of the study, single-voxel PRESS MRS acquisition (TR/TE = 1200/144 ms; flip angle, 65°; 50Hz bandwidth water suppression) was used to sample the thalamus (voxel size 15 × 15 × 15 mm) and hypothalamus (voxel size 20 × 10 × 10 mm) (Fig. 3A). Single voxels were positioned to avoid interference from cerebrospinal fluid and/or large blood vessels; spectral

quality was visually assessed in real-time to ensure that the water and NAA peaks were of a sufficient signal-to-noise ratio and the acquisition was repeated if necessary. Voxel position and orientation was matched between scanning sessions for each participant by a single radiographer who acquired all data for that individual at each time point. To maximise consistency of voxel positioning between participants, an illustrated placement protocol, specified by a senior neuroradiologist, was used. Foam pads were wedged between the skull and the head coil to eliminate any movement during acquisition. The whole procedure was optimized through pilot scanning, achieving a total scan time of ~30min, including shimming. All structural brain images were reviewed by a consultant neuroradiologist (G.M.). Further details on the scanning protocol are in Supplementary Appendix 3.

MRS data processing

MRS data were processed as described previously.[10] Briefly, free induction decays were apodised (Gaussian function, 4 Hz full width at half height) and the NAA and water chemical shifts determined by time-domain fitting of both resonances. For each voxel, four quality control filters were applied: a cut off for NAA linewidth (13 Hz for multivoxel data; 15.5 Hz for single voxel data), an R-squared measure of the NAA fit quality (0.80), a cut off for H_2O linewidth (13 Hz for multivoxel data; 15.5 Hz for single voxel data), and an R-squared measure of H_2O fit quality (0.93). Whilst the consensus recommendation threshold for H_2O linewidth is 0.1 ppm = 12 Hz,[12] the linewidth cut-offs used here included the effect of the Gaussian apodization applied. Hypothalamus and thalamus are challenging to shim, and the single voxels were larger than those acquired with the multivoxel sequence. For these reasons, the single-voxel data was expected to be of lower quality with a slightly increased linewidth (reflected in the higher linewidth cut-off). Data from a voxel was discarded if it failed one or more of the set cut-offs and if the resultant data point affected the range of T_{Br} values for that participant at that time point, and/or the range for that voxel at that time point across a given sex group. The means and standard deviations for linewidths for NAA and H_2O are reported in Supplementary Table 3; spectral curve fitting and peak annotation are depicted in Supplementary Fig. 8. Spectral fitting was performed in batch mode without investigator intervention. Processing of MRS data was fully automated.

Rationale for prospective study sampling and linear mixed model

For our primary research question addressing whether there was a time of day variation in human T_{Br} using MRS, we had two main options: (1) sample a few individuals at high temporal resolution, or (2) sample many individuals at low temporal resolution. Given our secondary objectives (to determine T_{Br} variation by brain location and by sex) the second option was clearly preferable, since it offered sufficient power to detect such changes. Three time points were chosen for sampling, equally spaced across the extent of waking hours for an average human, and covering the range of times when patients would most likely be scheduled for advanced diagnostic imaging in the clinic. The null hypothesis was no change in T_{Br} by time of day; given the known, roughly sinusoidal daily variation in human T_{Bo}, a two time-point design carried a small but concerning risk of selecting two points at which T_{Br} returned to baseline. Therefore, three time points was considered the simplest design that would allow T_{Br} variation over time to be detected. Whilst time-dependent analyses exploring only the influence of time on a parameter classically employ nonlinear models, a linear mixed model approach was chosen for the following reasons (i) to accommodate fixed (e.g. time, age, sex, BMI) and random effects (allowing individual participants to have different baseline temperatures and different changes in temperature over time) (ii) to

accommodate data nested at multiple levels, not least that T_{Br} measurements would be correlated within individuals, (iii) to accommodate repeated measurements per participant, (iv) to enable the use of data from all participants provided they undergo at least one, but not necessarily all 3 scans, and (v) to enable the incorporation of chronotype-control (with the proviso that 'time' would have to be modelled as a continuous, rather than a categorical variable).

Data management

Source data for the prospective study included electronic screening questionnaire responses (hosted by Jisc online surveys, Bristol, UK), actigraphy data logs (electronic format) and Study Participant Data Forms (Supplementary Appendix 4; hard and electronic format, hosted by the Centre for Clinical Brain Sciences, University of Edinburgh), DICOM files and other raw imaging data (stored at Edinburgh Imaging, hosted by the University of Edinburgh). Clinical imaging reports used for HRF screening were hosted by NHS Lothian; no members of the Study research team had access to these reports apart from the designated Neuroradiologist (G.M.). Data derived from human participants was managed and shared according to terms described in the Consent to Participate Form (Supplementary Appendix 1) and Data Protection Information Sheet, in compliance with the revised GDPR (https://www.eugdpr.org).

Patient temperature data analyses

Temperature data from each patient were subjected to an analysis pipeline to determine the presence/absence of a daily rhythm in T_{Br} and/or T_{Bo}. Since different algorithms frequently produce quite different results from the same data, we aimed to remove any potential for subjective bias in the choice of analysis method, such that a positive result from any single method should not be sufficient to assign any individual dataset as having a 'daily rhythm'. After confirming that sufficient data were available for rhythmic analysis, the first criterion for assigning a dataset as having a 'daily rhythm' was that a rhythm of period length ~22-26h could be detected by visual analysis of the raw data in GraphPad Prism. This method was prioritised because it simulates what any clinician could practically achieve at the bedside by looking at the raw data without any assumed knowledge of chronobiology or applying algorithms to test for rhythmicity. However, this visual method alone was not considered sufficient, since human error may generate false positives as well as false negatives. Hence the data were then tested using a battery of validated algorithms that are well established in biological rhythms research. The second criterion was that the dataset had to have a 'daily rhythm' according to at least one of three semi-automated methods (cosinor analysis in GraphPad Prism, Harmonic Regression in R, or analysis in Biodare2). For cosinor analysis in Prism, a cosinor fit was tested against the null hypothesis of a straight line; the cosinor fit was modelled as a non-decaying cosine wave oscillating around a baseline that may change in a linear fashion over time:

$$y = baseline + (m * x) + amplitude * cos\,(((2 * \pi * (x - \phi))/\tau))$$

where:
x = time
y = patient temperature (brain or body)
m = linear trend function
$baseline$ = the y (temperature) value around which the wave oscillates

$amplitude$ = height of the top of wave
τ = period (the time it takes for a complete cycle, in units of x)
ϕ = phase (the first time when $y = baseline$, in units of x)

For Harmonic Regression, the oscillation period to estimate and test for (Tau) was set to 24, a polynomial was fitted to each time series and used for normalization, and trend elimination was performed. The scalar used to indicate the polynomial degree (both for normalization and trend elimination) was based on the initial visual analysis in GraphPad Prism. Harmonic Regression results were based on statistical significance as determined by Benjamini-Hochberg-adjusted p-values (qvals). For Biodare2 analyses, amplitude and baseline detrending were performed and a period length constraint of 18 to 34h was applied prior to period analysis using each of the six available period analysis algorithms (FFT NLLS, MFourFit, MESA, LS Periodogram, ER Periodogram, Spectrum Resampling; full details at https://biodare2.ed.ac.uk/documents/period-methods). As recommended by the software developers, a positive result (period length of ~22-26h) had to be returned by at least two of these six algorithms, and the rhythmicity analysis (Classic JTK with no detrending and a preset pattern of a cosine curve with 24h period and phases spread every 1h) had to return a statistically significant 'true' result, in order for the data to be assigned as having a 'daily rhythm'. Results for each patient are presented in Supplementary Extended Data and the entire analysis pipeline for each temperature dataset is summarized in Supplementary Fig.1.

Rationale for generalized linear mixed model

A GLMM requires that (1) the outcome is not normally distributed but the distribution is known (here, our outcome variable is binomial 'death or survival'), and (2) that there is more than one source of random error. The outcome itself is not linearly related to the predictor(s) but a function of it (logit for logistic regression) is. In our model, death is defined as a success or 'hit', and survival as a 'miss'. Our observations of 'death' or 'survival' are equal to '1' or '0' and the probability of death p lies between these two values. The logit (log of the odds) is thus a way of linking our GLMM to a non-normal distribution of one of two outcomes. In Fig. 5A-C, plots are provided for those readers who prefer to have a graphical presentation of GLMM outputs that enables visualization of all data points. However, since log of the odds is hard to interpret, and for greater accessibility, Fig. 5D presents the regular odds of death for each predictor. The odds of death = probability of death/(1-probability of death) = $p/(1-p)$. We transform from log odds back to regular odds using the exponential function; for this we also have to transform the 95% confidence intervals and in doing so, we are most interested in whether the confidence interval contains 1 (a confidence interval that contains 0 in log odds will always contain 1 when transformed to regular odds).

Our GLMM is designed as a predictive model (which x variables predict the y variable), not an explanatory one (which x variables are truly related to the y variable). For a predictive model, we are not concerned about confounders if the model makes accurate predictions using the variable of interest. To this end, based on our results, we might consider T_{Br} rhythmicity as a proxy variable; in reality, this variable is most probably directly relevant to outcome, but our model is not designed to test this. There are many other factors that may have influenced temperature and/or outcome in our TBI cohort,[13] however for the question we are asking of our retrospective data, we are not interested in how or why the temperature rhythm exists or does not exist—we are simply asking

whether the presence/absence of a temperature rhythm correlates with odds of death in intensive care. The limitations of patient T_{Br} data currently available internationally precludes absolute adoption of all recommendations for prognostic modelling.[14] Nevertheless, the effect size for mortality presented herein calls for further validation of T_{Br} variation as a prognostic marker in independent datasets, alongside re-appraisal of existing models for TBI.[13]

Supplementary Text

Internal rhythms and health

The body clock—our internal circadian rhythm—anticipates the day-night cycle, thus optimizing every aspect of our physiology to solar time.[15] Circadian rhythms are fine-tuned by environmental cues, which feed into a multi-oscillator system; a 'master' clock within the suprachiasmatic nuclei (SCN) of the hypothalamus synchronizes cell-autonomous clocks throughout the brain and periphery, coupling systemic processes to light-dark transitions.[16] With age, this timekeeping becomes less robust—a situation compounded by modern living, which dissociates our body clocks from natural cues.[17,18] Shift-work, travel, and 'social jet-jag' all disrupt our sleep and circadian health.[9,19] Globally, neurological conditions are the primary cause of disability and second leading cause of death.[20] Several brain disorders including traumatic and vascular events, neurodevelopmental and mood problems, and neurodegenerative diseases are associated with circadian disruption; others, such as epilepsy, display symptomatic coupling to the body clock.[15,21-29] A mechanistic link between circadian and brain dysfunction is yet to be established, but a poorly explored factor is T_{Br}.[15] This is despite the fact that elevated temperature and increased temperature variability are considered prognostic indicators after brain injury.[30-36]

Technical limitations of brain thermometry

Direct T_{Br} measurements from human patients are confounded by variable brain pathology, anaesthesia or sedation, TTM protocols, and/or sampling from subdural or intraventricular sites. MRS brain thermometry is attractive because it is non-invasive and can be used to explore multiple brain locations. However, many patients with moderate to severe TBI (as included in this study) are not suitable candidates for MR-based monitoring. In particular, intracranial Licox probes are not MRI-compatible and in most patients with mild TBI, the placement of an intracranial probe is not required nor ethically justified. Thus, it is not currently possible or safe to simultaneously record T_{Br} from a probe whilst a patient is undergoing MRS brain thermometry. Controversy also remains over the ideal calibration method for MRS thermometry. Common approaches use phantoms to mimic brain tissue, or directly-measured T_{Br} curves from other species under anaesthesia (typically neonates), often reporting T_{Br} values that are substantially lower than direct normothermic T_{Br} measurements. Patient MRS studies have focused on relative T_{Br} changes in response to injury or other pathology.[34,37-43] At the time of writing, we are aware of 25 published original research articles that used MRS for brain thermometry in healthy adults (Supplementary Table 1). Sex was reported in 22 of these studies; 21 of them included <36 participants per experiment—a total of 329 participants including 105 women. Only two studies made sex comparisons for T_{Br}, both reporting no statistical difference.[44,45] One retrospective study (n=150 with 90 females) found a 0.1°C higher cerebral T_{Br} in females[46]; smaller than the sex difference found in our cohort. However, no prior studies controlled for menstrual cycle phase, which has

enabled us to reveal physiologically-relevant differences between the sexes in all brain regions measured. In addition to menstrual cycle, the large retrospective study of Maudsley et al.[46] did not control for age, time of day, chronotype, or medications, all of which may have contributed to high variance in their dataset. Eight MRS brain thermometry studies reported time period of scanning, but only 6 of these arguably controlled for time of day (scanning window 2–3 hours).

Recent studies have emphasized the need for more accurate calibration and potentially brain region-specific temperature coefficients to account for differences in tissue content, microstructure and orientation.[10,46-48] However, using methods described by Marshall[40] and Thrippleton et al.[10] we have now obtained data that are absolutely consistent with published direct T_{Br} measurements obtained from healthy, conscious, non-human primates, and normothermic human patients.[49-52,53] The limitations of MRS thermometry dictate that 'absolute' temperatures cannot be estimated with the same confidence as relative temperature differences. That said, the mean global temperatures we have observed lie, as expected, between average human core T_{Bo}[54,55] and direct brain temperatures obtained from other species and human patients with brain injury (Supplementary Fig. 3).[49,52,56,57] To avoid concerns arising from potential inaccuracy of the calibration intercept, we have focused our analysis on relative T_{Br} changes, for which MRS thermometry is exquisitely well-suited.[10] Given the propensity for major brain structural changes with age and neurodegeneration, exploring time of day variation at different life stages, rather than single time-point variation over months or years, would circumvent some of the inherent limitations of the technique. Ultimately, physiological and clinical understanding should be informed by experimental observation. MRS thermometry has high spatial resolution and is extremely sensitive to temperature change within a tissue, but the technique can only ever have low temporal resolution. Moreover, it cannot be used for repeated measurements of brain-injured patients; whereas, direct temperature probes measure absolute temperature with very high temporal resolution, but cannot be used in healthy individuals. We have found these two approaches of T_{Br} measurement to be mutually supportive, with the temperature probe data and repeated measurements of individuals allowing us to be much more confident about the fidelity of the MRS data than in any previous study. Temperature variations were further supported by high temporal resolution actigraphy data in our healthy cohort, and core T_{Bo} data in TBI patients.

Temperature gradients

In our healthy cohort, T_{Br} was always higher than oral temperature. The direction of this brain-body temperature gradient contradicts some studies,[58,59] but agrees with others.[44,58,60] Our findings are corroborated by a case report documenting parallel oesophageal, tympanic, intraventricular, and cortical white matter temperature in an unanaesthetised, normothermic patient.[53] The relationship between sites was fixed, with cortical T_{Br} > ventricular > oesophageal > tympanic.[53] During an overnight recording, cortical T_{Br} declined from ~38.1°C at 9pm to a minimum of ~36.7°C at 3am, as expected during sleep. Considering this 1.4°C decrease over 6 hours, and extrapolating back to the predicted maximum T_{Br} for this male patient (~3pm), maximum T_{Br} in this region would have been ~38.7°C—practically indistinguishable from the mean T_{Br} we found in healthy male parietal white matter in the afternoon (Fig. 4). In the report, T_{Br} exceeded oesophageal temperature by ~0.7°C, and so our measured difference between oral temperature and T_{Br} is realistic. A similar brain-body temperature relationship was found in healthy volunteers with a mean parietal MRS-derived T_{Br} of 38.1°C; 1.3°C higher than rectal temperature.[45] Our retrospective analysis indicates that a positive T_{Br}-T_{Bo} gradient cannot be attributed entirely to local

injury (Supplementary Fig. 2) and so, as for non-human primates,[49,50] it is likely a normal physiological phenomenon in humans. Indeed, maintenance of this gradient was associated with a better outcome in severe head injury.[61]

MRS-derived estimates of absolute T_{Br} often conflict with known T_{Br}-T_{Bo} gradients in other species, including non-human primates.[49,50] A recent 7T MRS-based T_{Br} study in healthy sheep found a brain-regional T_{Br} gradient, but global T_{Br} was 0.7°C lower than T_{Bo} (38.5 versus 39.2°C).[56] This inversion of the brain-body temperature gradient might be explained by several factors: (i) that measurements were conducted under general anaesthesia (which inverts temperature gradients in some species), (ii) the species under test has defined neurovascular anatomy to effect selective brain cooling (SBC; a carotid *rete mirabile* is absent in primates and the existence of other SBC mechanisms in humans and non-human primates is highly contentious),[49,52,62,63] and (iii) it remains possible that absolute mean global T_{Br} is the key defended parameter across mammals, whilst T_{Bo} can vary more widely in a species-specific manner (T_{Bo} of most domestic livestock is higher than human T_{Bo}). In seminal work by Fuller et al., diurnal variations in colonic and hypothalamic temperatures were explored simultaneously in unsedated male squirrel monkeys.[52] At various ambient temperatures (T_a of 20, 26, 32, and 36°C), there was no change in the phase or period length of either temperature rhythm, and hypothalamic always exceeded colonic temperature except at T_a 36°C. Mean temperatures at both sites increased with increasing T_a, and there was a clear reduction in amplitude in both temperature rhythms, primarily resulting from an increase in temperature minima during the night. Importantly, hypothalamic temperatures ranged from 37.5–39.1°C between the afternoon and late evening at T_a 26°C, remarkably similar to the hypothalamic temperature range observed by MRS in male subjects here (Fig. 4).[52] Compelling evidence for the absence of SBC in primates comes from a study in unrestrained baboons, where hypothalamic temperature was consistently higher than carotid arterial or abdominal temperature, ranged between 37.5 and 38.8°C under cycling T_a conditions (15–35°C), and from 37.5–40.5°C when water was restricted.[49] Our results for this brain region are thus entirely consistent with direct temperature measurements from conscious healthy animals of closely related species.

Temperature rhythms and sleep

Temperature rhythms emerge immediately after birth and peak in amplitude during childhood as the brain matures.[15] T_{Bo} rhythms then phase-delay in adulthood, and gradually reduce in amplitude and phase-advance with ageing.[15] Daily variations in heat loss (rather than heat production) are considered to drive these daily changes in temperature[64]; it follows that an increase in minimum T_{Br} (and consequently, a reduced T_{Br} rhythmic amplitude) with ageing must represent a failure of heat loss. It has been estimated that T_{Br} would increase by ~0.28°C/min if the key routes of heat dissipation did not exist,[65] so even modest cerebrovascular compromise is likely to result in net heat gain. This is not necessarily at odds with findings in ischaemic stroke, where T_{Br} is higher in the lesion penumbra than the lesion core.[35,40] At the core, total loss of perfusion would lead to a T_{Br} drop as observed during brain death.[66] A vascular-driven disruption of T_{Br} rhythm reconciles various risk factors for non-convulsive seizures during sleep in dementia patients with some of the temperature-sensitive molecular aberrations in animal models of these disorders,[67,68] and potentially even disease-specific distribution of protein pathology. Rather than an inherent vulnerability of specific, synaptically-connected neurons, or an age-related decline in glymphatic clearance,[19] the predictable 'spread' of protein aggregates through the brain in neurodegenerative disease may simply reflect age-related progression of cooling failure through vascular

compromise. This is conceptually straightforward for chronic traumatic encephalopathy, where tau pathology is concentrated at cerebral sulci surrounding repetitive microvascular trauma.[69] The propensity of mutant protein forms to aggregate in familial disease may signify a heightened sensitivity to temperature-driven effects on biochemical modification in regions of greatest diurnal T_{Br} variability.[70,71] Notably, the hypothalamus (and in particular the SCN) is one of the most densely vascularized regions of the mammalian brain.[72] This is the first study to measure T_{Br} in the human hypothalamus; future studies should consider whether the position and vascularity of this structure optimizes its circadian and thermoregulatory roles, whilst shielding it from high absolute temperatures. Epilepsy is bimodally distributed but the highest incidence is in adults >75 years, where cerebrovascular disease causes more than a third of cases.[67,73] Alzheimer's disease and late-onset epilepsy may be pathologically linked through vascular changes, tau pathology, or both, and sleep deprivation can unmask a life-long tendency to seizures.[67] Given the temperature-sensitivity of tau protein modifications, the role of brain vasculature in effecting T_{Br} changes, and the importance of temperature in sleep, impaired T_{Br} rhythms might contribute to this pathological link.[70,74] In everyday clinical terms however, cerebrovascular impairment is not the only route to a T_{Br} increase; nasal airflow is clearly inhibited during prolonged wearing of facemasks,[75,76] or indeed bypassed all together in intubated patients. That daily T_{Br} variation is such as strong predictor of mortality (Fig. 5) underpins an urgent need for further chronotype-controlled research in this area.[30,53]

Superimposed on daily temperature rhythms is a daily oscillation in thermoregulatory capacity which is relatively impaired during sleep.[64] This may have contributed to the increased variability in T_{Br} in participants that reportedly slept during their scans (Fig. 3B). Whilst the preoptic area and anterior hypothalamus are the main loci for T_{Bo} regulation, their thermoregulatory function appears to suppress, rather than coordinate, the circadian organization of T_{Bo}—a tenet of the SCN.[15] Studies in several mammalian species including humans have suggested that circadian T_{Br} and sleep-wake cycles are not interdependent, but that under free-running conditions, the period length of the sleep-wake cycle is negatively correlated with average core T_{Bo}.[77-81] In rats, ultradian (<24-hour) T_{Br} rhythms are superimposed on circadian T_{Br} rhythms.[82] These high-frequency rhythms are closely associated with sleep-wake states, and persist in the absence of SCN input which abolishes the circadian rhythm in T_{Br}.[82] Rodents however are not small humans; there are critical differences in thermoregulation strategy and rate of heat gain/loss across mammals which make it difficult to translate temperature-related phenomena between species of vastly different sizes.[83] Such caveats aside, it is clear that changes in T_{Br} can affect synaptic structure, function, and plasticity in mammals,[84,85] and may even influence the diurnal variation in glymphatic clearance of 'brain waste'.[19,86] This clearance in awake rats seems to be greatest during the rest phase, at a time when T_{Br} would be lowest and CBF increased.[86-88] On the one hand, increased CBF might simultaneously enhance the removal of brain heat whilst favouring pulsatile glymphatic clearance; on the other it could reduce the perivascular space available for glymphatic flow through hydrostatic means, particularly in the presence of hypertensive cardiovascular disease.[19] We found no significant correlation between T_{Br} and subject-reported sleep during scanning (Supplementary Appendix 5). However, we did not employ a gold standard method for sleep measurement (polysomnography) and since the scan time was around only 30 minutes (and the MRS acquisition much less than this), we would not expect to observe a significant effect. Irrespectively, our late evening T_{Br} data confirms that sleep onset coincides with a reduction in both core T_{Bo} and T_{Br}, assisted by a selective redistribution of blood flow (Fig. 2B-C), and presumably modulated by melatonin.[64,89,90-92] Moving

forward, it will be important to consider the role of this hormone, and whether its rhythmic disruption in intensive care patients affects its rhythmic differential modulation of regional vasculature.[92,93]

Limitations of retrospective TBI analysis

There are many factors (internal and external to the patient) that might impact upon T_{Bo}, T_{Br}, or mortality risk in TBI patients.[13] We could only test for relationships among the limited number of parameters for which data were available in our patient cohort, and that could be accommodated by our sample size without overfitting the outcome model. Our model thus posed a very simple question of whether the presence or absence of a daily T_{Br} rhythm correlates with the odds of death, without implying causality in either direction. Despite our inability to adjust for several more conventional parameters such as injury severity, our analysis suggests that loss of a daily T_{Br} rhythm is a very powerful predictor of mortality compared to prognostic factors reported historically, either alone, or in combination.[13] One interpretation is that a daily T_{Br} rhythm together with age might be a very simple way to accurately predict ICU outcome after brain injury—independent validation and a rigorous prospective clinical study is of course needed test this hypothesis.[14] The fact that presence of a daily T_{Br} rhythm did not correlate with either age or mean T_{Br} indicates that this parameter has additional predictive value in its own right.

Clinical applications

Impaired daily core T_{Bo} rhythmicity correlates with injury severity and worsened behavioural measures for patients in vegetative or minimally conscious states.[21,22] One study reported that only 29% of trauma patients had a T_{Bo} rhythm period of 22–26h, and circadian disruption has been associated with poor outcome after both head trauma and subarachnoid haemorrhage.[94,95] T_{Br} was not measured in these patients, but our findings (Fig. 5) suggest that relationships between temperature rhythm and outcome would be clearer if T_{Br} was included for monitoring and prognostication. Indeed, functional scoring of comatose patients varies by time of day, with better scores around maximum T_{Bo}.[94] Based on our new data, we recommend review of existing guidelines for management of TBI patients in intensive care. Specifically, we propose that direct T_{Br} (in addition to core T_{Bo}) becomes the clinical standard for temperature monitoring in these patients, and that, alongside other clinical parameters, daily *temperature variation* should be considered a core element of treatment plans and prognostication. At the very least, time of day should be considered when scheduling assessments, and ideally, patient-specific temperature rhythm and chronotype should be accounted for when interpreting results that influence life-changing clinical decisions. Future studies should standardize the method of T_{Bo} measurement, and the location of intracranial probes with respect to focal brain pathology. By contrast, HEATWAVE would find greatest practical utility for chronic brain disease, since our voxel maps give an approximation of the T_{Br} expected in each brain location at three clinically-relevant times of day, and how much T_{Br} should vary between voxels at a given time point. Since each data point in each map is averaged from multiple healthy individuals, it incorporates the range of ages, BMIs, and chronotypes for each sex in the demographic tested.

Beyond brain injury and disease, our results further question the value of single-point temperature measurements using peripheral thermometers.[96] We have shown that more sophisticated analyses can better exploit temperature as a clinical tool. Wearable devices now permit easy and convenient recording of daily rhythms in many physiological parameters. Algorithm-based temperature

profiling will help accomplish the goals of precision medicine, not just for individuals,[54] but at scale. For example, in an infectious disease outbreak, real-time screening for fever development could rapidly identify high-risk individuals by deviation from their own temperature rhythm, rather than a population 'mean' or by random testing. Personalized, digital, round-the-clock temperature monitoring would thus advance remote health tracking and evidence-based enforcement of global health policy in the context of emerging disease.

Supplementary Figures and Tables

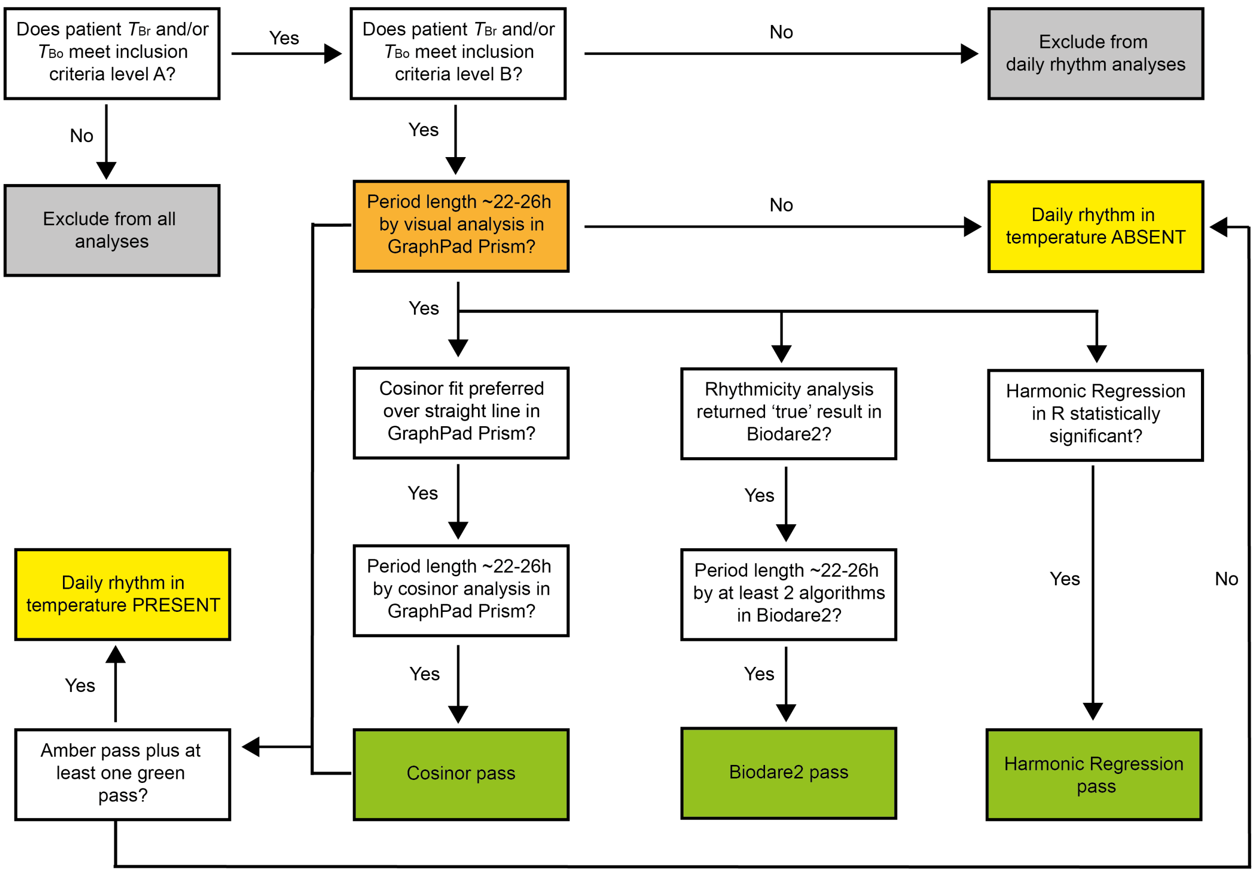

Supplementary Figure 1 Patient temperature analysis pipeline.

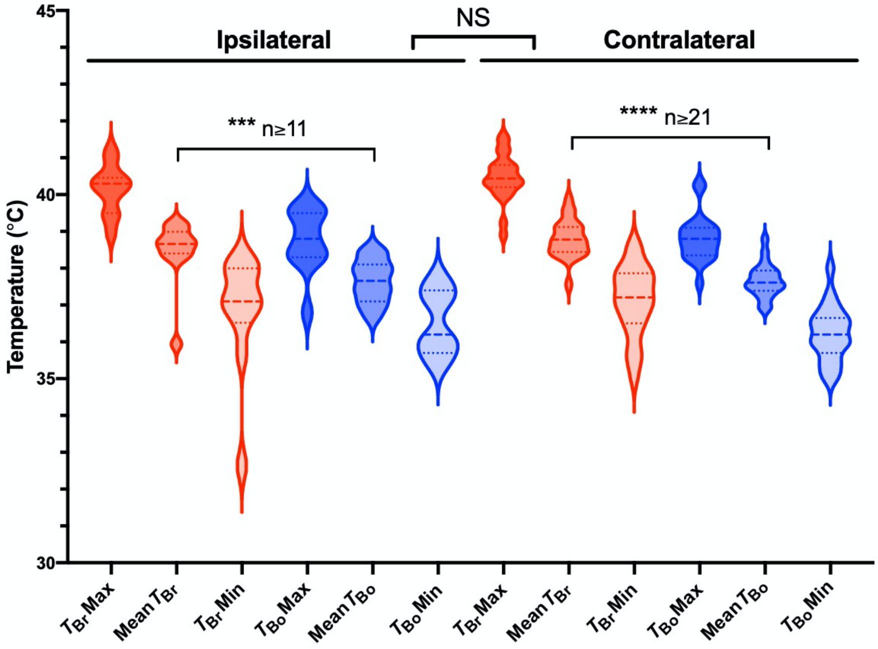

Supplementary Figure 2 Patient temperatures according to probe site. Violin plot of T_{Br} and T_{Bo} in TBI patients with focal brain injury. The nature of brain injury was available for 46 patients (34 focal, 12 diffuse); of the focal injury cases, the brain probe was placed ipsilateral and contralateral to the site of injury in 12 and 22 cases, respectively. Mean T_{Br} was greater than mean T_{Bo}, regardless of site of probe placement (mixed effects analysis with Tukey's for multiple comparisons ***P=0.0007, ****P<0.0001, n refers to number of individual patients; one patient in each probe site group lacked T_{Bo} data). There were no significant differences in temperature according to site of probe placement.

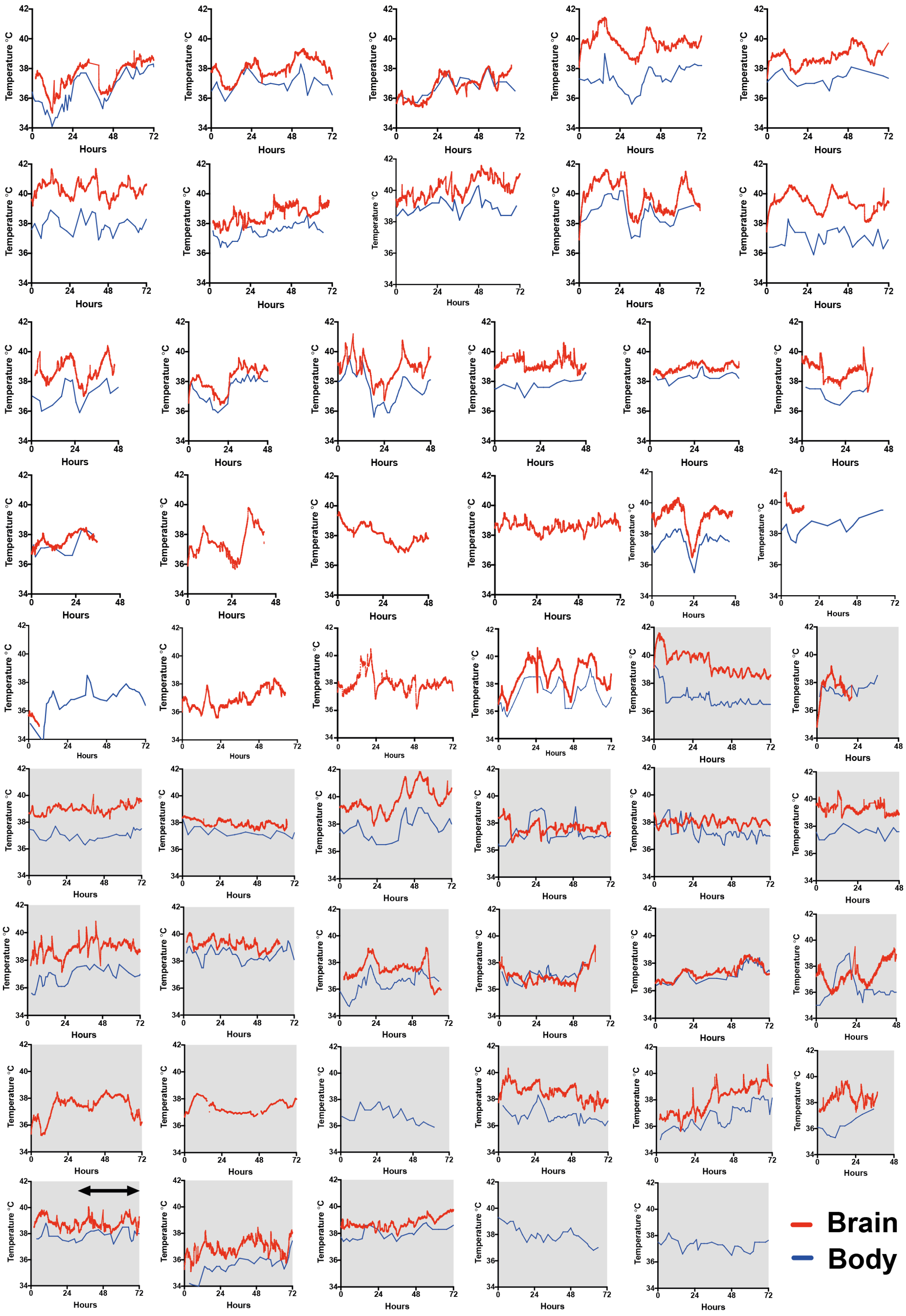
Temperature °C
Hours
Brain
Body

Supplementary Figure 3 Raw temperatures in non-survivors and patients with daily rhythms. Upper panel (white plots) show raw T_{Br} and/or T_{Bo} data from 26 patients that survived intensive care and were deemed to have evidence of a daily rhythm in one or both temperature parameters at some point during recording. Lower panel (grey plots) show the 25 patients that did not survive in intensive care after TBI. All but one of these patients (lower left) showed a complete lack of a daily temperature rhythm during recording. Rhythmicity was apparent in some of these patients but it did not meet our assigned daily cut-off for period length of 22–26h. Note that in the patients who died, transitory inversion of the T_{Br}–T_{Bo} gradient was more commonly observed than in those that survived (in which T_{Br} was almost always higher than T_{Bo}). The patient in the lower left corner was classified as having a daily rhythm based on the time period highlighted by the horizontal black arrow, although it is clear that the period length was >26h earlier on in the recording.

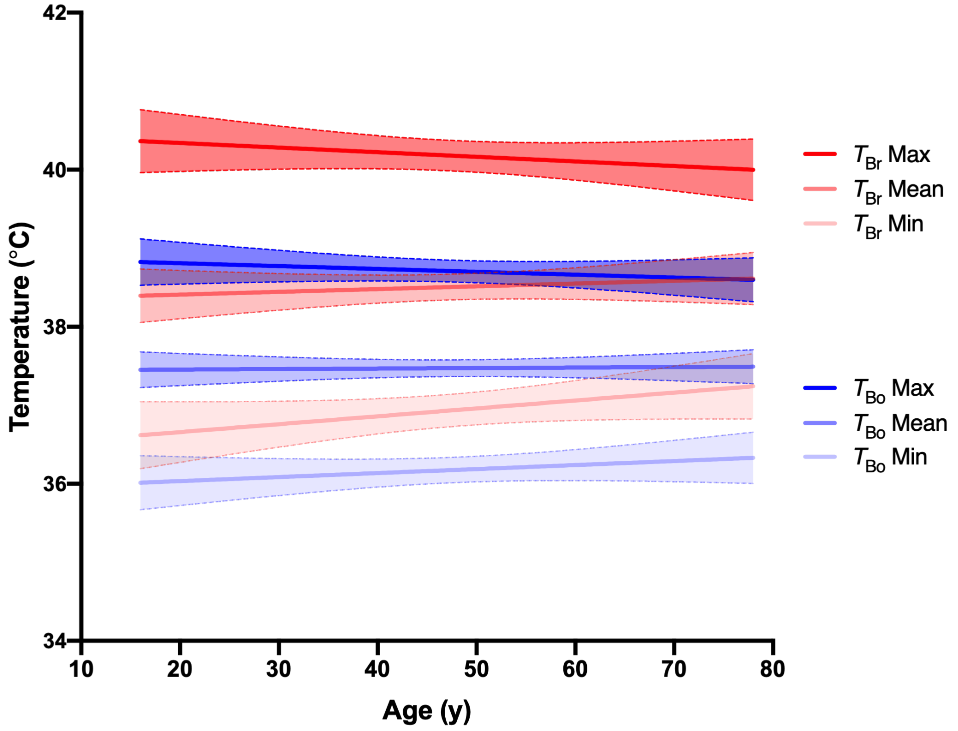

Supplementary Figure 4 Minimum brain temperature trends upwards with age in TBI patients. Linear regression of patient temperatures with age (n=105 for T_{Br}Mean, n=104 for T_{Br}Max and T_{Br}Min, n=101 for T_{Bo}Max and T_{Bo}Min, n=94 for T_{Bo}Mean); the apparent upward trend for minimum T_{Br} was not significantly different from zero (slope 0.010, 95% confidence interval -0.002 to 0.022, P=0.096). Shaded areas represent 95% confidence intervals.

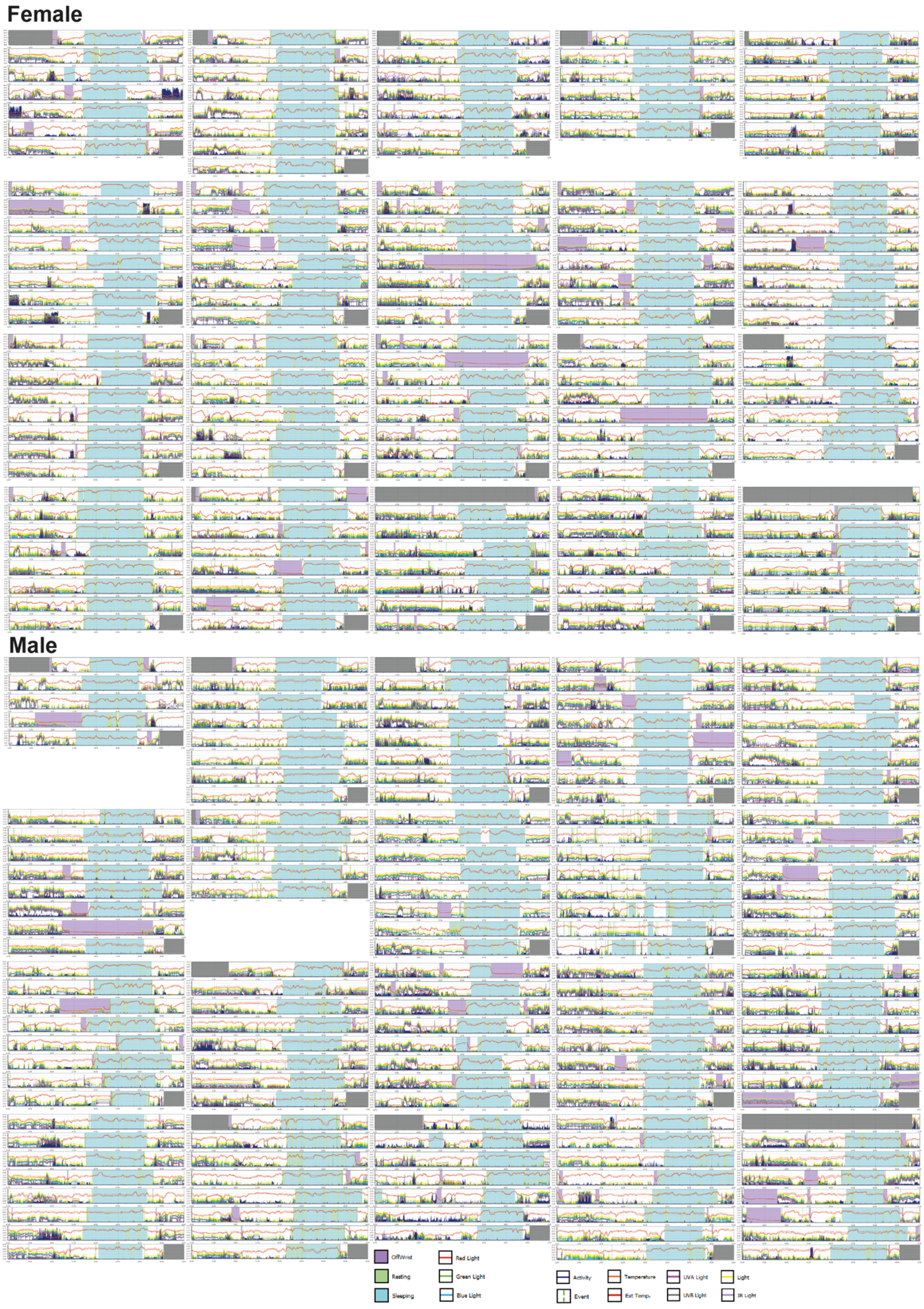
Female
Male
OffWrist
Resting
Sleeping
Red Light
Green Light
Blue Light
Activity
Event
Temperature
Ext Temp.
UVA Light
UVB Light
Light
IR Light

Supplementary Figure 5 Actograms in healthy volunteers. Montage of actograms in 40 healthy volunteers. Most participants wore an ActTrust2 device for eight days. Actograms are presented in order of acrophase (earliest top left, latest bottom right) in females and males separately. Note increase in participant wrist skin temperature during sleep phase, reflecting peripheral vasodilation (core body temperature is expected to drop in anti-phase to a skin temperature increase during sleep).[97]

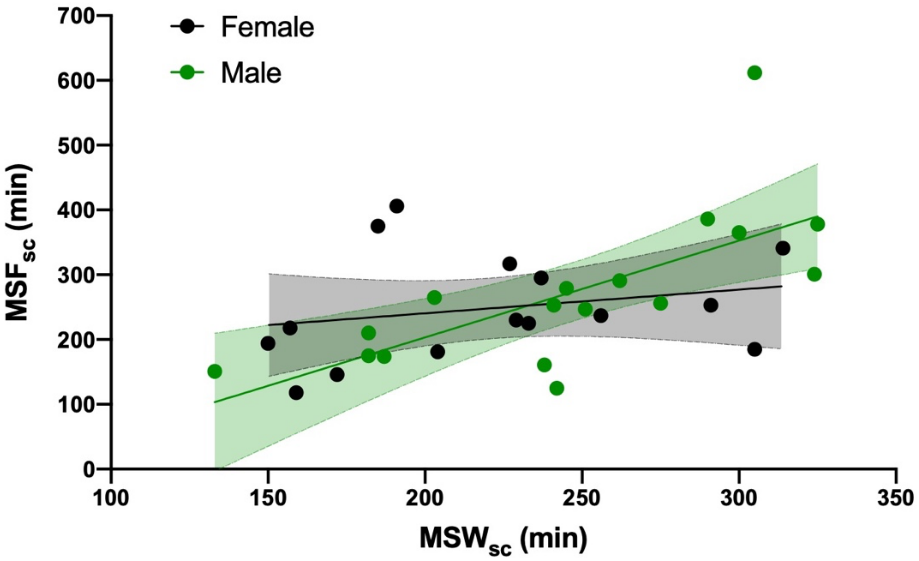

Supplementary Figure 6 Interpolation of MSF_{sc}. Five females and three males reported no free days during actigraphy sampling; their sleep-corrected midpoint of sleep on free days (MSF_{sc}) was interpolated by linear regression with the sleep-corrected midpoint of sleep on work days (MSW_{sc}). Regression was performed for males and females separately.

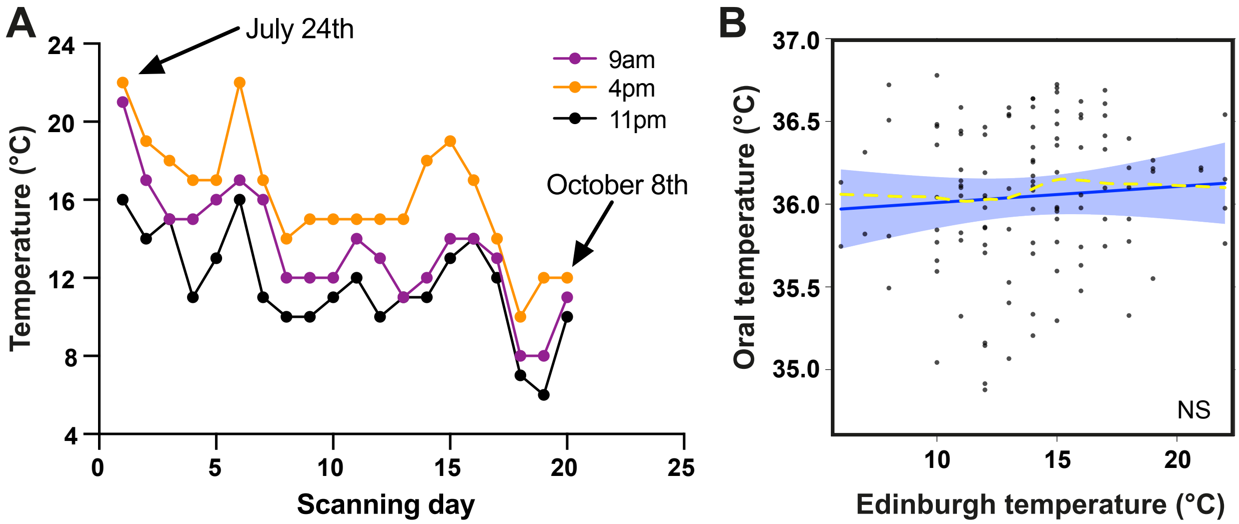

Supplementary Figure 7 Environmental temperature variations. (**A**) Recorded outdoor air temperatures at 1.5m above ground level in Edinburgh at times of scanning sessions, on each day of scanning during data collection period. Collection period limited to 14 weeks to minimize any effect of seasonal variation in environmental temperature and light. For actigraphy data extraction from each participant, daily changes in sunrise and sunset times were adjusted according to date of device retrieval using date and location function in ActStudio. (**B**) Linear mixed model results for oral temperature by environmental temperature. Solid blue line represents model fit, shaded areas represent 95% confidence intervals, dark grey circles display residuals (single temperature data points), and smoothed dashed yellow line represents partial residuals.

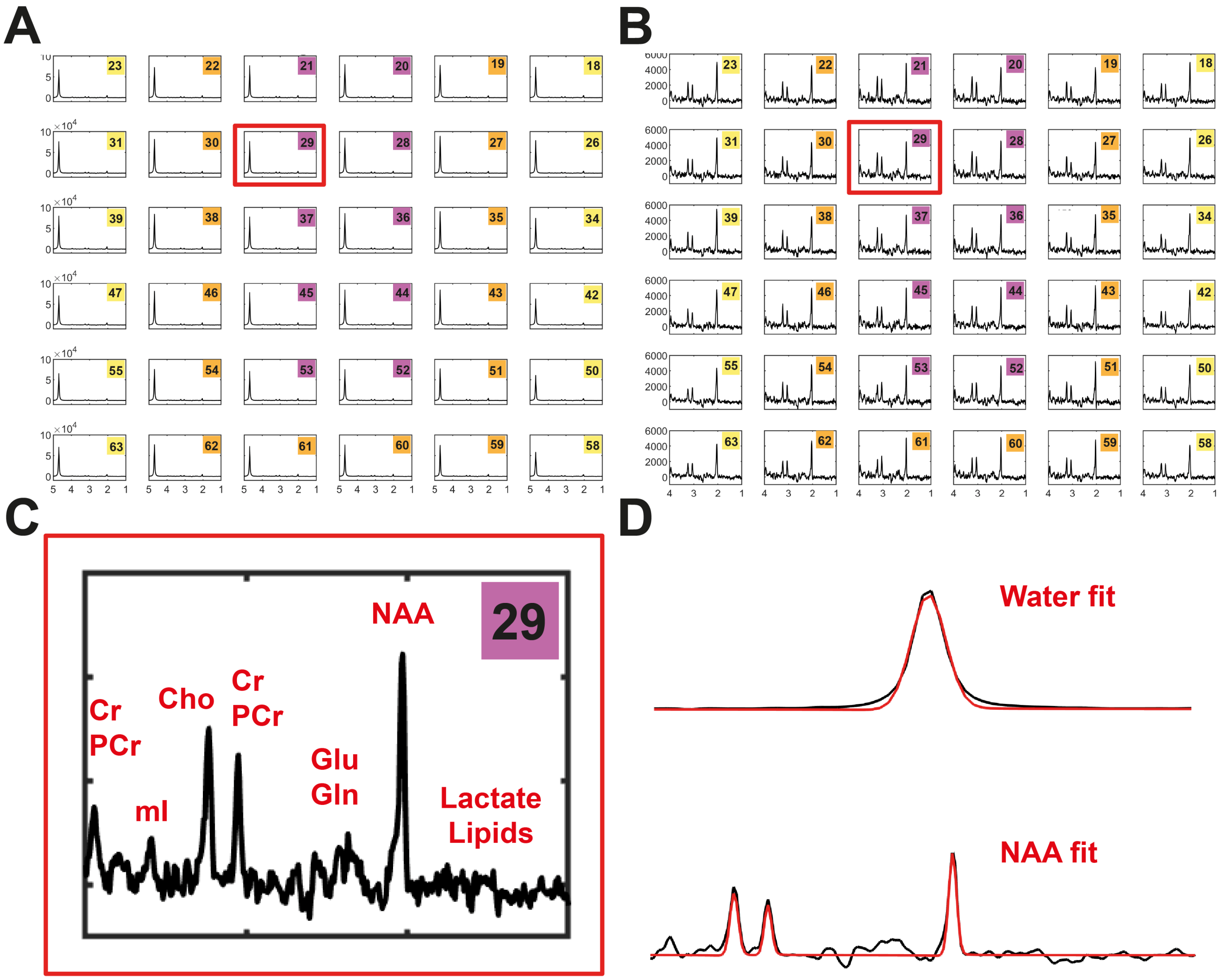

Supplementary Figure 8 MRS data processing. (**A**) Example spectra from central 36 voxels from cerebral region; voxel numbering as in Fig. 3. At this scale, only the large water peak to the left of each spectrum is clearly visible; smaller peaks to the right of this are barely visible. Selected voxel (29) shown in red box. (**B**) Zoomed-in representation of voxel 29, focusing on smaller peaks; the water peak is off-chart (and off-scale) to the left. The largest peak visible in the spectrum is now NAA. (**C**) Enlargement of voxel from (**B**) to show peak annotation; Cr, creatine; PCr, phosphocreatine; mI, myo-inositol; Cho, choline; Glu, glutamate; Gln, glutamine; NAA, N-acetylaspartate. (**D**) Representative examples of spectral fitting of water and NAA peaks for voxel 29. All but one of the 24 rejected T_{Br} data points were obtained from the most rostro-lateral voxel of the right frontal lobe.

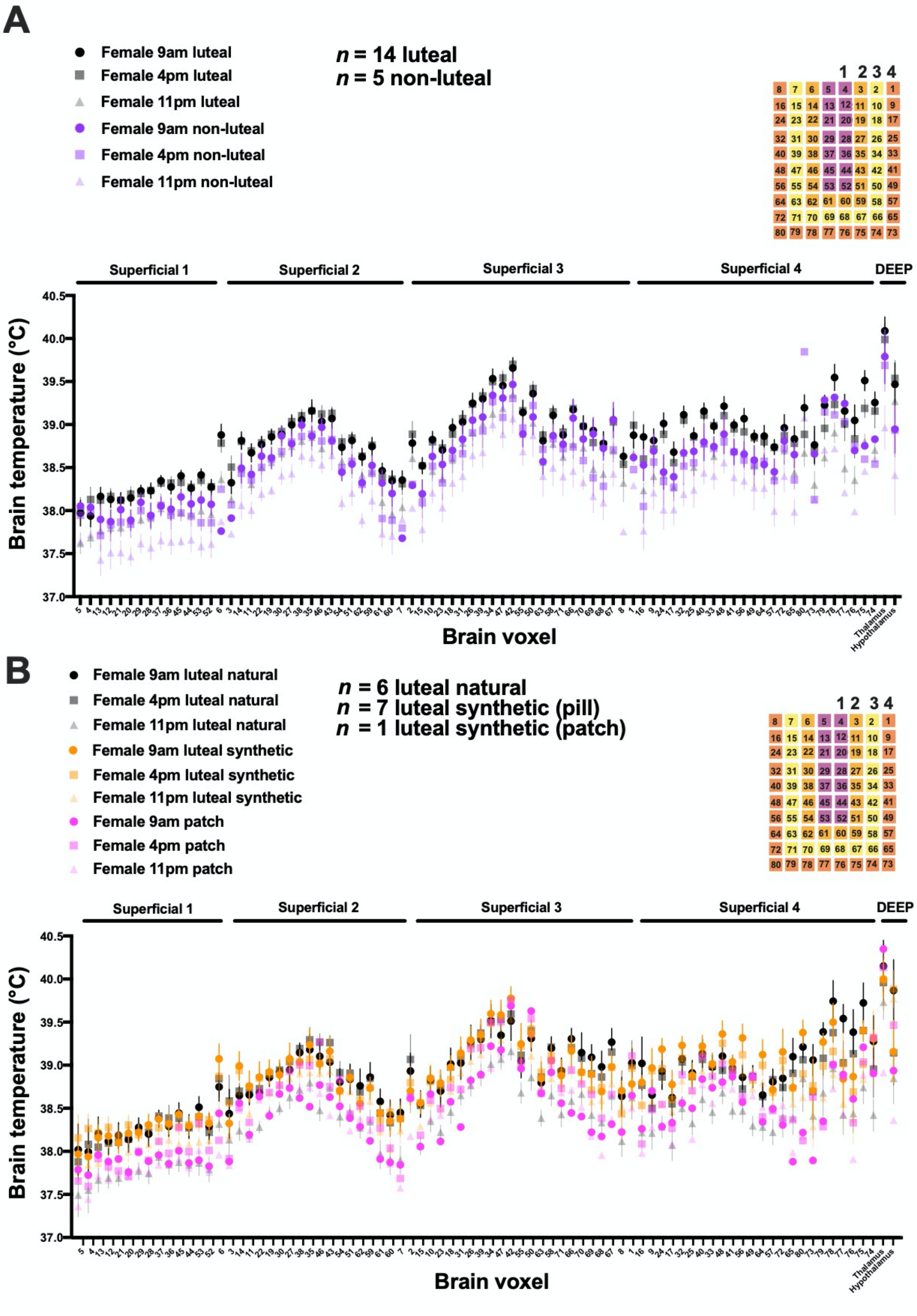
A
Female 9am luteal
Female 4pm luteal
Female 11pm luteal
Female 9am non-luteal
Female 4pm non-luteal
Female 11pm non-luteal
n = 14 luteal
n = 5 non-luteal
Superficial 1
Superficial 2
Superficial 3
Superficial 4
DEEP
Brain temperature (°C)
Brain voxel
Thalamus
Hypothalamus
B
Female 9am luteal natural
Female 4pm luteal natural
Female 11pm luteal natural
Female 9am luteal synthetic
Female 4pm luteal synthetic
Female 11pm luteal synthetic
Female 9am patch
Female 4pm patch
Female 11pm patch
n = 6 luteal natural
n = 7 luteal synthetic (pill)
n = 1 luteal synthetic (patch)
Superficial 1
Superficial 2
Superficial 3
Superficial 4
DEEP
Brain temperature (°C)
Brain voxel
Thalamus
Hypothalamus

Supplementary Figure 9 Female brain temperature by menstrual cycle phase. (A) Female T_{Br} data split into luteal and non-luteal groups. Menstrual cycle phase explained 15.2% (P=0.0006) and time of day explained 21.2% (P=0.0004) of the variation in T_{Br}, respectively (two-way ANOVA). The interaction between these two factors was not significant. (**B**) Luteal-phase female T_{Br} plotted according to contraception type. T_{Br} did not differ between luteal females with natural menstrual cycles and those taking the combined contraceptive pill (mixed-effects analysis with Tukey's multiple comparisons test). Synthetic steroid hormones influence T_{Bo} differently from endogenous forms; women taking oral contraceptives have persistently raised T_{Bo}, similar to naturally cycling women in the luteal phase.[53] We suggest this is also true for T_{Br}. Only one participant was using the combined patch; note that T_{Br} for this participant was generally lower (more consistent with non-luteal females and males). For each voxel in (**A**) and (**B**), data are plotted as mean ±SEM. Voxel numbering on x-axis refers to numbers shown in overlay (top right); voxels were grouped into 4 concentric U-shaped regions labelled 1–4 based on T_{Br} variation. Note relatively high T_{Br} in cerebral white matter ('Superficial 3') and thalamus, and low T_{Br} in grey matter surrounding the sagittal sulcus ('Superficial 1').

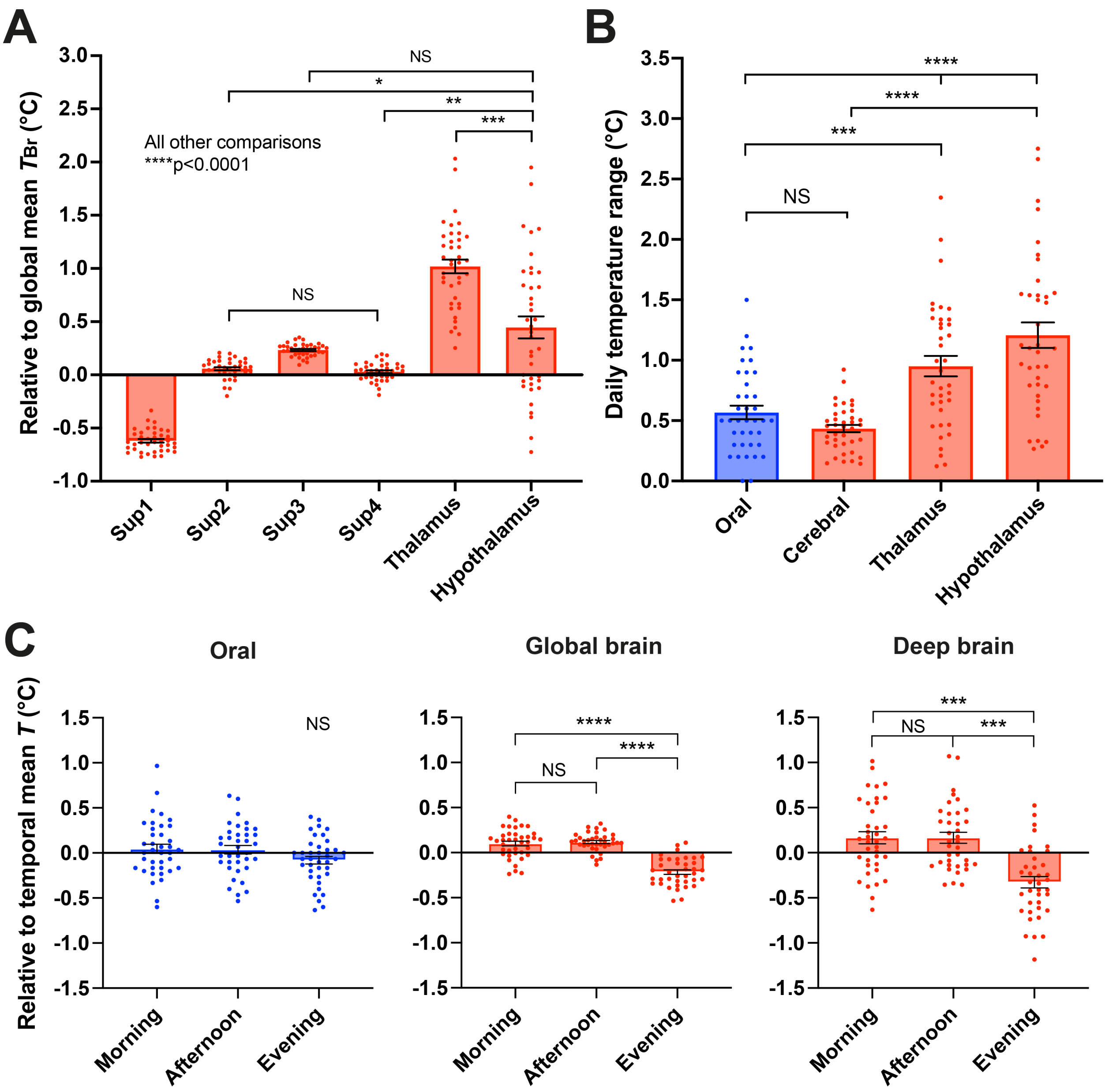

Supplementary Figure 10 Daily temperature ranges by location. (**A**) Time-averaged spatial variation in T_{Br} by brain region; temperature deviations relative to global temporal mean T_{Br} were calculated for each individual. Sup1-4, superficial cerebral layers 1-4. Comparisons between regions were significant unless specified as NS (n=39, repeated measures one-way ANOVA with Tukey's multiple comparisons test, *P=0.01, **P=0.004, ***P=0.0002, ****P<0.0001). (**B**) Daily temporal temperature ranges (maximum versus minimum across the three tested time points) are plotted by measurement site (n=39). Note that temperature varied more by time of day in the thalamus and hypothalamus than in the cerebrum or orally (repeated measures one-way ANOVA with Sidak's multiple comparisons test ***P=0.0004, ****P<0.0001). 'Cerebral' refers to mean T_{Br} from all cerebral voxels combined. (**C**) Temporal variations in temperature; temperature deviations relative to temporal mean were calculated for each participant for three sites (body, global brain, deep brain) at each time point. Note drop in T_{Br} in late evening (n=38, mixed-effects

analysis (REML) with Tukey's multiple comparisons test, ****P<0.0001, ***P=0.0004 for morning versus evening and 0.0001 for afternoon versus evening). NS, non-significant. Note that analyses in (**A**) to (**C**) were performed without controlling for sex, age, BMI, menstrual cycle stage, or chronotype. Irrespectively, the effects of brain region and time of day on T_{Br} remain significant.

Supplementary Box 1 Definitions for key terms and phrases with context-dependent meaning

Term	Definition
Circadian	'About a day'; here also used as an adjective to describe physiological phenomena that vary in a repeating, approximately 24-hour pattern as a result of the internal timekeeping mechanism of a human (the 'body clock'), but which cannot be formally confirmed to be 'circadian' in nature unless observed/measured under constant environmental conditions. None of the data collected from healthy volunteers or TBI patients in this study can be described as circadian, but a subject/patient's circadian rhythm ('body clock') will have impacted on the key variables observed/measured.[a]
Circadian rhythm	A self-sustained cyclic biological phenomenon that (i) persists under constant conditions with a period length of around 24 hours, (ii) can be entrained by or synchronises to environmental cues, and (iii) proceeds at approximately the same rate (same period length) across a physiological range of temperatures for a given organism.[b]
Daily	Something that happens once every day or varies in an approximately 24-hour pattern.
Daily rhythm	A cyclic phenomenon that repeats every day.[c]
Diurnal	Something that changes by time of day in the manner expected for a daytime active mammal (e.g. a human) under natural environmental conditions.
Diurnal rhythm	A cyclic phenomenon that repeats approximately every 24 hours in the manner expected for a daytime active mammal (e.g. a human) under natural environmental conditions.[d]
Diurnal variation	A parameter that varies with time of day in the manner expected for a daytime active mammal (e.g. a human) under natural environmental conditions.
Rhythmic variation	A parameter that varies in a repetitive and predictable manner.
Spatiotemporal variation	A parameter that varies with space and time; in the context of this work, a change that happens according to different brain locations, at different times of the day, and/or according to age of the subject/patient.
Time of day variation	A parameter that varies according to the (external) clock time at which it is observed/measured which may also be influenced by the phase of the (internal) circadian rhythm ('body clock') at which it is observed/measured.

[a]'Circadian' is used also in reference to cited works that have used this specific term.
[b]This means that the rhythm is temperature compensated.
[c]A daily rhythm may also prove to be a circadian rhythm if observed/measured under constant environmental conditions; such conditions do not apply to the data described in this manuscript.
[d]A diurnal rhythm may also prove to be a circadian rhythm if observed/measured under constant environmental conditions; such conditions do not apply to the data described in this manuscript.

Supplementary Table 1 Published studies using MRS brain thermometry in healthy adults

Study	Subject age	Females/total	TOD control	T_{Br}	Calibration method
Childs et al. (2007)[58]	23–52y	3/8	No	36.5	Anaesthetised piglets[59]
Corbett et al. (1997)[59]	NR	5/10	No	37.2/37.7[a]	Anaesthetised piglets[59]
Corbett & Laptook (1998)[98]	22–47y	NR/10	No	37.0/36.6[b]	Anaesthetised piglets[59]
Curran et al. (2017)[99]	Mean 33y	NR/6	No	NA	Phantom[d99]
Covaciu et al. (2010)[45]	18–57y	5/18	No (10:30–17:30)	38.1	Phantom[e45]
Covaciu et al. (2011)[100]	21–62y	2/10	No	NA	Anaesthetised adult pigs[101]
Fujiwara et al. (2016)[102]	21–50y	3/15	No	38.3[c]	Anaesthetised piglets[103]
Harris et al. (2008)[104]	31–48y	2/5	No	NA	Phantom[f40]
Inoue et al. (2013)[105]	NR	NR/5	No	37.1	NR
Kaupinnen et al. (2008)[106]	21–51y	5/14	No	37.4	Anaesthetised piglets[103]
Kickhefel et al. (2010)[107]	25–43y	5/9	No	NA	Ex vivo swine muscle
Marshall et al. (2006)[40]	23–38y	0/4	No	36.5	Phantom referenced[f40]
Maudsley et al. (2017)[46]	18–84y	90/150	No	37.5	Combination[103,48,108,109]
Onitsuka et al. (2018)[110]	Mean 26.9	0/8	No	~37.2	Anaesthetised piglets[103]
Posporelis et al. (2018)[44]	Mean 23.1y	7/20	Yes (12:00–14:00)	NA	Anaesthetised piglets[103]
Rango et al. (2012)[41]	49–78y	5/10	Yes (14:00–16:00)	36.8	Anaesthetised piglets[59]
Rango et al. (2014)[65]	Mean 43y	6/14	Yes (14:00–16:00)	37.6	Anaesthetised piglets[59]
Rango et al. (2015)[111]	23–53y	10/20	Yes (14:00–16:00)	37.38	Anaesthetised piglets/rats[59,111]
Sharma et al. (2020)[112]	23-46y	7/18	No	37.2	Combination[103,48,108,109]
Shiloh et al. (2008)[113]	Mean 30.6y	0/10	No	37.7	Anaesthetised piglets[103]
Sumida et al. (2016)[114]	25–78y	18/35	Yes (17:00–19:00)	36.04	Anaesthetised piglets[103]
Thrippleton et al. (2014)[10]	22–40y	0/51	Yes (afternoon)	37.4	Referenced phantom[f40]
Verius et al. (2019)[37]	22–37y	15/30	No (08:00 to 15:50)	37.78	Phantom[g37]
Weis et al. (2012)[115]	20–61y	2/10	No	NA	Anaesthetised adult pigs[115]
Zhang (2020)[116]	19-49y	5/10	No	36.9	Combination[103,48,108,109]

Subject age is reported as available in publication; either mean or range. Females/total states number of female subjects included relative to total number of subjects, where this information was available. NR, not reported; NA, not applicable; TOD, time of control. 'No' means there was no reported control for TOD when scanning subjects; time period during which scans were performed are given in brackets if reported. T_{Br} = mean/median absolute T_{Br} (if reported) in °C.

[a]frontal lobe/thalamus

[b]superficial cortex/thalamus

[c]defined as upper threshold in healthy control subjects

[d]Water and N-acteylaspartate (NAA) solution

[e]Aqueous solutions of glycero-phosphocoline (GPC), creatine (Cr), and NAA

[f]Homogenous solution of metabolites

[g]pH-buffered aqueous solution of NAA, Cr, methyl protons of Cr (Cr2), dimethyl silapentane sulfonic acid (DSS), and sodium formate (NaFor)

Supplementary Table 2 Inclusion and exclusion criteria for prospective study

Criteria for inclusion	Criteria for exclusion
• Healthy men<br>• Healthy women with regular natural menstrual cycles for a minimum of 6 months, or women that have taken monophasic hormonal contraception (fixed dose oestradiol and synthetic progestin) for a minimum of 3 months prior to scanning and will continue to do so during the month of scanning.<br>• Age 20–40 years<br>• Body mass index 18.5–29.9<br>• Live within 5 mile radius of Edinburgh Imaging (Royal Infirmary of Edinburgh) Facility<br>• Capacity to understand written and verbal information provided in English, and able to provide valid written informed consent to participate<br>• Able to wear an actigraphy wristband for a week prior to scanning<br>• Able to commit to 3 x 45min scanning protocol within selected 24h period<br>• Able to commit to fixed times of food and caffeine consumption on the day of scanning<br>• Able to avoid alcohol and excessive physical activity on the day of scanning	• Pregnancy<br>• Early menopause, irregular menstrual cycles, premenstrual syndrome<br>• Lack of luteinizing hormone surge prior to two scheduled scanning days (naturally cycling women only)<br>• MRI contraindications (e.g. cardiac pacemaker, cochlear implant, claustrophobia, any prior accident in which metal penetrated one or both eyes)<br>• Oral temperature outside of normal range (33.2–38.1°C) prior to any scan<br>• Medical history that might limit activity or alter cerebral blood flow (hypothyroidism, stroke, severe arthritis, Parkinson's disease, dementia, history of brain trauma, brain tumour or epilepsy, significant mental illness besides clinical depression, spinal cord injuries, recent serious burn, diabetes, dehydration)<br>• Taking medications (except seasonal allergy medication, over-the-counter NSAIDs, or contraceptives), paracetamol, drug abuse<br>• Known neurodevelopmental, neuropsychiatric or neurodegenerative disorder<br>• Known sleep or chronotype disorder (delayed sleep phase syndrome, familial advanced sleep phase syndrome)<br>• Known family history of cardiovascular disease at < 40 years of age<br>• Failure to attend, or late attendance at, two independently scheduled morning scans<br>• Participant-reported ill-health on the day before or the morning of scanning

Supplementary Table 3 MRS linewidths by brain region, time of day, and sex

	**NAA**			**H_2O**		
	Morning	Afternoon	Evening	Morning	Afternoon	Evening
**Females**						
Cerebrum	7.44 (0.19)	7.31 (0.16)	7.31 (0.18)	9.70 (0.22)	9.45 (0.19)	9.40 (0.20)
Thalamus	8.64 (0.64)	8.46 (0.78)	8.46 (0.75)	11.5 (0.98)	11.3 (0.84)	11.3 (0.73)
Hypothalamus	10.8 (1.63)	10.0 (0.67)	9.86 (1.19)	14.1 (1.42)	14.0 (1.38)	13.9 (1.39)
**Males**						
Cerebrum	7.42 (0.29)	7.36 (0.27)	7.35 (0.28)	9.68 (0.34)	9.57 (0.35)	9.52 (0.31)
Thalamus	8.65 (0.58)	8.61 (0.62)	8.44 (0.71)	11.5 (0.92)	11.7 (0.85)	11.3 (0.69)
Hypothalamus	10.4 (1.44)	10.3 (1.57)	9.83 (0.88)	13.6 (1.48)	13.7 (1.27)	13.5 (1.48)

Spectral linewidth data presented for NAA and H_2O as mean (SD) for *n*=19 females and *n*=20 males. All data for the female participant with an HRF was excluded.

Supplementary Extended Data

A supplementary Excel spreadsheet is provided containing brain and body temperature rhythmicity analysis results for each TBI patient.

Captions for Supplementary Videos

Supplementary Video 1 Introduction to HEATWAVE. Dynamic 3D brain temperature map showing aggregate data from luteal females at one time point and highlighting brain regions from which data was extracted in healthy volunteers. Inferno colour scale is used to assign a temperature to each tissue voxel, to 0.1°C resolution. The colour of each voxel represents the mean raw temperature of that voxel calculated from the data obtained from all luteal females (*n*=14). For enhanced accessibility, the mapping reverses the radiological convention used in Fig. 3A such that from all perspectives, the data presented on the right side of the brain in HEATWAVE are from the right side of the human brain. Note that the thalamus is illuminated as a relatively hot, core brain structure.

Supplementary Video 2 HEATWAVE by sex. Comparison of diurnal brain temperature variation in luteal females and males (aggregate data from each group, without controlling for age, BMI, or chronotype), enabling both time-of-day and sex differences to be visualized in accelerated time, side-by-side. Two days' worth of data are presented in an accelerated looped fashion at 1 hour resolution. At the data collection points (9am, 4pm, 11pm), the colour of each voxel represents the mean raw temperature of that voxel calculated from the data obtained from either all luteal females (*n*=14), or all males (*n*=20). Between data collection points, data were extrapolated via non-linear regression of the aggregate data in GraphPad Prism version 8.2, fitting to a sine function (see Fig. 4D).

Supplementary references

1. Fischer D, Lombardi DA, Marucci-Wellman H, Roenneberg T. Chronotypes in the US ± Influence of age and sex. *PLoS One.* 2017;12:e0178782.
2. Cambras T, Castro-Marrero J, Zaragoza MC, Diez-Noguera A, Alegre J. Circadian rhythm abnormalities and autonomic dysfunction in patients with Chronic Fatigue Syndrome/Myalgic Encephalomyelitis. *PLoS One.* 2018;13:e0198106. doi:10.1371/journal.pone.0198106.
3. Blackwell T, Redline S, Ancoli-Israel S et al. Comparison of sleep parameters from actigraphy and polysomnography in older women: the SOF study. *Sleep.* 2008;31:283-291. doi:10.1093/sleep/31.2.283.
4. Marino M, Rueschman MN, Winkelman JW et al. Measuring sleep: accuracy, sensitivity and specificity of wrist actigraphy compared to polysomnography. *Sleep.* 2013;36:1747-1755. doi:10.5665/sleep.3142.
5. Albu S, Umemura G, Forner-Cordero A. Actigraphy-based evaluation of sleep quality and physical activity in individuals with spinal cord injury. *Spinal Cord Series and Cases.* 2019; 5:7. doi:10.1038/s41394-019-0149-0.
6. Cole RJ, Kripke DF, Gruen W, Mullaney DJ, Gillin JC. Automatic sleep/wake identification from wrist activity. *Sleep.* 1992;15:461-469.
7. Roenneberg T, Pilz LK, Zerbini G, Winnebeck EC. Chronotype and Social Jetlag: A (Self-) Critical Review. *Biology.* 2019;8:54. doi:10.3390/biology8030054.
8. Beale AD, Pedrazzoli M, Goncalves BdSB et al. Comparison between an African town and a neighbouring village shows delayed, but not decreased, sleep during the early stages of urbanisation. *Sci Rep.* 2017;7:5697.

DOI:10.1038/s41598-017-05712-3.
9. Jankowski KS. Social jet lag: Sleep-corrected formula. *Chronobiol Int.* 2017;34:531-535. doi: 10.1080/07420528.2017.1299162.
10. Thrippleton MJ, Parikh J, Harris B et al. Reliability of MRSI brain temperature mapping at 1.5 and 3 T. *NMR Biomed.* 2014;27:183-90.
11. Scheenen TWJ, Klomp DWJ, Wijnen JP, Heerschap A. Short echo time H-1-MRSI of the human brain at 3T with minimal chemical shift displacement errors using adiabatic refocusing pulses. *Magn Reson Med.* 2008;59:1-6.
12. Wilson M, Andronesi O, Barker PB et al. Methodological consensus on proton MRS of the brain: Review and recommendations. *Magn Resn Med.* 2019;82:527-550. doi: 10.1002/mrm.27742.
13. Steyerberg EW, Mushkudiani N, Perel P et al. Predicting outcome after traumatic brain injury: development and international validation of prognostic scores based on admission characteristics. *PLoS Med.* 2008;5:e165. doi:10.1371/journal.pmed.0050165.
14. Steyerberg EW, Moons KGM, van der Windt DA et al. Prognosis Research Strategy (PROGRESS) 3: Prognostic Model Research. *PLoS Med.* 2013;10:e1001381. doi:10.1371/journal.pmed.1001381.
15. Logan RW, McClung CA. Rhythms of life: circadian disruption and brain disorders across the lifespan. *Nat Rev Neurosci.* 2019;20:49-65.
16. Takahashi JS. Transcriptional architecture of the mammalian circadian clock. *Nat Rev Genet.* 2017;18:164-179.
17. Kondratova AA, Kondratov RV. The circadian clock and pathology of the ageing brain. *Nat Rev Neurosci.* 2012;13:325-335.
18. Roenneberg T, Merrow M. The circadian clock and human health. *Curr Biol.* 2016; 26:R432-443.
19. Nedergaard M, Goldman SA. Glymphatic failure as a final common pathway to dementia. *Science.* 2020;370:50-56.
20. GBD 2016 Neurology Collaborators. Global, regional, and national burden of neurological disorders, 1990–2016: a systematic analysis for the Global Burden of Disease Study 2016. *Lancet Neurol.* 2019;18:459-480.
21. Bekinschtein TA, Golombek DA, Simonetta SH, Coleman MR, Manes FF. Circadian rhythms in the vegetative state. *Brain Inj.* 2009;23:915-919.
22. C. Kirkness J, Burr RL, Thompson HJ, Mitchell PH. Temperature rhythm in aneurysmal subarachnoid haemorrhage. *Neurocrit Care.* 2008;8:380-390.
23. Paul T, Lemmer B. Disturbance of circadian rhythms in analgosedated intensive care unit patients with and without craniocerebral injury. *Chronobiol Int.* 2007;24:45-61.
24. Takekawa H, Miyamoto M, Miyamoto T, Yokota N, Hirata K. Alteration of circadian periodicity in core body temperature of patients with acute stroke. *Psychiatry Clin Neurosci.* 2002;56:221-222.
25. Lyall LM, Wyse CA, Graham N et al. Association of disrupted circadian rhythmicity with mood disorders, subjective wellbeing, and cognitive function: a cross-sectional study of 91 105 participants from the UK Biobank. *Lancet Psychiatry.* 2018;5:507-514.
26. Bunney BG, Walsh DM, Stein R et al. Circadian dysregulation of clock genes: clues to rapid treatments in major depressive disorder. *Mol Psychiatry.* 2015;20:48-55.
27. Leng Y, Musiek ES, Hu K, Cappuccio FP, Yaffe K. Association between circadian rhythms and neurodegenerative diseases. *Lancet Neurol.* 2019;18:307-318.
28. Khan S, Nobili L, Khatami R et al. Circadian rhythm and epilepsy. *Lancet Neurol.* 2018;17:1098-1108.
29. Nikitopoulou G, Crammer JL. Change in diurnal temperature rhythm in manic-depressive illness. *BMJ.* 1976;1:1311-1314.
30. Busto R, Dietrich WD, Globus MY, Valdes I, Scheinberg P, Ginsberg MD. Small differences in intraischemic brain temperature critically determine the extent of ischemic neuronal injury. *J Cereb Blood Flow Metab.* 1987;7:729-738.
31. Childs C, Vail A, Protheroe R, King AT, Dark PM. Differences between brain and rectal temperatures during routine critical care of patients with severe traumatic brain injury. *Anaesthesia.* 2005;60:759-76.
32. Abu-Arafeh A, Rodriguez A, Paterson RL, Andrews PJD. Temperature variability in a modern targeted temperature management trial. *Crit Care Med.* 2018;46:223-228.
33. Dehkhargani S, Fleischer CC, Qiu D, Yepes M, Tong F. Cerebral temperature dysregulation: MR thermographic monitoring in a nonhuman primate study of acute ischemic stroke. *AJNR Am J Neuroradiol.* 2017;38:712-720.
34. Rumana CS, Gopinath SP, Uzara M, Valadka AB, Robertson CS. Brain temperature exceeds systemic temperature in head-injured patients. *Crit Care Med.* 1998;26:562-567.

35. Karaszewski B, Wardlaw JM, Marshall I et al. Early brain temperature elevation and anaerobic metabolism in human acute ischaemic stroke. *Brain.* 2009;132:955-964.
36. Karaszewski B, Carpenter TK, Thomas RGR et al. Relationships between brain and body temperature, clinical and imaging outcomes after ischemic stroke. *J Cereb Blood Flow Metab.* 2013;33:1083-1089.
37. Verius M, Frank F, Gizewski E, Broessner G. Magnetic resonance spectroscopy thermometry at 3 Tesla: importance of calibration measurements. *Ther Hypothermia Temp Manag.* 2019;9:146-155.
38. Nanba T, Nishimoto H, Yoshioka Y et al. Apparent brain temperature imaging with multi-voxel proton magnetic resonance spectroscopy compared with cerebral blood flow and metabolism imaging on positron emission tomography in patients with unilateral chronic major artery steno-occlusive disease. *Neuroradiology.* 2017;59:923-935.
39. Tsutsui S, Nanba T, Yoshioka Y et al. Preoperative brain temperature imaging on proton magnetic resonance spectroscopy predicts hemispheric ischemia during carotid endarterectomy for unilateral carotid stenosis with inadequate collateral blood flow. *Neurol Res.* 2018;40:617-623.
40. Marshall I, Karaszewski B, Wardlaw JM et al. Measurement of regional brain temperature using proton spectroscopic imaging: validation and application to acute ischemic stroke. *Magn Reson Imaging.* 2006;24:699-706.
41. Rango M, Arighi A, Bonifati C, Del Bo R, Comi G, Bresolin N. The brain is hypothermic in patients with mitochondrial diseases. *J Cereb Blood Flow Metab.* 2014;34:915-920.
42. Sone D, Ikegaya N, Takahashi A et al. Noninvasive detection of focal brain hyperthermia related to continuous epileptic activities using proton MR spectroscopy. *Epilepsy Res.* 2017;138:1-4.
43. Shiloh R, Kushnir T, Gilat Y et al. In vivo occipital-frontal temperature-gradient in schizophrenia patients and its possible association with psychopathology: a magnetic resonance spectroscopy study. *Eur Neuropsychopharmacol.* 2008;18:557-564.
44. Posporelis S, Coughlin JM, Marsman A et al. Decoupling of brain temperature and glutamate in recent onset of schizophrenia: a 7T proton Magnetic Resonance Spectroscopy study. *Biol Psychiatry Cogn Neurosci Neuroimaging.* 2018;3:248-254.
45. Covaciu L, Rubertsson S, Ortiz-Nieto F, Ahlström H, Weis J. Human brain MR spectroscopy thermometry using metabolite aqueous-solution calibrations. *J Magn Reson Imaging.* 2010;31:807-814.
46. Maudsley AA, Goryawala MZ, Sheriff S. Effects of tissue susceptibility on brain temperature mapping. *Neuroimage.* 2017;146:1093-1101.
47. Chadzynski GL, Bender B, Groeger A, Erb M, Klose U. Tissue specific resonance frequencies of water and metabolites within the human brain. *J Magn Reson.* 2011;212:55-63.
48. Brown MA. Time-domain combination of MR spectroscopy data acquired using phased-array coils. *Magn Reson Med.* 2004;52:1207-1213.
49. Maloney SK, Mitchell D, Mitchell G, Fuller A. Absence of selective brain cooling in unrestrained baboons exposed to heat. *Am J Physiol Regul Integr Comp Physiol.* 2007;292: R2059-2067.
50. Hayward JN, Baker MA. Role of cerebral arterial blood in the regulation of brain temperature in the monkey. *Am J Physiol.* 1968;215:389-403.
51. Prakash KN, Verma SK, Marchenko Y et al. Echo planar spectroscopic imaging based temperature calibration at 7T and 3T for whole brain temperature measurement in rodents and humans. *International Society of Magnetic Resonance in Medicine, Milan 2014* (conference paper).
52. Fuller CA, Baker MA. Selective regulation of brain and body temperatures in the squirrel monkey. *Am J Physiol.* 1983;245:R293-R297.
53. Shiraki K, Sagawa S, Tajima F, Yokota A, Hashimoto M, Brengelmann GL. Independence of brain and tympanic temperatures in an unanaesthetised human. *J Appl Physiol. (1985)* 1988;65:482-486.
54. Baker FC, Waner JI, Vieira EF, Taylor SR, Driver HS, Mitchell D. Sleep and 24 hour body temperatures: a comparison in young men, naturally cycling women and women taking hormonal contraceptives. *J Physiol.* 2001;530:565-574.
55. Papaionnou VE, Chouvarda IG, Maglaveras NK, Pneumatikos IA. Temperature variability analysis using wavelets and multiscale entropy in patients with systemic inflammatory response syndrome, sepsis, and septic shock. *Crit Care Lond Engl.* 2012;16:R51.
56. Mariak Z. Intracranial temperature recordings in human subjects. The contribution of the neurosurgeon to thermal physiology. *J Therm Biol.* 2002;27:219-228. https://doi.org/10.1016/S0306-4565(01)00087-0.
57. Walsh JJ, Huang Y, Simmons JW et al. Dynamic thermal mapping of localized therapeutic hypothermia in the brain. *J Neurotrauma.* 2020;37:55-65.
58. Erikson R. Oral temperature differences in relation to thermometer and technique. *Nurs Res.* 1980;29:157-

64.
59. Childs C, Hiltunen Y, Vidyasagar R, Kaupinnen RA. Determination of regional brain temperature using proton magnetic resonance spectroscopy to assess brain-body temperature differences in healthy human subjects. *Magn Reson Med.* 2007;57:59-66.
60. Corbett R, Laptook A, Weatherall P. Noninvasive measurements of human brain temperature using volume-localized proton magnetic resonance spectroscopy. *J Cereb Blood Flow Metab.* 1997;17:363-369.
61. Awaya S. A study of brain temperature in patients with severe head injuries. *Nihon Ika Daigaku Zasshi.* 1993;60:37-43.
62. Brengelmann GL. Specialised brain cooling in humans? *FASEB J.* 1993;7:1148-1153.
63. Cabanac M. Selective brain cooling in humans: "fancy" or fact? *FASEB J.* 1993;7:1143-1147.
64. Refinetti R. The circadian rhythm of body temperature. *Front Biosci (Landmark Ed).* 2010;15:564-594.
65. Rango M, Arighi A. Bonifati C, Del Bo R, Comi G, Bresolin N. The brain is hypothermic in patients with mitochondrial diseases. *J Cereb Blood Flow Metab.* 2014;34:915-920.
66. Orita T, Izumihara A, Tsurutani T, Kajiwara K. Brain temperature before and after brain death. *Neurol Res.* 1995;17:443-444.
67. Sen A, Jette N, Husain M, Sander JW. Epilepsy in older people. *Lancet.* 2020;395:735-748.
68. Peretti D, Bastide A, Radford H et al. RBM3 mediates structural plasticity and protective effects of cooling in neurodegeneration. *Nature.* 2015;518:236-239.
69. Sandsmark DK, Bashir A, Wellington CL, Diaz-Arrastia R. Cerebral microvascular injury: a potentially treatable endophenotype of traumatic brain injury-induced neurodegeneration. *Neuron.* 2019;103:367-379.
70. Rzechorzek NM, Connick P, Livesey MR et al. Hypothermic preconditioning reverses tau ontogenesis in human cortical neurons and is mimicked by protein phosphatase 2A inhibition. *EBioMedicine.* 2015;3:141-154.
71. Spillantini MG, Goedert M. Tau pathology and neurodegeneration. *Lancet Neurol.* 2013;12: 609-622.
72. Mirmiran M, Bernardo L, Jenkins SL, Ma XH, Brenna JT, Nathanielsz PW. Growth, neurobehavioural and circadian rhythm development in newborn baboons. *Pediatr Res.* 2001; 49:673-677.
73. Tjijs RD, Surges R, O'Brien TJ, Sander W. Epilepsy in adults. *Lancet.* 2019;393:689-701.
74. Arendt T, Stieler J, Strijkstra AM et al. Reversible paired helical filament-like phosphorylation of tau is an adaptive process associated with neuronal plasticity in hibernating animals. *J Neurosci.* 2003;23:6972-6981.
75. Mariak Z, White MD, Lewko J, Lyson T, Piekarski P. Direct cooling of the human brain by heat loss from the upper respiratory tract. *J Appl Physiol (1985).* 1999;87:1609-1613.
76. Roberge RJ, Kim JH, Coca A. Protective facemask impact on human thermoregulation: an overview. *Ann Occup Hyg.* 2012;56:102-112.
77. Kuwabara N, Seki K, Aoki K. Circadian, sleep and brain temperature rhythms in cats under sustained daily light-dark cycles and constant darkness. *Physiol Behav.* 1986;38,283-289.
78. J. Aschoff, Circadian rhythms in man. *Science.* 1965;148:1427-1432.
79. Daan S, Honma S, Honma K. Body temperature predicts the direction of internal desynchronization in humans isolated from time cues. *J Biol Rhythms.* 2013;28:403-411.
80. Archer SN, Oster H. How sleep and wakefulness influence circadian rhythmicity: effects of insufficient and mistimed sleep on the animal and human transcriptome. *J Sleep Res.* 2015;24: 476-493.
81. Dijk DJ. Circadian variation of EEG power spectra in NREM and REM in humans: dissociation from body temperature. *J Sleep Res.* 1999;8:198-195.
82. Baker FC, Angara C, Szymusiak R, McGinty D. Persistence of sleep-temperature after suprachiasmatic nuclei lesions in rats. *Am J Physiol Integr Comp Physiol.* 2005;289:R927-838.
83. V. Skop V, Guo J, Liu N et al. Mouse thermoregulation: introducing the concept of the thermoneutral point. *Cell Rep.* 2020;31:107501. doi: 10.1016/j.celrep.2020.03.065.
84. Frank MG. Circadian regulation of synaptic plasticity. *Biology (Basel).* 2016;5:pii:E31 doi: 10.3390/biology5030031.
85. Deboer T. Brain temperature dependent changes in the electroencephalogram power spectrum of humans and animals. *J Sleep Res.* 1998;7:254-262.
86. Cai X, Qiao J, Kulkarni P, Harding IC, Ebong E, Ferris CF. Imaging the effect of the circadian light-dark cycle on the glymphatic system in awake rats. *Proc Natl Acad Sci USA.* 2020;117:668-676.
87. Hastings MH, O'Neill JS, Maywood ES. Circadian clocks: regulators of endocrine and metabolic rhythms. *J Endocrinol.* 2007;195:187-98.
88. Conroy DA, Spielman AJ, Scott RQ. Daily rhythm of cerebral blood flow velocity. *J Circadian Rhythms.* 2005;3:3. doi:10.1186/1740-3391-3-3.

89. Aschoff VJ, Wever R. Spontanperiodik des menschen bei ausschluß aller zeitgeber. *Naturwissenschaften.* 1962;49:337-342.
90. Harding EC, Franks NP, Wisden W. The temperature dependence of sleep. *Front Neurosci.* 2019;23:336.
91. Raymann RJ, Swaab DF, Van Someren EJ. Cutaneous warming promotes sleep onset. *Am J Physiol Regul Integr Comp Physiol.* 2005;288:R1589-R1597.
92. Cook JS, Sauder CL, Ray CA. Melatonin differentially affects vascular blood flow in humans. *Am J Physiol Heart Circ Physiol.* 2011;300:H670-H674.
93. Maas MB, Lizza BD, Abbott AM. Factors disrupting melatonin secretion rhythms during critical illness. *Crit Care Med.* 2020;48:854-861.
94. Blume C, Lechinger J, Santhi N et al. Significance of circadian rhythms in severely brain-injured patients: a clue to consciousness? *Neurology.* 2017;88:1933-1941.
95. Culver A, Coiffard B, Antonini F et al. Circadian disruption of core body temperature in trauma patients: a single-center retrospective observational study. *J Intensive Care.* 2020;8:4. doi: 10.1186/s40560-019-0425-x.
96. Niven DJ, Gaudet JE, Laupland KB et al. Accuracy of peripheral thermometers for estimating temperature: a systematic review and meta-analysis. *Ann Intern Med.* 2015;163:768-777.
97. Van Someren EJ. More than a marker: interaction between the circadian regulation of temperature and sleep, age-related changes, and treatment possibilities. *Chronobiol Int.* 2000;17:313-354. doi: 10.1081/cbi-100101050.
98. Corbett RJ, Laptook AR. Failure of localized head cooling to reduce brain temperature in adult humans. *Neuroreport.* 1998;9:2721-2725.
99. Curran EJ, Wolfson DL, Watts R, Freeman K. Cold blooded: evaluating brain temperature by MRI during surface cooling of human subjects. *Neurocrit Care.* 2017;27:214-219.
100. Covaciu L. Brain temperature in volunteers subjected to intranasal cooling. *Intensive Care Med.* 2011;37:1277-1284.
101. Weis J, Covaciu L, Rubertsson S et al. Noninvasive monitoring of brain temperature during mild hypothermia. *Magn Reson Imaging.* 2009;27:923-932.
102. Fujiwara S, Yoshika Y, Matsuda T et al. Brain temperature measured by 1H-magnetic resonance spectroscopy in acute and subacute carbon monoxide poisoning. *Neuroradiology.* 2016;58:27-32.
103. Cady EB, D'Souza PC, Penrice J, Lorek A. The estimation of local brain temperature by in vivo 1H magnetic resonance spectroscopy. *Magn Reson Med.* 1995;33:862-867.
104. Harris BA, Andrews PJ, Marshall I, Robinson TM, Murray GD. Forced convective head cooling device reduces human cross-sectional brain temperature measured by magnetic resonance: a non-randomized healthy volunteer pilot study. *Br J Anaesth.* 2008;100:365-72.
105. Inoue T, Shimizu H, Fujimura M et al. Noninvasive measurement of human brain temperature adjacent to arteriovenous malformation using 3.0T magnetic resonance spectroscopy. *Clin Neurol Neurosurg.* 2013;115:445-449.
106. Birkl C, Langkammer C, Haybaeck J et al. Temperature dependency of T1 relaxation time in unfixed and fixed human brain tissue. *Biomed Tech (Berl).* 2013;58 Suppl 1.pii/j/bmte.2013.58.issue-s1-L/bmt-2013-4290/bmt-2013-4290.xml. doi: 10.1515/bmt-2013-4290.
107. Kickhefel A, Roland J, Weiss C, Schick F. Accuracy of real-time MR temperature mapping in the brain: a comparison of fast sequences. *Phys Med.* 2010;26:192-201.
108. Cady EB, Penrice J, Robertson NJ. Improved reproducibility of MRS regional brain thermometry by 'amplitude-weighted combination'. *NMR Biomed.* 2011;24:865-872.
109. Zhu M, Bashir A, Ackerman JJ, Yablonskiy DA. Improved calibration technique for in vivo proton MRS thermometry for brain temperature measurement. *Magn Reson Med.* 2008;60:536-541.
110. Onitsuka S, Nakamura D, Onishi T, Arimitsu T, Takahashi H, Hasegawa H. Ice slurry ingestion reduces human brain temperature measured using non-invasive magnetic resonance spectroscopy. *Sci Rep.* 2018;8:2757. doi: 10.1038/s41598-018-21086-6.
111. Rango M, Bonifati C, Bresolin N. Post-activation brain warming: a thermometry study. *PLoS One.* 2015;10:e0127314.
112. Sharma AA, Nenert R, Mueller C, Maudsley AA, Younger JW, Szaflarski JP. Repeatability and reproducibility of in-vivo brain temperature measurements. *Front Hum Neurosci.* 2020;14:598435. doi: 10.3389/fnhum.2020.598435.

113. Shiloh R, et al., In vivo occipital-frontal temperature-gradient in schizophrenia patients and its possible association with psychopathology: a magnetic resonance spectroscopy study. *Eur. Neuropsychopharmacol.* 2008;18:557–564.
114. Sumida K, et al. Intraventricular temperature measured by diffusion-weighted imaging compared with brain parenchymal temperature measured by MRS in vivo. *NMR Biomed.* 2016;29:890–895.
115. Weis J, Covaciu L, Rubertsson S et al. Phase-difference and spectroscopic imaging for monitoring of human brain temperature during cooling. *Magn Reson Imaging.* 2012;30:1505-1511.
116. Zhang Y, Taub E, Mueller C, Younger J, Uswatte G, DeRamus TP, Knight DC. Reproducibility of whole-brain temperature mapping and metabolite quantification using proton magnetic spectroscopy. *NMR Biomed.* 2020;33:e4313. https://doi.org/10.1002/nbm.4313.

Supplementary Appendices

Supplementary Appendix 1 - Participant Information Sheet and Consent to Participate Form
Supplementary Appendix 2 - Prospective Study Protocol
Supplementary Appendix 3 - MRI Protocol
Supplementary Appendix 4 - Study Participant Data Form
Supplementary Appendix 5 - Analytic code for statistical models
