# Supplementary material for: A daily temperature rhythm in the human brain predicts survival after brain injury
Source: Brain. 2022 Jun 13;145(6):2031–48. doi: 10.1093/brain/awab466 (PMC9336587; doi:10.1093/brain/awab466)
Supplement: awab466_Supplementary_Data [file awab466_Supplementary_Data.zip › brain-2021-00914-File019.pdf]

Investigators for The CENTER-TBI HR ICU Sub-Study include: Audny Anke, Department of Physical Medicine and Rehabilitation, University Hospital of North Norway, Tromsø, Norway; Ronny Beer, Department of Neurology, Neurological Intensive Care Unit, Medical University of Innsbruck, Innsbruck, Austria; Bo-Michael Bellander, Department of Neurosurgery & Anesthesia & intensive care medicine, Karolinska University Hospital, Stockholm, Sweden; Erta Beqiri, NeuroIntensive Care, Niguarda Hospital, Milan, Italy; Andras Buki, Department of Neurosurgery, Medical School, University of Pécs, Hungary and Neurotrauma Research Group, János Szentágothai Research Centre, University of Pécs, Hungary; Manuel Cabeleira, Brain Physics Lab, Division of Neurosurgery, Dept of Clinical Neurosciences, University of Cambridge, Addenbrooke's Hospital, Cambridge, UK; Marco Carbonara, Neuro ICU, Fondazione IRCCS Cà Granda Ospedale Maggiore Policlinico, Milan, Italy; Giorgio Chevallard, NeuroIntensive Care, Niguarda Hospital, Milan, Italy; Arturo Chieregato, NeuroIntensive Care, Niguarda Hospital, Milan, Italy; Giuseppe Citerio, NeuroIntensive Care Unit, Department of Anesthesia & Intensive Care, ASST di Monza, Monza, Italy; and School of Medicine and Surgery, Università Milano Bicocca, Milano, Italy; Endre Czeiter, Department of Neurosurgery, University of Pécs and MTA-PTE Clinical Neuroscience MR Research Group and János Szentágothai Research Centre, University of Pécs, Hungarian Brain Research Program (Grant No. KTIA 13 NAP-A-II/8), Pécs, Hungary; Marek Czosnyka, Brain Physics Lab, Division of Neurosurgery, Dept of Clinical Neurosciences, University of Cambridge, Addenbrooke's Hospital, Cambridge, UK; Bart Depreitere, Department of Neurosurgery, University Hospitals Leuven, Leuven, Belgium; Ari Ercole, Division of Anaesthesia, University of Cambridge, Addenbrooke's Hospital, Cambridge, UK; Shirin Frisvold, Department of Anesthesiology and Intensive Care, University Hospital of North Norway, Tromsø, Norway; Raimund Helbok, Department of Neurology, Neurological Intensive Care Unit, Medical University of Innsbruck, Innsbruck, Austria; Stefan Jankowski, Neurointensive Care, Sheffield Teaching Hospitals NHS Foundation Trust, Sheffield, UK; Daniel Kondziella, Departments of Neurology, Clinical Neurophysiology and Neuroanesthesiology, Region Hovedstaden Rigshospitalet, Copenhagen, Denmark; Lars-Owe Koskinen, Department of Clinical Neuroscience, Neurosurgery, Umeå University, Umeå, Sweden; David K. Menon, Division of Anaesthesia, University of Cambridge, Addenbrooke's Hospital, Cambridge, UK; Geert Meyfroidt, Intensive Care Medicine, University Hospitals Leuven, Leuven, Belgium; Kirsten Moeller, Department Neuroanesthesiology, Region Hovedstaden Rigshospitalet, Copenhagen, Denmark; David Nelson, Department of Neurosurgery & Anesthesia & intensive care medicine, Karolinska University Hospital, Stockholm, Sweden; Anna Piippo-Karjalainen, Helsinki University Central Hospital, Helsinki, Finland; Andreea Radoi, Department of

Neurosurgery, Vall d'Hebron University Hospital, Barcelona, Spain; Arminas Ragauskas, Department of Neurosurgery, Kaunas University of Technology and Vilnius University, Vilnius, Lithuania; Rahul Raj, Helsinki University Central Hospital, Helsinki, Finland; Jonathan Rhodes, Department of Anaesthesia, Critical Care & Pain Medicine, NHS Lothian & University of Edinburgh, Edinburgh, UK; Saulius Rocka, Department of Neurosurgery, Kaunas University of Technology and Vilnius University, Vilnius, Lithuania; Rolf Rossaint, Department of Anaesthesiology, University Hospital of Aachen, Aachen, Germany; Juan Sahuquillo, Department of Neurosurgery, Vall d'Hebron University Hospital, Barcelona, Spain; Oliver Sakowitz, Klinik für Neurochirurgie, Klinikum Ludwigsburg, Ludwigsburg, Germany; Department of Neurosurgery, University Hospital Heidelberg, Heidelberg, Germany; Peter Smielewksi, Brain Physics Lab, Division of Neurosurgery, Dept of Clinical Neurosciences, University of Cambridge, Addenbrooke's Hospital, Cambridge, UK; Ana Stevanovic, Department of Anaesthesiology, University Hospital of Aachen, Aachen, Germany; Nino Stocchetti, Department of Pathophysiology and Transplantation, Milan University, and Neuroscience ICU, Fondazione IRCCS Cà Granda Ospedale Maggiore Policlinico, Milano, Italy; Nina Sundström, Department of Radiation Sciences, Biomedical Engineering, Umea University Hospital, Umea, Sweden; Riikka Takala, Perioperative Services, Intensive Care Medicine, and Pain Management, Turku University Central Hospital and University of Turku, Turku, Finland; Tomas Tamosuitis, Neuro-intensive Care Unit, Kaunas University of Health Sciences, Kaunas, Lithuania; Olli Tenovuo, Rehabilitation and Brain Trauma, Turku University Central Hospital and University of Turku, Turku, Finland; Peter Vajkoczy, Neurologie, Neurochirurgie und Psychiatrie, Charité–Universitätsmedizin Berlin, Berlin, Germany; Alessia Vargiolu, NeuroIntensive Care Unit, Department of Anesthesia & Intensive Care, ASST di Monza, Monza, Italy; Rimantas Vilcinis, Department of Neurosurgery, Kaunas University of Health Sciences, Kaunas, Lithuania; Stefan Wolf, Interdisciplinary Neuro Intensive Care Unit, Charité–Universitätsmedizin Berlin, Berlin, Germany; Alexander Younsi, Department of Neurosurgery, University Hospital Heidelberg, Heidelberg, Germany; Frederick A. Zeiler, Division of Anaesthesia, University of Cambridge, Addenbrooke's Hospital, Cambridge, UK; and Section of Neurosurgery, Department of Surgery, Rady Faculty of Health Sciences, University of Manitoba, Winnipeg, MB, Canada.
